# Risk Factors for Chronic Cerebrospinal Venous Insufficiency (CCSVI) in a Large Cohort of Volunteers

**DOI:** 10.1371/journal.pone.0028062

**Published:** 2011-11-30

**Authors:** Kresimir Dolic, Bianca Weinstock-Guttman, Karen Marr, Vesela Valnarov, Ellen Carl, Jesper Hagemeier, Christina Brooks, Colleen Kilanowski, David Hojnacki, Murali Ramanathan, Robert Zivadinov

**Affiliations:** 1 Buffalo Neuroimaging Analysis Center, University at Buffalo, State University of New York, Buffalo, New York, United States of America; 2 Department of Neurology, The Jacobs Neurological Institute, Kaleida Health, University at Buffalo, State University of New York, Buffalo, New York, United States of America; 3 Department of Pharmaceutical Sciences, University at Buffalo, State University of New York, Buffalo, New York, United States of America; University of Modena and Reggio Emilia, Italy

## Abstract

**Background:**

The role of intra- and extra-cranial venous system impairment in the pathogenesis of various vascular, inflammatory and neurodegenerative neurological disorders, as well as in aging, has not been studied in detail. Nor have risk factors been determined for increased susceptibility of venous pathology in the intra-cranial and extra-cranial veins. The aim of this study was to investigate the association between presence of a newly proposed vascular condition called chronic cerebrospinal venous insufficiency (CCSVI) and environmental factors in a large volunteer control group without known central nervous system pathology.

**Methods and Findings:**

The data were collected in a prospective study from 252 subjects who were screened for medical history as part of the entry criteria and participated in the case-control study of CCSVI prevalence in multiple sclerosis (MS) patients, and then were analyzed post-hoc. All participants underwent physical and Doppler sonography examinations, and were assessed with a structured environmental questionnaire. Fullfilment of ≥2 positive venous hemodynamic (VH) criteria on Doppler sonography was considered indicative of CCSVI diagnosis. Risk and protective factors associated with CCSVI were analyzed using logistic regression analysis. Seventy (27.8%) subjects presented with CCSVI diagnosis and 153 (60.7%) presented with one or more VH criteria. The presence of heart disease (p = .001), especially heart murmurs (p = .007), a history of infectious mononucleosis (p = .002), and irritable bowel syndrome (p = .005) were associated with more frequent CCSVI diagnosis. Current or previous smoking (p = .029) showed a trend for association with more frequent CCSVI diagnosis, while use of dietary supplements (p = .018) showed a trend for association with less frequent CCSVI diagnosis.

**Conclusions:**

Risk factors for CCSVI differ from established risk factors for peripheral venous diseases. Vascular, infectious and inflammatory factors were associated with higher CCSVI frequency.

## Introduction

Several studies have shown a relationship between internal jugular vein (IJV) drainage abnormalities and specific neurological diseases of undetermined etiology such as transient global amnesia [1], transient monocular blindness [2], cough headache [3], primary exertional headache [4], idiopathic intra-cranial hypertension [5] and higher prevalence of white matter hyperintensities in elderly people.[6] The role of intra- and extra-cranial venous system impairment in the pathogenesis of various vascular, inflammatory and neurodegenerative neurological disorders, as well as in aging, has not been studied in detail. Nor have risk factors been determined for increased susceptibility of venous pathology in the intra-cranial and extra-cranial veins. Certainly, physiological inter-individual variation of the cerebral venous anatomy and the complexity of imaging the venous systems, regardless of the modality used, contribute to the lack of risk factor studies in relation to the intra- and extra-cranial venous system.

Recently, a new vascular condition called chronic cerebrospinal venous insufficiency (CCSVI) was proposed in patients with multiple sclerosis (MS) [7]. CCSVI is characterized by impaired blood outflow from the central nervous system (CNS) to the periphery, secondary to anatomical abnormalities of the major neck and azygos veins [7]. So far, CCSVI has caused considerable controversy and debate in the medical literature because: 1) recently published studies [8]–[14] failed to reproduce the original results that reported that CCSVI was never observed in controls, but perfectly overlapped with the diagnosis of MS [7]; 2) of uncritical application of interventional procedures in the treatment of CCSVI-related abnormalities without established safety and efficacy outcomes [15]–[17], and 3) it has been demonstrated that CCSVI-related abnormalities are not exclusive to MS patients, but healthy controls [11], [13], [18] or patients with other neurological diseases [11] can also present with these anomalies.

The origin of CCSVI-related venous anomalies has not been determined. It has been suggested that the origin of these abnormalities could be physiological [4], [8], aging-dependent [6], [19], congenital [20], a possible consequence of an inflammatory process [11], related to chronic pulmonary pathology such as chronic obstructive pulmonary disease (COPD) and pulmonary hypertension [21] or related to environmental factors.

In the first phase of the Combined Trans-cranial and Extra-cranial Venous Doppler (CTEVD) study that enrolled 499 participants [11], CCSVI prevalence rates were 56.1% in MS patients, 42.3% in those with other neurologic diseases, 38.1% in clinically isolated syndrome (CIS) patients and 22.7% in controls. Another recent study showed even higher prevalence of CCSVI in controls (36%) [18].

Therefore, the aim of this study was to investigate the association between presence of CCSVI and risk/protective factors in a large volunteer control group without known central nervous system (CNS) pathology.

## Methods

### Subjects and clinical assessments

The study participants were controls without known CNS pathology who were part of the prospective CTEVD study and were analyzed post-hoc [11]. The study started in April 2009 and is still enrolling controls, as well as patients with MS, CIS and with other neurologic diseases. Controls were recruited from the following volunteer sources: hospital personnel, respondents to a local newspaper advertisement, and spouses or relatives of the MS patients.

Inclusion criteria were: fulfilling health screen questionaire requirements containing information about medical history (illnesses, surgeries, medications, etc.), fulfilling the health screen requirements on physical examination, being capable of undergoing diagnostic evaluation for intra- and extra-cranial venous system using Doppler sonography (DS) and being able to respond on a structured environmental questionnaire. Exclusion criteria included pre-existing medical conditions known to be associated with brain pathology (e.g., neurodegenerative disorder, cerebrovascular disease, cognitive impairment, history of psychiatric disorders, seizures, trauma, etc.), neck pathology, history of cerebral congenital vascular malformations, venous thrombosis, genetic thrombophilia and presence of arthritic necks (as these subjects may not be able to lie flat).

All subjects underwent physical and DS examinations, and were assessed with a structured environmental questionnaire administered in-person by a trained interviewer unaware of subjects' disease status (Appendix S1). The questionnaire contained information related to demographic characteristics, presence of autoimmune and other concomitant diseases, vascular risk factors and environmental factors, as well as information about habits (Appendix S2). Race/ethnicity was determined according to the US Census Bureau.

This study was approved by the local Health Sciences Institutional Review Board (HSIRB #NEU2490109A) and written informed consent was obtained from all subjects.

### Doppler sonography

Participants underwent trans- and extra-cranial DS of the neck. A Color-coded DS scanner (Esaote-Biosound, My lab 25) equipped with a 7.5–10 Mhz transducer was used to examine venous return in the IJVs and vertebral veins (VVs). The DS examination was performed by two trained technologists who were blinded to subjects' characteristics, as previously reported [11]. The detailed scanning protocol and validation were recently reported [11]. Briefly, the following 5 venous hemodynamic (VH) parameters indicative of CCSVI were investigated: 1) Reflux/bidirectional flow in the IJVs and/or in the VVs in sitting and in supine positions, defined as flow directed towards the brain for a duration of >0.88 s; 2) Reflux/bidirectional flow in the deep cerebral veins defined as reverse flow for a duration of 0.5 s in one of the intra-cranial veins; 3) B-mode abnormalities or stenoses in IJVs. IJV stenosis is defined as a cross-sectional area (CSA) of this vein ≤0.3 cm2; 4) Flow that is not Doppler-detectable in IJVs and/or VVs despite multiple deep breaths, and 5) Reverted postural control of the main cerebral venous outflow pathway by measuring the difference of the CSA of the IJVs in the supine and upright positions. A subject was considered CCSVI-positive if ≥2 VH criteria were fulfilled, as previously proposed [7].

### Statistical analyses

Statistical analysis was performed using the Statistical Package for Social Sciences (SPSS, version 16.0). Student's *t*-test and the chi-square test were used for comparing demographic and clinical differences between the study subgroups, as appropriate. In order to test whether CCSVI was dependent on age, the subjects were divided into age groups, ranging from 0–10 years to 71–80 years, and the differences were tested with the chi-square test. Individual risk factors were investigated separately. Whenever possible, the presence of multiple variables that investigated specific individual risk factors was combined into a single variable (Appendix S2). Logistic regression analysis was used to test which risk/protective factors were associated with CCSVI diagnosis. The odds-ratio (OR) and a 95% confidence interval (CI) were calculated.

To correct for multiple comparisons, a nominal p-value of <0.01 was considered significant using two-tailed tests.

## Results

### Demographic and clinical characteristics

Table 1 shows demographic and clinical characteristics of the study subjects. The mean age of subjects was 42.4±15.3 years, median was 44.5 years with a range of 60. One hundred forty-four subjects were females (57.1%), and 82 (32.1%) of all participants had a relative with MS diagnosis. No significant differences were found in number, age and gender between participants, who were selected from different recruitment sources (hospital personnel, respondents to advertisement, MS spouses or MS relatives).

**Table 1:** Demographic and clinical characteristics in study subjects.

	Subjectsn (%)	Femalen (%)	Age years Mean (SD)
**Population**	252	144 (57.1)	42.4 (15.3)
**Race/Ethnicity**			
Caucasian	207 (82.1)	109 (53.4)	43.8 (15.4)
African-American	16 (6.3)	12 (66.7)	37.3 (10)
Hispanic/Latino	2 (0.8)	1 (50)	28 (14.1)
Asian	4 (1.6)	3 (75)	33.5 (19.1)
Other/missing	23 (9.1)	19 (13.2)	35.7 (11)
**Vascular risk factors**			
Heart disease	80 (31.7)	45 (31.3)	43.3 (15.2)
Smoking	146 (57.9)	35 (24.3)	41.9 (15.4)
Hypertension	64 (25.4)	29 (20.1)	48.6 (14.9)
Obesity	56 (22.2)	34 (23.6)	46 (13.5)
**Habits**			
Alcohol	204 (81)	112 (54.9)	44.2 (13.7)
Dietary supplements	144 (57.1)	90 (62.5)	44.4 (15.04)
Herbal supplements	39 (15.5)	26 (66.7)	41.8 (12.6)
Physical activity	168 (66.7)	87 (60.4)	40.6 (15.9)
**Autoimmune diseases**			
Rheumatoid disorders	8 (3.2)	4 (50)	52.1 (7.1)
Psoriasis	6 (2.4)	4 (66.6)	49.2 (17.3)
Diabetes Mellitus Type 1	5 (2)	3 (60)	51.5 (5.2)
**Other diseases**			
Asthma	32 (12.7)	22 (68.7)	43.7 (16.6)
Allergy	90 (35.7)	62 (68.8)	42.6 (15.7)
Cancer	14 (5.7)	7 (50)	53.7 (6.4)
COPD	9 (3.6)	7 (77.7)	52 (11.9)
Irritable bowel syndrome	19 (7.5)	11 (57.9)	45.9 (12.06)
Migraine	40 (15.9)	28 (70)	42.4 (13.5)

**Legend:** COPD – chronic obstructive pulmonary disease.

The various environmental factor frequencies were calculated on the total population of 252 subjects. Data for missing cases were conservatively categorized as negative.

Of the 252 participants, 207 (82.1%) were Caucasian, 16 (6.3%) were African-Americans, 4 (1.6%) were Asian, 2 (0.8%) were Hispanic/Latino and data for 23 subjects were missing. The majority of the participants (over 95%) were from Buffalo, NY.

Almost 1/3 of the subjects presented with one or more vascular risk factors (ranging from 22.2% to 57.9%) and almost half of the subjects (57.1%) were taking dietary supplements. Consumption of alcohol (ranging from occasionally to daily) was very frequent among participants (81%). Almost 2/3 of subjects practiced physical activity (ranging from daily to weekly). Only 9 subjects reported an autoimmune disease. Allergy was present in 35.7% of participants and migraine in 15.9%.

### CCSVI prevalence

Table 2 shows CCSVI prevalence in study subjects and the gender differences for global and individual VH criteria. Seventy (27.8%) subjects presented with ≥2 positive VH criteria and were considered to have CCSVI. One or more positive VH criteria were found in 153 (60.7%) participants. The most common positive VH criterion was criterion 3 (B-mode abnormalities or proximal stenoses in IJVs), which was present in 99 (39.3%) of the subjects, followed by VH criterion 2 (reflux/bidirectional flow in the DCVs), (20.2% of subjects).

**Table 2:** CCSVI prevalence in study subjects on Doppler sonography.

CCSVI criteria	Total (n = 252)	Females (n = 144)	Males (n = 108)	p
VH Criterion 1, n (%)	36 (14.3)	18 (12.5)	18 (16.7)	.225
VH Criterion 2, n (%)	51(20.2)	31 (21.5)	20 (18.5)	.335
VH Criterion 3, n (%)	99 (39.3)	51 (35.4)	48 (44.4)	.093
VH Criterion 4, n (%)	31(12.3)	14 (9.7)	17 (15.7)	.107
VH Criterion 5, n (%)	21 (8.3)	11 (7.6)	10 (9.3)	.406
≥2 VH positivecriteria, n (%)	70 (27.8)	36 (25)	34 (31.5)	.160
≥1 VH positivecriteria, n (%)	153 (60.7)	85 (59)	68 (63)	.308

**Legend**: CCSVI – chronic cerebrospinal venous insufficiency; VH - venous hemodynamic criteria.

Subjects who fulfilled exactly one of the 4 VH criteria and were not assessed on one VH criterion were classified originally as CCSVI borderline [11]. In the present study, 21 (8.3%) fell into this category. These individuals were conservatively categorized as CCSVI negative in the statistical analyses, potentially biasing associations toward the null.

CCSVI frequency differences between males and females were tested using the chi-square test.

**Figure 1:**
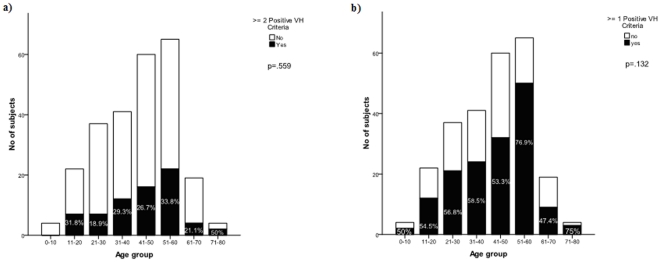
Prevalence of CCSVI diagnosis (a) or more than one venous hemodynamic criterion (b) in study subjects, according to the age group.

There was no significant difference for CCSVI diagnosis and/or presence of one or more positive VH criteria between males and females. No difference was found for presence of CCSVI or one or more positive VH criteria between different age groups (Figures 1a and b). The prevalence of CCSVI was similar with respect to source of recruitment.

### Risk/protective factors

Table 3 shows risk and protective factors that were significantly associated with the CCSVI diagnosis in logistic regression analysis. Subjects who presented with heart disease generally (p = .001), and especially heart murmurs (p = .007), a history of infectious mononucleosis (p = .002) and irritable bowel syndrome (IBS) (p = 005) were significantly associated with higher frequency of CCSVI diagnosis. There was a trend for association between higher frequency of CCSVI diagnosis and use of fish oil (p = .015) or current/previous smoking (p = .029). However, subanalysis showed that 57 of the 71 subjects who used fish oil, also had heart disease and were smoking. There was a trend for lower frequency of CCSVI in subjects who were using dietary supplements for three months or more (p = .018) or those who were using ginkgo (p = .027).

**Table 3:** Risk factors significantly associated with CCSVI in the logistic regression analysis.

Risk factors	Subjects (n = 252) (totR/totA/CCSVI)	OR	95% CI	R	p
Heart disease	227/80/32	2.7	1.5–4.96	.99	.001**
Heart murmurs	227/13/8	4.9	1.5–15.5	1.5	.007**
Infective mononucleosis	230/34/18	2.7	1.4–5.4	1.0	.002**
Irritable bowel syndrome	234/19/11	3.9	1.5–10.2	1.4	.005**
Fish oil	131/72/21	2.3	1.1–4.8	.853	.015*
Smoking	218/146/50	1.98	1.0–3.8	.683	.029*

Protective factors	Subjects (n = 252) (totR/totA/no CCSVI)	OR	95% CI	R	p
Dietary supplements	232/144/49	0.5	0.27–0.93	−.7	.018*
Gingko	32/10/8	0.1	0.02–0.8	−1.9	.027*

**Legend**: totR - total respondents on the environmental questionnaire; totA - total affected for certain condition; CCSVI - subjects presenting with CCSVI; no CCSVI - subjects not presenting with CCSVI; OR - odds ratio; CI - confidence interval; R - regression coefficient.

*represents a trend for significance (p<0.05);

**represents significance (p<0.01).

Familial connection to MS, presence of rheumatoid disorders, psoriasis, diabetes type 1, asthma, allergy, cancer, migraine, COPD, obesity, hypertension, multiple pregnancies, occupation requiring standing, use of alcohol or presence of other risk factors were not associated with increased risk for CCSVI.

## Discussion

This is the first study to investigate the association of demographic, clinical and environmental risk factors and presence of CCSVI in a large control group without known central nervous system pathology. We found that heart disease (especially heart murmurs), history of infectious mononucleosis, and IBS were significantly associated with presence of CCSVI. There was also a trend for higher prevalence of CCSVI in subjects who smoked or consumed fish oil. We also found that use of dietary supplements in general, as well as ginkgo by itself, were protective factors for this vascular condition.

Debate is still ongoing about CCSVI and its relationship with MS pathogenesis, because none of the confirmatory studies [8]–[13], [18] reproduced the originally reported results [7]. One of the possible reasons for such inconsistent results is related to the lack of standardized diagnostic guidelines related to the evaluation of the intra- and extra-cranial cerebrospinal venous system and the definition of pathology related to those anomalies. So far, it has not been possible to determine whether venous abnormalities are related to MS or are a consequence of MS. The higher prevalence of CCSVI in progressive MS patients may suggest that CCSVI may play a contributory role or be a consequence of disease progression [11].

Furthermore, there is still a lack of information about the prevalence of CCSVI in patients with other neurological and non-neurological diseases of autoimmune or other origins. In a recently published study, it was reported that prevalence of CCSVI was higher in patients with other neurologic diseases than in healthy individuals [11]. In that study, 42.3% of the patients with other neurologic diseases presented with CCSVI. However, the composition of the other neurologic disease group was biased towards immune mediated or inflammatory diseases. Therefore at this time, it cannot be excluded that prevalence of CCSVI may be increased in patients presenting with autoimmune neurologic and non-neurologic diseases in general. Further studies need to determine whether CCSVI is an MS-specific condition or can be found with high prevalence in neurologic and non-neurologic pathologic entities.

Because several recently published studies detected a substantial amount of vein abnormalities in the general population [11], [13], [18] ranging from 22% to 40%, we aimed to investigate which risk factors could be associated with the presence of CCSVI. In the present study, 27.8% of the subjects presented with CCSVI.

It is well known that established risk factors for periphery vein disorders include older age, family history, female gender, multiple pregnancies, an occupation requiring standing, and obesity in females [22]. In our study we did not find an association between these risk factors and CCSVI. In addition, there was no correlation between different age groups and CCSVI diagnosis, although increased prevalence of CCSVI and one or more VH criteria were found in subjects over 70 years of age (Figure 1). This is in line with a recent study that investigated IJV hemodynamics and the frequency of jugular venous reflux in a large cohort of elderly subjects, and reported increased prevalence of jugular venous reflux, dilated vessel lumen and slowed flow velocity in the left IJV, as well as decreased time-averaged mean velocity of bilateral IJV, in those over 70 years of age [19]. It has also been found that the prevalence of peripheral chronic venous insufficiency increases with aging [22].

One of the most interesting findings of the present study was that subjects who had a history of infectious mononucleosis showed higher frequency of CCSVI diagnosis. It is known that the Epstein-Barr virus (EBV) infection has been associated with numerous cancers. There is also considerable evidence that EBV infection is the most important risk factor candidate for the development of MS [23]. MS risk is low in individuals who are EBV negative, but increases several fold following EBV infection. There is some evidence suggesting that EBV infection can act as a precipitating factor for venous thrombo-embolism in immuno-compromised patients [24]. The mechanisms by which EBV infection might trigger thrombosis are not fully understood, but include transient elevations of anti-phospholipid antibodies [25], and EBV-induced oxidative endothelial cell injury [26]. The EBV virus was also found to have caused venous thrombosis in a patient with hereditary thrombophilia [24]. Persistent EBV infection in the small vein vessel wall can directly promote a pro-inflammatory, pro-coagulant, and pro-atherogenic environment [24]. Whether EBV infection may damage the venous endothelium, causing venous thromboses and strictures in the cranial and spinal venous drainage system, is unknown at this time. It could also be hypothesized that an EBV-mediated autoimmune attack of the intra- and extra-cranial veins can cause chronic inflammation and scarring [27]. Further longitudinal studies should investigate the relationship between CCSVI and EBV infection.

We found a significant association between CCSVI diagnosis and the presence of IBS. Previous studies have suggested that patients with IBS have increased risk of venous thrombo-embolism [28]. Serum markers associated with inflammation have also been found in patients with IBS [29]. Patients with MS and their first-degree relatives seem to be at an increased risk of acquiring certain other autoimmune diseases [30]. The risk of developing MS was 1.7 times higher for individuals who had IBS compared with those who did not [31].

The current study also identified an association between CCSVI and the presence of heart disease and, in particular, the presence of heart murmurs. Patients with cardiovascular events are at short-term increased risk of venous thrombo-embolism [32]. There is increasing evidence that arterial and venous thrombosis share several cardiovascular risk factors [33]. The pathogenesis of both disorders includes endothelial injury, platelet activation, elevated levels of intrinsic clotting factors and inflammatory markers, increased fibrinogen, and impaired fibrinolysis [34]. MS patients may have a higher risk for cardiovascular disease than the general population [35]. It has been reported that vascular co-morbidity is associated with more rapid disability progression [36] and higher risk of death [37] in patients with MS. However, as our original environmental questionnaire was biased toward risk factors for MS, we did not collect general risk factors in relatives of the healthy individual (except high blood pressure). No relationship between CCSVI and high blood pressure was found in the present study.

There was a trend in the present study for higher prevalence of CCSVI in subjects who were or currently are smokers. It has been shown that cigarette smoking is one of the most important risk factors for vascular disease in women [38]. The mechanisms responsible for the harmful effects of tobacco on the venous system have still not been elucidated. However, cigarette smoking is known to be a major factor of hypoxia through fixation of carbon monoxide and nitric oxide to hemoglobin [39]. Also, it damages the vascular endothelium, promotes vascular thrombosis, increases the relative risk of venous thrombo-embolism and is significantly associated with lower limb venous insufficiency [40]. Cigarette smoking is one of the most compelling risk factors associated with increased risk of developing [41] and progression [42] of MS.

A trend for relationship between higher prevalence of CCSVI and use of fish oil was identified in the current study. This finding has to be interpreted with caution, as only 52% of participants provided an answer to this question and 80.3% of those who used fish oil also presented heart disease and were smoking. Therefore, these preliminary findings need further investigation, but could indicate that use of fish oil showed a trend for higher CCSVI frequency, because it was used in subjects who already had other risk factors for CCSVI. Marine fish consumption is known to reduce mortality from ischemic heart disease [43]. The use of fish oil as a dietary supplement, however, is not universally recommended. In large doses, fish oil reduces plasma cholesterol and triacylglycerol, but increases low-density lipoprotein levels and the potential for free radical generation and bleeding [44]. In animal models of arterial thrombosis, fish-oil-enriched diets have been shown to have an antithrombotic effect, if the fish-oil consumption is associated with a reduction of the saturated fat intake [45].

Between 10% to 20% of people with MS have a relative with the disease, suggesting a genetic link [46]. The CTEVD study was designed to test whether MS relatives more frequently present with CCSVI diagnosis, but no relationship was found in a previous [11] or this study.

Among protective factors, we found that everyday use of dietary supplements showed a trend for lower frequency of CCSVI. Dietary supplements are widely used for improving the integrity and function of veins in the lower limbs, in order to increase the integrity of the tissue to improve the vein's function [47]. They also decrease the capillary's permeability by reducing the number and size of pores in the capillary's walls, improve the tone of or dilate the blood vessels and affect blood-clotting mechanisms [47]. In particular, we found that use of ginkgo showed a trend for a protective role in relation to CCSVI; however, because only 13% of participants answered this question, the data need to be confirmed in further studies. Ginkgo may reduce the risk of atherosclerosis by interfering with the receptor of the platelet activating factor in human cells [48]. Ginkgo also increases blood circulation to the brain, arms, and legs [49]. Ginkgo biloba is able to prevent the activation of endothelial cells, which play a key role in regulating vascular homeostasis, by hypoxic conditions both in a cell culture and in a complete perfused saphenous vein [50].

One of the limitations of our study is the small number of subjects representing races other than Caucasian. Also, there was a small number of participants who used dietary supplements such as gingko and fish oil as well as a low number of respondents for some questions; these could have influenced the results about risk and protective factors for CCSVI in relation to those variables. Another limit could be related to our decision to select controls from different sources of recruitment. Spouses or relatives of MS patients may have shared environmental risk factors with MS patients; however, no CCSVI prevalence differences were found according to the source of recruitment. Further studies enrolling a larger cohort of controls are needed to determine the role of risk factors for CCSVI.

In conclusion, our study showed that risk factors for CCSVI differ from established risk factors for peripheral venous diseases.

## Supporting Information

Appendix S1 **Environmental Factors in Multiple Sclerosis (questionnaire).**
(DOC)

Appendix S2 **Environmental Factors in Multiple Sclerosis.**
(DOC)
