# Supplementary material for: Risk Factors for Chronic Cerebrospinal Venous Insufficiency (CCSVI) in a Large Cohort of Volunteers
Source: PLoS One. 2011 Nov 30;6(11):e28062. doi: 10.1371/journal.pone.0028062 (PMC3227626; doi:10.1371/journal.pone.0028062)
Supplement: Appendix S1 — Environmental Factors in Multiple Sclerosis (questionnaire). (DOC) [file pone.0028062.s001.doc]

**Appendix S1: Environmental Factors in Multiple Sclerosis (questionnaire)**

**1) Informed Consent**

🔾 Yes, Informed Consent

🔾 Yes, Informed Assent and Parental Consent

**2) Date of Informed Consent**

____________________________________________________________

**3) Study Identifier**

____________________________________________________________

**4) Subject Identifier**

____________________________________________________________

**5) Survey Recorder**

Last name ____________________________________________________________

Firstname____________________________________________________________

**6) Survey Date**

____________________________________________________________

**7) Reason for Declining Survey**

🔾 Not applicable, Patient agreed

🔾 Declined, too time consuming

🔾 Declined, did not want to share personal information

🔾 Declined, other

🔾 Excluded, cognitive impairment

🔾 Excluded, disability

🔾 Excluded, other

🔾 Other (please specify)

If you selected other, please specify ______________________________________________________________________

**8) Subject's Date of Birth**

____________________________________________________________

**9) Subject Primary Classification**
🔾 Adult MS Patient 🔾 Grandparent of MS Patient

🔾 Pediatric (0 through 17 year old) MS Patient 🔾 Other Relative of MS Patient

🔾 Spouse of MS Patient 🔾 Other Neurological Disease Control

🔾 Parent of MS Patient 🔾 Other Autoimmune Disease Control

🔾 Sibling of MS Patient 🔾 Healthy Control

🔾 Child of MS Patient 🔾 Other

If you selected other, please specify

____________________________________________________________

**10) Gender**

🔾 Male 🔾 Transgender Male

🔾 Female 🔾 Transgender Female

**11) Dominant hand**

🔾 Left 🔾 Right 🔾 Ambidextrous

**12) Height and Weight**

Height in inches______________________

Weight in Pounds_____________________

**13) What is your body shape?**

🔾 V-shape: have proportionally smaller buttocks, bigger chests and wider shoulders, which makes for a V-shape of the torso.

🔾 Hourglass shape: significantly narrower in the waist both in front view and profile view. The waist is narrower than the chest region due to the breasts, and narrower than the hip region due to the width of the buttocks.

🔾 Apple shape: the stomach region is wider than the hip section.

🔾 Pear, spoon, or bell: The hip section is wider than the upper body.

🔾 Rectangle, straight, or banana: The hip, waist, and shoulder sections are relatively similar.

🔾 Don't know.

🔾 Declined to answer.

**14) Where do you tend to carry extra weight?**

🔾 Back, chest, arms and stomach 🔾 Generally do not carry extra weight

🔾 Both upper and lower body 🔾 Not applicable, e.g., child

🔾 Hips, thighs and buttocks 🔾 Do not know

🔾 Stomach and buttocks 🔾 Declined to answer

**15) Are you a twin?**

🔾 Not a twin or multiple birth 🔾 Identical twin

🔾 Fraternal twin 🔾 Multiple births

**16) Gender of your twin sibling**

🔾 Male 🔾 Female 🔾 Multiple births

**17) Information on Siblings**Number of Full Siblings (Do not include yourself.)____________

Number of Full brothers (Do not include yourself if male.)________

Birth Order among Full Siblings_____________________________

Number of Half-Siblings___________________________________

Number of Half-Brothers__________________________________

Number of Step-Siblings__________________________________

Number of Step Brothers_________________________________

Number of Adopted Siblings________________________________

Number of Adopted Brothers_______________________________

**18) Do you have any relatives with MS?**

🔾 Yes 🔾 No 🔾 Don't know

**19) Which of your immediate family or siblings has multiple sclerosis?**

|  | Yes | No | Don't Know | Not Applicable |
| --- | --- | --- | --- | --- |
| Mother | 🔾 | 🔾 | 🔾 | 🔾 |
| Father | 🔾 | 🔾 | 🔾 | 🔾 |
| Son | 🔾 | 🔾 | 🔾 | 🔾 |
| Daughter | 🔾 | 🔾 | 🔾 | 🔾 |
| Twin Brother | 🔾 | 🔾 | 🔾 | 🔾 |
| Twin Sister | 🔾 | 🔾 | 🔾 | 🔾 |
| Brother | 🔾 | 🔾 | 🔾 | 🔾 |
| Sister | 🔾 | 🔾 | 🔾 | 🔾 |
| Half Brother | 🔾 | 🔾 | 🔾 | 🔾 |
| Half Sister | 🔾 | 🔾 | 🔾 | 🔾 |
| Step-Brother | 🔾 | 🔾 | 🔾 | 🔾 |
| Step-Sister | 🔾 | 🔾 | 🔾 | 🔾 |
| Adopted Brother | 🔾 | 🔾 | 🔾 | 🔾 |
| Adopted Sister | 🔾 | 🔾 | 🔾 | 🔾 |
| Other | 🔾 | 🔾 | 🔾 | 🔾 |

**20) Which of your blood relatives has multiple sclerosis?**

|  | Yes | No | Don't Know | Not Applicable |
| --- | --- | --- | --- | --- |
| Paternal Grandfather | 🔾 | 🔾 | 🔾 | 🔾 |
| Paternal Grandmother | 🔾 | 🔾 | 🔾 | 🔾 |
| Maternal Grandfather | 🔾 | 🔾 | 🔾 | 🔾 |
| Maternal Grandmother | 🔾 | 🔾 | 🔾 | 🔾 |
| Paternal Uncle | 🔾 | 🔾 | 🔾 | 🔾 |
| Paternal Aunt | 🔾 | 🔾 | 🔾 | 🔾 |
| Maternal Uncle | 🔾 | 🔾 | 🔾 | 🔾 |
| Maternal Aunt | 🔾 | 🔾 | 🔾 | 🔾 |
| Paternal Cousin | 🔾 | 🔾 | 🔾 | 🔾 |
| Maternal Cousin | 🔾 | 🔾 | 🔾 | 🔾 |
| Other | 🔾 | 🔾 | 🔾 | 🔾 |

**21) Pregnancies and Live Births**

🔾 None 🔾 Not applicable, C

🔾 Not applicable, Male 🔾 Yes

**22) Total number of pregnancies** _____________________

**23) Total number of live births** _______________________

**24) Number of pregnancies BEFORE onset of MS** _______________

**25) Number of Live Births BEFORE onset of MS** _________________

**26) Biological, Step and Adopted Children**

Number of Biological Children ____________________________

Sex and Years of Birth of Biological Children (Use M/F Year of Birth followed by commas)

_________________________________________________________

Number of Step-Children ______________________________

Sex and Years your Step children Joined your household (Use M/F Year of Joining followed by commas)

____________________________________________

Number of Adopted Children ______________________________

Sex and Years of Adoption of Adopted Children (Use M/F Year of joining followed by commas)

_________________________________________________________

**27) Did you have any of the following conditions during pregnancy?**

🔾 Gestational diabetes or poorly controlled high blood sugar

🔾 Pregnancy induced hypertension

🔾 Infection with rubella

🔾 Alcohol intake

🔾 Exposure to retinoic acid ( acne treatment medication)

🔾 Other complication

- If yes, please specify ______________________________________________

**28) Did you have any complication during labor?**

🔾 Preterm labor 🔾 Forceps delivery

🔾 Prolonged labor 🔾 Cesarean section

🔾 Obstructive labor

**These items are to be answered by the mother / father of the MS child if present**

**29) Did you have any of the following conditions during pregnancy of your MS child?**

🔾 Gestational diabetes or poorly controlled high blood sugar

🔾 Pregnancy induced hypertension

🔾 Infection with rubella

🔾 Alcohol intake

🔾 Exposure to retinoic acid ( acne treatment medication)

🔾 Other complication

If yes, please specify _____________________________

**30)** **Did you have any complication during labor?**

Please specify ____________________________________________________

**31) race (US Census Bureau)**
🔾 White 🔾 American Indian/Alaska Native

🔾 Hispanic/Latino 🔾 Other

🔾 Black/African-American 🔾 Declined

🔾 Asian 🔾 Other (please specify)___________________

**32) Your Biological Father's Place of Birth**

City___________________________ State/Province______________________ Country_________________

**33) Your Biological Mother's of Birth**

City___________________________ State/Province______________________ Country_________________

**34) Your Paternal Grandfather’s Place of Birth**

City___________________________ State/Province______________________ Country_________________

**35) Your Paternal Grandmothers’ Place of Birth**

City___________________________ State/Province______________________ Country_________________

**36) Your Mater**n**al Grandfather’s Place of Birth**

City___________________________ State/Province______________________ Country_________________

**37) Your Maternal Grandmothers’ Place of Birth**

City___________________________ State/Province______________________ Country_________________

**38) Ethnic Background. Please carefully examine and choose from the list of Ethnic Group Categories. If you DO NOT know the ethnicity of a family member, please select Unknown. Select two categories at most.**

- USA or Canada: White not Hispanic, NOT French-Canadian
- African
- African-American
- Western European: White not Hispanic (Germany, France, Netherlands, UK, Switzerland, Ireland, Austria, Belgium)
- Mediterranean: White not Hispanic (Italy, Greece, Cyprus, Turkey)
- Scandinavian: White not Hispanic (Norway, Sweden, Denmark NOT Finland)
- Russian: White not Hispanic
- Polish, Slavic, Eastern European countries: White not Hispanic
- White, NOT Hispanic, Other
- USA or Canada: White AND Hispanic origin
- Mexico, Central America: White and Hispanic
- Caribbean, Puerto Rico, Cuba, Dominican Republic, Jamaica, Haiti: Hispanic (Specify)
- Native American (Specify Tribe)
- Japan: Asian/Pacific Rim
- China: Asian/Pacific Rim
- Indian Sub-continent: Asian/Pacific (includes India, Bangladesh, Nepal, Sri Lanka, Pakistan)
- Other Asian (Specify)
- Amish: Special population
- Arab Middle Eastern: Special population (includes Saudi Arabia, Lebanon, Yemen, Iraq etc.)
- Arab North African: Special population (includes Egypt, Algeria, Morocco etc.)
- Non-Askenazi Jew: Special population
- Pennsylvania Dutch: Special population
- Finnish: Special population
- Icelandic: Special population
- French Canadian: Special population
- Inuit, Alaska Native: Special population
- Other (Specify)
- Unknown

|  | FATHER | MOTHER |
| --- | --- | --- |
| Your primary Ethnicity |  |  |
| Father's primary ethnicity |  |  |
| Mother's primary ethnicity |  |  |
| Paternal Grandfather |  |  |
| Paternal Grandmother |  |  |
| Maternal Grandfather |  |  |
| Maternal Grandmothers |  |  |

**39) Educational History**

|  | None | 1 year | 2 years | 3 years | 4 years | 5 or more years |
| --- | --- | --- | --- | --- | --- | --- |
| Elementary School | 🔾 | 🔾 | 🔾 | 🔾 | 🔾 | 🔾 |
| Middle School | 🔾 | 🔾 | 🔾 | 🔾 | 🔾 | 🔾 |
| High School | 🔾 | 🔾 | 🔾 | 🔾 | 🔾 | 🔾 |
| Two-Year College | 🔾 | 🔾 | 🔾 | 🔾 | 🔾 | 🔾 |
| University/Four-Year College | 🔾 | 🔾 | 🔾 | 🔾 | 🔾 | 🔾 |
| Trade School | 🔾 | 🔾 | 🔾 | 🔾 | 🔾 | 🔾 |
| Professional School (e.g., MD, MPH, DDS, JD, PharmD) | 🔾 | 🔾 | 🔾 | 🔾 | 🔾 | 🔾 |
| Masters Degree (e.g., MA, MS, MBA) | 🔾 | 🔾 | 🔾 | 🔾 | 🔾 | 🔾 |
| Doctoral Degree | 🔾 | 🔾 | 🔾 | 🔾 | 🔾 | 🔾 |
| Post doctoral, residency, fellowship | 🔾 | 🔾 | 🔾 | 🔾 | 🔾 | 🔾 |

**40) Employment Status**

| - Employed: Full time - Employed: Part time - Employed: Reduced due to disability - Employed: Adapted work due to disability - Employed at home - Homemaker - Student - Volunteer - Workers compensation - Unemployed– not looking for work |
| --- |

**41) Domestic status**

|  | One Year or Less | 1+ to 2 years | 3 to 5 years | 6 to 10 years | 11 to 15 years | 16 to 20 years | More than 20 years |
| --- | --- | --- | --- | --- | --- | --- | --- |
| Single |  |  |  |  |  |  |  |
| Married |  |  |  |  |  |  |  |
| Cohabiting |  |  |  |  |  |  |  |
| Divorced/ Separated |  |  |  |  |  |  |  |
| Widowed |  |  |  |  |  |  |  |
| Never married |  |  |  |  |  |  |  |
| none of the above |  |  |  |  |  |  |  |
| Not Applicable, Minor |  |  |  |  |  |  |  |
| Not Applicable, Other |  |  |  |  |  |  |  |

**42) Living Status**

|  | One Year or Less | 1+ to 2 years | 3 to 5 years | 6 to 10 years | 11 to 15 years | 16 to 20 years | More than 20 years | Don’t remember |
| --- | --- | --- | --- | --- | --- | --- | --- | --- |
| Living with Spouse/partner |  |  |  |  |  |  |  |  |
| Living with Parent |  |  |  |  |  |  |  |  |
| Living with Children |  |  |  |  |  |  |  |  |
| Living with Sibling |  |  |  |  |  |  |  |  |
| Living with Other Relative |  |  |  |  |  |  |  |  |
| Living with Friend |  |  |  |  |  |  |  |  |
| Living with health-related companion |  |  |  |  |  |  |  |  |
| Living with domestic help |  |  |  |  |  |  |  |  |
| Living in nursing home or ALC |  |  |  |  |  |  |  |  |
| Homeless |  |  |  |  |  |  |  |  |
| Other |  |  |  |  |  |  |  |  |
| Not Applicable |  |  |  |  |  |  |  |  |
| Declined |  |  |  |  |  |  |  |  |

**43) Residential History. Residence at Birth**

| Street of Birth_____________________ |
| --- |
| City of Birth_____________________ |
| State of Birth_____________________ |
| Zip Code of Birth_____________________ |
| Country of Birth (If not USA) ______________________________________ |

**44) Residential History. Current Residence.**

Start Year at Residence_______________________________

Moved Before MS Onset? _____ Yes/No Type_____________

Moved Before 15-years of Age? Yes/No Type_________________

Street__________________________________________________

City___________________________________________________

State______________________________________

Zip Code__________________________________

Country (If not USA)_______________________________

**45) Residential History. Residence 1. Start from the earliest residence. Consider college dormitories, overseas jobs, military or peace corps assignments and other transitional housing as a residence also. Provide as much information as you can remember.**

Start Year at Residence_______________________________

End Year at Residence_________________________________________

Moved Before MS Onset? _____ Yes/No Type_____________

Moved Before 15-years of Age? Yes/No Type_________________

Street__________________________________________________

City___________________________________________________

State______________________________________

Zip Code__________________________________

Country (If not USA)_______________________________

**46) Residential History. Residence 2. Start from the earliest residence. Consider college dormitories, overseas jobs, military or Peace Corps assignments and other transitional housing as a residence also. Provide as much information as you can remember.**

Start Year at Residence_______________________________

End Year at Residence_________________________________________

Moved Before MS Onset? _____ Yes/No Type_____________

Moved Before 15-years of Age? Yes/No Type_________________

Street__________________________________________________

City___________________________________________________

State______________________________________

Zip Code__________________________________

Country (If not USA)_______________________________

**47) Residential History. Residence 3. Start from the earliest residence. Consider college dormitories, overseas jobs, military or peace corps assignments and other transitional housing as a residence also. Provide as much information as you can remember.**

Start Year at Residence_______________________________

End Year at Residence_________________________________________

Moved Before MS Onset? _____ Yes/No Type_____________

Moved Before 15-years of Age? Yes/No Type_________________

Street__________________________________________________

City___________________________________________________

State______________________________________

Zip Code__________________________________

Country (If not USA)_______________________________

**48) Residential History. Residence 4. Start from the earliest residence. Consider college dormitories, overseas jobs, military or peace corps assignments and other transitional housing as a residence also. Provide as much information as you can remember.**

Start Year at Residence_______________________________

End Year at Residence_________________________________________

Moved Before MS Onset? _____ Yes/No Type_____________

Moved Before 15-years of Age? Yes/No Type_________________

Street__________________________________________________

City___________________________________________________

State______________________________________

Zip Code__________________________________

Country (If not USA)_______________________________

**49) Residential History. Residence 5. Start from the earliest residence. Consider college dormitories, overseas jobs, military or peace corps assignments and other transitional housing as a residence also. Provide as much information as you can remember.**

Start Year at Residence_______________________________

End Year at Residence_________________________________________

Moved Before MS Onset? _____ Yes/No Type_____________

Moved Before 15-years of Age? Yes/No Type_________________

Street__________________________________________________

City___________________________________________________

State______________________________________

Zip Code__________________________________

Country (If not USA)_______________________________

**50) Residential History. Residence 6. Start from the earliest residence. Consider college dormitories, overseas jobs, military or peace corps assignments and other transitional housing as a residence also. Provide as much information as you can remember.**

Start Year at Residence_______________________________

End Year at Residence_________________________________________

Moved Before MS Onset? _____ Yes/No Type_____________

Moved Before 15-years of Age? Yes/No Type_________________

Street__________________________________________________

City___________________________________________________

State______________________________________

Zip Code__________________________________

Country (If not USA)_______________________________

**51) Residential History. Residence 7. Start from the earliest residence. Consider college dormitories, overseas jobs, military or peace corps assignments and other transitional housing as a residence also. Provide as much information as you can remember.**

Start Year at Residence_______________________________

End Year at Residence_________________________________________

Moved Before MS Onset? _____ Yes/No Type_____________

Moved Before 15-years of Age? Yes/No Type_________________

Street__________________________________________________

City___________________________________________________

State______________________________________

Zip Code__________________________________

Country (If not USA)_______________________________

**52) Residential History. Residence 8. Start from the earliest residence. Consider college dormitories, overseas jobs, military or Peace Corps assignments and other transitional housing as a residence also. Provide as much information as you can remember.**

Start Year at Residence_______________________________

End Year at Residence_________________________________________

Moved Before MS Onset? _____ Yes/No Type_____________

Moved Before 15-years of Age? Yes/No Type_________________

Street__________________________________________________

City___________________________________________________

State______________________________________

Zip Code__________________________________

Country (If not USA)_______________________________

**53) Have you ever lived in a Foreign Country for an uninterrupted period of 3 or Months. Include college stays, overseas jobs, military or Peace Corps assignments as foreign residence also. Provide as much information as you can.  Consider Canada and Mexico as foreign countries as well.**

🔾 Yes 🔾 Declined

🔾 No 🔾 Other (please specify)______________________

**54) Foreign Residence 1. Start from the earliest residence. Provide as much information as you can remember.**

Start Year at Residence_______________________________

End Year at Residence_________________________________________

Months of Foreign Residence_________________________________

City___________________________________________________

State/Province______________________________________

Country_______________________________

**55) Foreign Residence 2. Start from the earliest residence. Provide as much information as you can remember.**

Start Year at Residence_______________________________

End Year at Residence_________________________________________

Months of Foreign Residence_________________________________

City___________________________________________________

State/Province______________________________________

Country_______________________________

**56) Foreign Residence 3. Start from the earliest residence. Provide as much information as you can remember.**

Start Year at Residence_______________________________

End Year at Residence_________________________________________

Months of Foreign Residence_________________________________

City___________________________________________________

State/Province______________________________________

Country_______________________________

**57) Foreign Residence 4. Start from the earliest residence. Provide as much information as you can remember.**

Start Year at Residence_______________________________

End Year at Residence_________________________________________

Months of Foreign Residence_________________________________

City___________________________________________________

State/Province______________________________________

Country_______________________________

**58) Foreign Residence 5. Start from the earliest residence. Provide as much information as you can remember.**

Start Year at Residence_______________________________

End Year at Residence_________________________________________

Months of Foreign Residence_________________________________

City___________________________________________________

State/Province______________________________________

Country_______________________________

**59) Employment and Occupation History. Current Job or Occupation. Provide as much information as you can remember.**

Start Year at Job_______________________________

Industry_________________________________________

Company_________________________________

Job Title_________________________________

Job Activities___________________________________________

City___________________________________________________

State______________________________________

Zip Code__________________________________

Country (If not USA)_______________________________

**60) Employment and Occupation History. Job or Occupation 1. This is the First Job or Occupation you had for 3-Months or more. Provide as much information as you can remember.**

Start Year at Job_______________________________

End Year at Job_______________________________

Industry_________________________________________

Company_________________________________

Job Title_________________________________

Job Activities___________________________________________

City___________________________________________________

State______________________________________

Zip Code__________________________________

Country (If not USA)_______________________________

**61) Employment and Occupation History. Job or Occupation 2. Start from the first Job or Occupation you had for 3-Months or more. Provide as much information as you can remember.**

Start Year at Job_______________________________

End Year at Job_______________________________

Industry_________________________________________

Company_________________________________

Job Title_________________________________

Job Activities___________________________________________

City___________________________________________________

State______________________________________

Zip Code__________________________________

Country (If not USA)_______________________________

**62) Employment and Occupation History. Job or Occupation 3. Provide as much information as you can remember.**

Start Year at Job_______________________________

End Year at Job_______________________________

Industry_________________________________________

Company_________________________________

Job Title_________________________________

Job Activities___________________________________________

City___________________________________________________

State______________________________________

Zip Code__________________________________

Country (If not USA)_______________________________

**63) Employment and Occupation History. Job or Occupation 4. Provide as much information as you can remember.**

Start Year at Job_______________________________

End Year at Job_______________________________

Industry_________________________________________

Company_________________________________

Job Title_________________________________

Job Activities___________________________________________

City___________________________________________________

State______________________________________

Zip Code__________________________________

Country (If not USA)_______________________________

**64) Employment and Occupation History. Job or Occupation 5. Provide as much information as you can remember.**

Start Year at Job_______________________________

End Year at Job_______________________________

Industry_________________________________________

Company_________________________________

Job Title_________________________________

Job Activities___________________________________________

City___________________________________________________

State______________________________________

Zip Code__________________________________

Country (If not USA)_______________________________

**65) Employment and Occupation History. Job or Occupation 6. Provide as much information as you can remember.**

Start Year at Job_______________________________

End Year at Job_______________________________

Industry_________________________________________

Company_________________________________

Job Title_________________________________

Job Activities___________________________________________

City___________________________________________________

State______________________________________

Zip Code__________________________________

Country (If not USA)_______________________________

**66) Employment and Occupation History. Job or Occupation 7. Provide as much information as you can remember.**

Start Year at Job_______________________________

End Year at Job_______________________________

Industry_________________________________________

Company_________________________________

Job Title_________________________________

Job Activities___________________________________________

City___________________________________________________

State______________________________________

Zip Code__________________________________

Country (If not USA)_______________________________

**67) Employment and Occupation History. Job or Occupation 8. Provide as much information as you can remember.**

Start Year at Job_______________________________

End Year at Job_______________________________

Industry_________________________________________

Company_________________________________

Job Title_________________________________

Job Activities___________________________________________

City___________________________________________________

State______________________________________

Zip Code__________________________________

Country (If not USA)_______________________________

**68) Sleep. Indicate the average number of hours of sleep you get at Night**

Non-MS Subject or Before Onset of MS

🔾 2 hours or Less 🔾 9 to 10 hours

🔾 3 to 4 hours 🔾 10 to 12 hours

🔾 5 to 6 hours 🔾 More than 12 hours

🔾 6 to 7 hours 🔾 Declined

🔾 8 hours

After Onset of MS

🔾 2 hours or Less 🔾 9 to 10 hours

🔾 3 to 4 hours 🔾 10 to 12 hours

🔾 5 to 6 hours 🔾 More than 12 hours

🔾 6 to 7 hours 🔾 Declined

🔾 8 hours

Last 3-Months?

🔾 2 hours or Less 🔾 9 to 10 hours

🔾 3 to 4 hours 🔾 10 to 12 hours

🔾 5 to 6 hours 🔾 More than 12 hours

🔾 6 to 7 hours 🔾 Declined

🔾 8 hours

**69) Sleep. Indicate whether you sleep or take daytime naps?**

Non-MS Subject or Before Onset of MS

🔾 Do not Sleep or Nap in Daytime 🔾 Nap more than 3 times

🔾 Nap Once for 30 minutes 🔾 Sleep for 3 hours or more in the Daytime

🔾 Nap Once for 1 hour or more 🔾 Don't Remember

🔾 Nap 2 times 🔾 Declined

🔾 Nap 3 times

After Onset of MS

🔾 Do not Sleep or Nap in Daytime 🔾 Nap more than 3 times

🔾 Nap Once for 30 minutes 🔾 Sleep for 3 hours or more in the Daytime

🔾 Nap Once for 1 hour or more 🔾 Don't Remember

🔾 Nap 2 times 🔾 Declined

🔾 Nap 3 times

Last 3-Months

🔾 Do not Sleep or Nap in Daytime 🔾 Nap more than 3 times

🔾 Nap Once for 30 minutes 🔾 Sleep for 3 hours or more in the Daytime

🔾 Nap Once for 1 hour or more 🔾 Don't Remember

🔾 Nap 2 times 🔾 Declined

🔾 Nap 3 times

**70) Sleep. Indicate the side you sleep on, e.g., left, right, back, face down, etc.**
Non-MS Subject or Before Onset of MS

🔾 Mostly on Back 🔾 Mostly on Right

🔾 Mostly on Stomach 🔾 Mostly on Left

🔾 Evenly on Back, Left and Right 🔾 Sleep Sitting

🔾 Evenly on Stomach, Left and Right 🔾 Don't know

🔾 Evenly on Right and Left 🔾 Declined

After Onset of MS

🔾 Mostly on Back 🔾 Mostly on Right

🔾 Mostly on Stomach 🔾 Mostly on Left

🔾 Evenly on Back, Left and Right 🔾 Sleep Sitting

🔾 Evenly on Stomach, Left and Right 🔾 Don't know

🔾 Evenly on Right and Left 🔾 Declined

Last 3-Months

🔾 Mostly on Back 🔾 Mostly on Right

🔾 Mostly on Stomach 🔾 Mostly on Left

🔾 Evenly on Back, Left and Right 🔾 Sleep Sitting

🔾 Evenly on Stomach, Left and Right 🔾 Don't know

🔾 Evenly on Right and Left 🔾 Declined

**71) Sleep. Indicate the average Number of Pillows.**

Non-MS Subject or Before Onset of MS

Number of Pillows

🔾 1 pillow 🔾 More than 4 Pillows

🔾 2 Pillows 🔾 Declined

🔾 3 Pillows 🔾 No Pillow

🔾 4 Pillows

After Onset of MS

Number of Pillows

🔾 1 pillow 🔾 More than 4 Pillows

🔾 2 Pillows 🔾 Declined

🔾 3 Pillows 🔾 No Pillow

🔾 4 Pillows

Last 3-Months?

Number of Pillows

🔾 1 pillow 🔾 More than 4 Pillows

🔾 2 Pillows 🔾 Declined

🔾 3 Pillows 🔾 No Pillow

🔾 4 Pillows

**72) Sleep. Do you use Extra Pillows for Lower legs, Upper legs, Lower back or Arms?**

Non-MS Subject or Before Onset of MS

🔾 No Extra Pillows 🔾 Pillow at Lower Back

🔾 Pillow for Lower legs 🔾 Pillow at Side, Arms/Shoulders

🔾 Pillow at Knees 🔾 Stuffed Animal

🔾 Pillow at Upper Legs 🔾 Declined

After Onset of MS

🔾 No Extra Pillows 🔾 Pillow at Lower Back

🔾 Pillow for Lower legs 🔾 Pillow at Side, Arms/Shoulders

🔾 Pillow at Knees 🔾 Stuffed Animal

🔾 Pillow at Upper Legs 🔾 Declined

Last 3-Months?

🔾 No Extra Pillows 🔾 Pillow at Lower Back

🔾 Pillow for Lower legs 🔾 Pillow at Side, Arms/Shoulders

🔾 Pillow at Knees 🔾 Stuffed Animal

🔾 Pillow at Upper Legs 🔾 Declined

**73) Sleep. Do you use Prescription or Over-the-Counter Sleep Medicine. These can include products such as Ambien, product containing dextromethorphan, etc.**

Non-MS Subject or Before Onset of MS

🔾 Never 🔾 Every night

🔾 Less than once a month 🔾 Don’t Remember

🔾 1-3 times a month 🔾 Not Applicable

🔾 Once a week 🔾 Not Applicable at this Stage

🔾 2-4 times a week 🔾 Other (specify)

🔾 5-6 times a week 🔾 Declined

After Onset of MS

🔾 Never 🔾 Every night

🔾 Less than once a month 🔾 Don’t Remember

🔾 1-3 times a month 🔾 Not Applicable

🔾 Once a week 🔾 Not Applicable at this Stage

🔾 2-4 times a week 🔾 Other (specify)

🔾 5-6 times a week 🔾 Declined

Last 3-Months?

🔾 Never 🔾 Every night

🔾 Less than once a month 🔾 Don’t Remember

🔾 1-3 times a month 🔾 Not Applicable

🔾 Once a week 🔾 Not Applicable at this Stage

🔾 2-4 times a week 🔾 Other (specify)

🔾 5-6 times a week 🔾 Declined

**74) Sleep. Do have you been diagnosed with sleep disorder such as sleep apnea, narcolepsy, restless leg syndrome, etc.**

Non-MS Before Onset of MS

| 🔾 Insomnia | 🔾 Delayed sleep phase syndrome | 🔾 Sleep talking (or somniloquy) |
| --- | --- | --- |
| 🔾 Narcolepsy | 🔾 REM sleep behavior disorder | 🔾 Sleep sex (or sexsomnia) |
| 🔾 Snoring - Not a disorder but can be a symptom | 🔾 Non-24-hour sleep-wake syndrome | 🔾 Exploding head syndrome - Waking up in the night hearing loud noises. |
| 🔾 Obstructive sleep apnea | 🔾 Advanced sleep phase syndrome | 🔾 Sleeping sickness |
| 🔾 Restless leg syndrome | 🔾 Sleep terror | 🔾 Other |
| 🔾 Periodic limb movement disorder | 🔾 Sleepwalking (or somnambulism) | 🔾 No known sleep disorder |
| 🔾 Recurrent hypersomnia - including Kleine-Levin syndrome | 🔾 Bruxism (Tooth-grinding) | 🔾 Have sleep problems but never saw doctor |
| 🔾 Post-traumatic hypersomnia | 🔾 Bedwetting or sleep enuresis. | 🔾 Declined |
| 🔾 Healthy hypersomnia |  |  |

After Onset of MS

| 🔾 Insomnia | 🔾 Delayed sleep phase syndrome | 🔾 Sleep talking (or somniloquy) |
| --- | --- | --- |
| 🔾 Narcolepsy | 🔾 REM sleep behavior disorder | 🔾 Sleep sex (or sexsomnia) |
| 🔾 Snoring - Not a disorder but can be a symptom | 🔾 Non-24-hour sleep-wake syndrome | 🔾 Exploding head syndrome - Waking up in the night hearing loud noises. |
| 🔾 Obstructive sleep apnea | 🔾 Advanced sleep phase syndrome | 🔾 Sleeping sickness |
| 🔾 Restless leg syndrome | 🔾 Sleep terror | 🔾 Other |
| 🔾 Periodic limb movement disorder | 🔾 Sleepwalking (or somnambulism) | 🔾 No known sleep disorder |
| 🔾 Recurrent hypersomnia - including Kleine-Levin syndrome | 🔾 Bruxism (Tooth-grinding) | 🔾 Have sleep problems but never saw doctor |
| 🔾 Post-traumatic hypersomnia | 🔾 Bedwetting or sleep enuresis. | 🔾 Declined |
| 🔾 Healthy hypersomnia |  |  |

Last 3-Months?

| 🔾 Insomnia | 🔾 Delayed sleep phase syndrome | 🔾 Sleep talking (or somniloquy) |
| --- | --- | --- |
| 🔾 Narcolepsy | 🔾 REM sleep behavior disorder | 🔾 Sleep sex (or sexsomnia) |
| 🔾 Snoring - Not a disorder but can be a symptom | 🔾 Non-24-hour sleep-wake syndrome | 🔾 Exploding head syndrome - Waking up in the night hearing loud noises. |
| 🔾 Obstructive sleep apnea | 🔾 Advanced sleep phase syndrome | 🔾 Sleeping sickness |
| 🔾 Restless leg syndrome | 🔾 Sleep terror | 🔾 Other |
| 🔾 Periodic limb movement disorder | 🔾 Sleepwalking (or somnambulism) | 🔾 No known sleep disorder |
| 🔾 Recurrent hypersomnia - including Kleine-Levin syndrome | 🔾 Bruxism (Tooth-grinding) | 🔾 Have sleep problems but never saw doctor |
| 🔾 Post-traumatic hypersomnia | 🔾 Bedwetting or sleep enuresis. | 🔾 Declined |
| 🔾 Healthy hypersomnia |  |  |

**75) Physical Activity and Exercise. Physical and Exercise is defined as 30 minutes or more of moderate intensity activity. Moderate intensity is equivalent to Brisk Walking.**

|  | Non-MS Subject or Before Onset of MS | | | | | | | | | | | | | | |
| --- | --- | --- | --- | --- | --- | --- | --- | --- | --- | --- | --- | --- | --- | --- | --- |
|  | Never | Once a Year | 2-4 Times a Year | 5-6 Times a Year | 7-11 Times a Year | Once a Month | 2-3 Times Month | Once a Week | 2-4 Times a Week | 5-6 Times a Week | Once a Day | 2 Times a Day | 3 or More Times a Day | Not Applicable at this Stage | Declined |
| Strenuous job-related walking | 🔾 | 🔾 | 🔾 | 🔾 | 🔾 | 🔾 | 🔾 | 🔾 | 🔾 | 🔾 | 🔾 | 🔾 | 🔾 | 🔾 | 🔾 |
| Strenuous job-related lifting, moving | 🔾 | 🔾 | 🔾 | 🔾 | 🔾 | 🔾 | 🔾 | 🔾 | 🔾 | 🔾 | 🔾 | 🔾 | 🔾 | 🔾 | 🔾 |
| Yoga, pilates, flexibility training | 🔾 | 🔾 | 🔾 | 🔾 | 🔾 | 🔾 | 🔾 | 🔾 | 🔾 | 🔾 | 🔾 | 🔾 | 🔾 | 🔾 | 🔾 |
| Weights, strength training | 🔾 | 🔾 | 🔾 | 🔾 | 🔾 | 🔾 | 🔾 | 🔾 | 🔾 | 🔾 | 🔾 | 🔾 | 🔾 | 🔾 | 🔾 |
| Tai Chi | 🔾 | 🔾 | 🔾 | 🔾 | 🔾 | 🔾 | 🔾 | 🔾 | 🔾 | 🔾 | 🔾 | 🔾 | 🔾 | 🔾 | 🔾 |
| Aerobics exercises | 🔾 | 🔾 | 🔾 | 🔾 | 🔾 | 🔾 | 🔾 | 🔾 | 🔾 | 🔾 | 🔾 | 🔾 | 🔾 | 🔾 | 🔾 |
| Running | 🔾 | 🔾 | 🔾 | 🔾 | 🔾 | 🔾 | 🔾 | 🔾 | 🔾 | 🔾 | 🔾 | 🔾 | 🔾 | 🔾 | 🔾 |
| Walking | 🔾 | 🔾 | 🔾 | 🔾 | 🔾 | 🔾 | 🔾 | 🔾 | 🔾 | 🔾 | 🔾 | 🔾 | 🔾 | 🔾 | 🔾 |
| Swimming | 🔾 | 🔾 | 🔾 | 🔾 | 🔾 | 🔾 | 🔾 | 🔾 | 🔾 | 🔾 | 🔾 | 🔾 | 🔾 | 🔾 | 🔾 |
| Other aerobic, Skiing, climbing, rowing | 🔾 | 🔾 | 🔾 | 🔾 | 🔾 | 🔾 | 🔾 | 🔾 | 🔾 | 🔾 | 🔾 | 🔾 | 🔾 | 🔾 | 🔾 |
| Court sports, e.g., tennis, basketball, racketball, squash | 🔾 | 🔾 | 🔾 | 🔾 | 🔾 | 🔾 | 🔾 | 🔾 | 🔾 | 🔾 | 🔾 | 🔾 | 🔾 | 🔾 | 🔾 |
| Field sport, soccer, lacrosse, field hockey | 🔾 | 🔾 | 🔾 | 🔾 | 🔾 | 🔾 | 🔾 | 🔾 | 🔾 | 🔾 | 🔾 | 🔾 | 🔾 | 🔾 | 🔾 |
| Contact field sport, rugby, football | 🔾 | 🔾 | 🔾 | 🔾 | 🔾 | 🔾 | 🔾 | 🔾 | 🔾 | 🔾 | 🔾 | 🔾 | 🔾 | 🔾 | 🔾 |
| Boxing | 🔾 | 🔾 | 🔾 | 🔾 | 🔾 | 🔾 | 🔾 | 🔾 | 🔾 | 🔾 | 🔾 | 🔾 | 🔾 | 🔾 | 🔾 |
| Contact sport, wrestling, judo | 🔾 | 🔾 | 🔾 | 🔾 | 🔾 | 🔾 | 🔾 | 🔾 | 🔾 | 🔾 | 🔾 | 🔾 | 🔾 | 🔾 | 🔾 |
| Computer, TV exercises, e.g. Wii Fit | 🔾 | 🔾 | 🔾 | 🔾 | 🔾 | 🔾 | 🔾 | 🔾 | 🔾 | 🔾 | 🔾 | 🔾 | 🔾 | 🔾 | 🔾 |
| Mixed exercises, cross training | 🔾 | 🔾 | 🔾 | 🔾 | 🔾 | 🔾 | 🔾 | 🔾 | 🔾 | 🔾 | 🔾 | 🔾 | 🔾 | 🔾 | 🔾 |
| Other (Specify) | 🔾 | 🔾 | 🔾 | 🔾 | 🔾 | 🔾 | 🔾 | 🔾 | 🔾 | 🔾 | 🔾 | 🔾 | 🔾 | 🔾 | 🔾 |
|  | After Onset of MS | | | | | | | | | | | | | | |
|  | Never | Once a Year | 2-4 Times a Year | 5-6 Times a Year | 7-11 Times a Year | Once a Month | 2-3 Times Month | Once a Week | 2-4 Times a Week | 5-6 Times a Week | Once a Day | 2 Times a Day | 3 or More Times a Day | Not Applicable at this Stage | Declined |
| Strenuous job-related walking | 🔾 | 🔾 | 🔾 | 🔾 | 🔾 | 🔾 | 🔾 | 🔾 | 🔾 | 🔾 | 🔾 | 🔾 | 🔾 | 🔾 | 🔾 |
| Strenuous job-related lifting, moving | 🔾 | 🔾 | 🔾 | 🔾 | 🔾 | 🔾 | 🔾 | 🔾 | 🔾 | 🔾 | 🔾 | 🔾 | 🔾 | 🔾 | 🔾 |
| Yoga, pilates, flexibility training | 🔾 | 🔾 | 🔾 | 🔾 | 🔾 | 🔾 | 🔾 | 🔾 | 🔾 | 🔾 | 🔾 | 🔾 | 🔾 | 🔾 | 🔾 |
| Weights, strength training | 🔾 | 🔾 | 🔾 | 🔾 | 🔾 | 🔾 | 🔾 | 🔾 | 🔾 | 🔾 | 🔾 | 🔾 | 🔾 | 🔾 | 🔾 |
| Tai Chi | 🔾 | 🔾 | 🔾 | 🔾 | 🔾 | 🔾 | 🔾 | 🔾 | 🔾 | 🔾 | 🔾 | 🔾 | 🔾 | 🔾 | 🔾 |
| Aerobics exercises | 🔾 | 🔾 | 🔾 | 🔾 | 🔾 | 🔾 | 🔾 | 🔾 | 🔾 | 🔾 | 🔾 | 🔾 | 🔾 | 🔾 | 🔾 |
| Running | 🔾 | 🔾 | 🔾 | 🔾 | 🔾 | 🔾 | 🔾 | 🔾 | 🔾 | 🔾 | 🔾 | 🔾 | 🔾 | 🔾 | 🔾 |
| Walking | 🔾 | 🔾 | 🔾 | 🔾 | 🔾 | 🔾 | 🔾 | 🔾 | 🔾 | 🔾 | 🔾 | 🔾 | 🔾 | 🔾 | 🔾 |
| Swimming | 🔾 | 🔾 | 🔾 | 🔾 | 🔾 | 🔾 | 🔾 | 🔾 | 🔾 | 🔾 | 🔾 | 🔾 | 🔾 | 🔾 | 🔾 |
| Other aerobic, Skiing, climbing, rowing | 🔾 | 🔾 | 🔾 | 🔾 | 🔾 | 🔾 | 🔾 | 🔾 | 🔾 | 🔾 | 🔾 | 🔾 | 🔾 | 🔾 | 🔾 |
| Court sports, e.g., tennis, basketball, racketball, squash | 🔾 | 🔾 | 🔾 | 🔾 | 🔾 | 🔾 | 🔾 | 🔾 | 🔾 | 🔾 | 🔾 | 🔾 | 🔾 | 🔾 | 🔾 |
| Field sport, soccer, lacrosse, field hockey | 🔾 | 🔾 | 🔾 | 🔾 | 🔾 | 🔾 | 🔾 | 🔾 | 🔾 | 🔾 | 🔾 | 🔾 | 🔾 | 🔾 | 🔾 |
| Contact field sport, rugby, football | 🔾 | 🔾 | 🔾 | 🔾 | 🔾 | 🔾 | 🔾 | 🔾 | 🔾 | 🔾 | 🔾 | 🔾 | 🔾 | 🔾 | 🔾 |
| Boxing | 🔾 | 🔾 | 🔾 | 🔾 | 🔾 | 🔾 | 🔾 | 🔾 | 🔾 | 🔾 | 🔾 | 🔾 | 🔾 | 🔾 | 🔾 |
| Contact sport, wrestling, judo | 🔾 | 🔾 | 🔾 | 🔾 | 🔾 | 🔾 | 🔾 | 🔾 | 🔾 | 🔾 | 🔾 | 🔾 | 🔾 | 🔾 | 🔾 |
| Computer, TV exercises, e.g. Wii Fit | 🔾 | 🔾 | 🔾 | 🔾 | 🔾 | 🔾 | 🔾 | 🔾 | 🔾 | 🔾 | 🔾 | 🔾 | 🔾 | 🔾 | 🔾 |
| Mixed exercises, cross training | 🔾 | 🔾 | 🔾 | 🔾 | 🔾 | 🔾 | 🔾 | 🔾 | 🔾 | 🔾 | 🔾 | 🔾 | 🔾 | 🔾 | 🔾 |
| Other (Specify) | 🔾 | 🔾 | 🔾 | 🔾 | 🔾 | 🔾 | 🔾 | 🔾 | 🔾 | 🔾 | 🔾 | 🔾 | 🔾 | 🔾 | 🔾 |

|  | Last 3-Months | | | | | | | | | | | | | | |
| --- | --- | --- | --- | --- | --- | --- | --- | --- | --- | --- | --- | --- | --- | --- | --- |
|  | Never | Once a Year | 2-4 Times a Year | 5-6 Times a Year | 7-11 Times a Year | Once a Month | 2-3 Times Month | Once a Week | 2-4 Times a Week | 5-6 Times a Week | Once a Day | 2 Times a Day | 3 or More Times a Day | Not Applicable at this Stage | Declined |
| Strenuous job-related walking | 🔾 | 🔾 | 🔾 | 🔾 | 🔾 | 🔾 | 🔾 | 🔾 | 🔾 | 🔾 | 🔾 | 🔾 | 🔾 | 🔾 | 🔾 |
| Strenuous job-related lifting, moving | 🔾 | 🔾 | 🔾 | 🔾 | 🔾 | 🔾 | 🔾 | 🔾 | 🔾 | 🔾 | 🔾 | 🔾 | 🔾 | 🔾 | 🔾 |
| Yoga, pilates, flexibility training | 🔾 | 🔾 | 🔾 | 🔾 | 🔾 | 🔾 | 🔾 | 🔾 | 🔾 | 🔾 | 🔾 | 🔾 | 🔾 | 🔾 | 🔾 |
| Weights, strength training | 🔾 | 🔾 | 🔾 | 🔾 | 🔾 | 🔾 | 🔾 | 🔾 | 🔾 | 🔾 | 🔾 | 🔾 | 🔾 | 🔾 | 🔾 |
| Tai Chi | 🔾 | 🔾 | 🔾 | 🔾 | 🔾 | 🔾 | 🔾 | 🔾 | 🔾 | 🔾 | 🔾 | 🔾 | 🔾 | 🔾 | 🔾 |
| Aerobics exercises | 🔾 | 🔾 | 🔾 | 🔾 | 🔾 | 🔾 | 🔾 | 🔾 | 🔾 | 🔾 | 🔾 | 🔾 | 🔾 | 🔾 | 🔾 |
| Running | 🔾 | 🔾 | 🔾 | 🔾 | 🔾 | 🔾 | 🔾 | 🔾 | 🔾 | 🔾 | 🔾 | 🔾 | 🔾 | 🔾 | 🔾 |
| Walking | 🔾 | 🔾 | 🔾 | 🔾 | 🔾 | 🔾 | 🔾 | 🔾 | 🔾 | 🔾 | 🔾 | 🔾 | 🔾 | 🔾 | 🔾 |
| Swimming | 🔾 | 🔾 | 🔾 | 🔾 | 🔾 | 🔾 | 🔾 | 🔾 | 🔾 | 🔾 | 🔾 | 🔾 | 🔾 | 🔾 | 🔾 |
| Other aerobic, Skiing, climbing, rowing | 🔾 | 🔾 | 🔾 | 🔾 | 🔾 | 🔾 | 🔾 | 🔾 | 🔾 | 🔾 | 🔾 | 🔾 | 🔾 | 🔾 | 🔾 |
| Court sports, e.g., tennis, basketball, racketball, squash | 🔾 | 🔾 | 🔾 | 🔾 | 🔾 | 🔾 | 🔾 | 🔾 | 🔾 | 🔾 | 🔾 | 🔾 | 🔾 | 🔾 | 🔾 |
| Field sport, soccer, lacrosse, field hockey | 🔾 | 🔾 | 🔾 | 🔾 | 🔾 | 🔾 | 🔾 | 🔾 | 🔾 | 🔾 | 🔾 | 🔾 | 🔾 | 🔾 | 🔾 |
| Contact field sport, rugby, football | 🔾 | 🔾 | 🔾 | 🔾 | 🔾 | 🔾 | 🔾 | 🔾 | 🔾 | 🔾 | 🔾 | 🔾 | 🔾 | 🔾 | 🔾 |
| Boxing | 🔾 | 🔾 | 🔾 | 🔾 | 🔾 | 🔾 | 🔾 | 🔾 | 🔾 | 🔾 | 🔾 | 🔾 | 🔾 | 🔾 | 🔾 |
| Contact sport, wrestling, judo | 🔾 | 🔾 | 🔾 | 🔾 | 🔾 | 🔾 | 🔾 | 🔾 | 🔾 | 🔾 | 🔾 | 🔾 | 🔾 | 🔾 | 🔾 |
| Computer, TV exercises, e.g. Wii Fit | 🔾 | 🔾 | 🔾 | 🔾 | 🔾 | 🔾 | 🔾 | 🔾 | 🔾 | 🔾 | 🔾 | 🔾 | 🔾 | 🔾 | 🔾 |
| Mixed exercises, cross training | 🔾 | 🔾 | 🔾 | 🔾 | 🔾 | 🔾 | 🔾 | 🔾 | 🔾 | 🔾 | 🔾 | 🔾 | 🔾 | 🔾 | 🔾 |
| Other (Specify) | 🔾 | 🔾 | 🔾 | 🔾 | 🔾 | 🔾 | 🔾 | 🔾 | 🔾 | 🔾 | 🔾 | 🔾 | 🔾 | 🔾 | 🔾 |

**76) Stressful and Tragic Events.**

|  | Non-MS Subject or Before Onset of MS | | | | | | | | |
| --- | --- | --- | --- | --- | --- | --- | --- | --- | --- |
|  | Never | Once | Twice | 3 Times | 4 or More Times | Not Applicable, Male | Not Applicable, Minor | Do Not Remember | Declined |
| Military combat | 🔾 | 🔾 | 🔾 | 🔾 | 🔾 | 🔾 | 🔾 | 🔾 | 🔾 |
| Divorce of parents | 🔾 | 🔾 | 🔾 | 🔾 | 🔾 | 🔾 | 🔾 | 🔾 | 🔾 |
| Loss of parent | 🔾 | 🔾 | 🔾 | 🔾 | 🔾 | 🔾 | 🔾 | 🔾 | 🔾 |
| Loss of newborn child | 🔾 | 🔾 | 🔾 | 🔾 | 🔾 | 🔾 | 🔾 | 🔾 | 🔾 |
| First trimester miscarriages | 🔾 | 🔾 | 🔾 | 🔾 | 🔾 | 🔾 | 🔾 | 🔾 | 🔾 |
| Second or third trimester miscarriages | 🔾 | 🔾 | 🔾 | 🔾 | 🔾 | 🔾 | 🔾 | 🔾 | 🔾 |
| Loss of child under 10 years | 🔾 | 🔾 | 🔾 | 🔾 | 🔾 | 🔾 | 🔾 | 🔾 | 🔾 |
| Loss of child 10-18 years | 🔾 | 🔾 | 🔾 | 🔾 | 🔾 | 🔾 | 🔾 | 🔾 | 🔾 |
| Loss of adult child | 🔾 | 🔾 | 🔾 | 🔾 | 🔾 | 🔾 | 🔾 | 🔾 | 🔾 |
| Loss of sibling | 🔾 | 🔾 | 🔾 | 🔾 | 🔾 | 🔾 | 🔾 | 🔾 | 🔾 |
| Homelessness | 🔾 | 🔾 | 🔾 | 🔾 | 🔾 | 🔾 | 🔾 | 🔾 | 🔾 |
| Bankruptcy, foreclosure on parents | 🔾 | 🔾 | 🔾 | 🔾 | 🔾 | 🔾 | 🔾 | 🔾 | 🔾 |
| Bankruptcy, foreclosure on self | 🔾 | 🔾 | 🔾 | 🔾 | 🔾 | 🔾 | 🔾 | 🔾 | 🔾 |
| Job loss | 🔾 | 🔾 | 🔾 | 🔾 | 🔾 | 🔾 | 🔾 | 🔾 | 🔾 |
| Homicide in home | 🔾 | 🔾 | 🔾 | 🔾 | 🔾 | 🔾 | 🔾 | 🔾 | 🔾 |
| Life threatening illness in family | 🔾 | 🔾 | 🔾 | 🔾 | 🔾 | 🔾 | 🔾 | 🔾 | 🔾 |
| Long-term (≥ 1 year) physical disability in immediate family | 🔾 | 🔾 | 🔾 | 🔾 | 🔾 | 🔾 | 🔾 | 🔾 | 🔾 |
| Long-term (≥ 1 year) cognitive disability in family | 🔾 | 🔾 | 🔾 | 🔾 | 🔾 | 🔾 | 🔾 | 🔾 | 🔾 |
| Criminal legal proceedings | 🔾 | 🔾 | 🔾 | 🔾 | 🔾 | 🔾 | 🔾 | 🔾 | 🔾 |
| Civil legal proceedings | 🔾 | 🔾 | 🔾 | 🔾 | 🔾 | 🔾 | 🔾 | 🔾 | 🔾 |
| Prison sentence | 🔾 | 🔾 | 🔾 | 🔾 | 🔾 | 🔾 | 🔾 | 🔾 | 🔾 |
| Other | 🔾 | 🔾 | 🔾 | 🔾 | 🔾 | 🔾 | 🔾 | 🔾 | 🔾 |

|  | After Onset of MS | | | | | | | | |
| --- | --- | --- | --- | --- | --- | --- | --- | --- | --- |
|  | Never | Once | Twice | 3 Times | 4 or More Times | Not Applicable, Male | Not Applicable, Minor | Do Not Remember | Declined |
| Military combat | 🔾 | 🔾 | 🔾 | 🔾 | 🔾 | 🔾 | 🔾 | 🔾 | 🔾 |
| Divorce of parents | 🔾 | 🔾 | 🔾 | 🔾 | 🔾 | 🔾 | 🔾 | 🔾 | 🔾 |
| Loss of parent | 🔾 | 🔾 | 🔾 | 🔾 | 🔾 | 🔾 | 🔾 | 🔾 | 🔾 |
| Loss of newborn child | 🔾 | 🔾 | 🔾 | 🔾 | 🔾 | 🔾 | 🔾 | 🔾 | 🔾 |
| First trimester miscarriages | 🔾 | 🔾 | 🔾 | 🔾 | 🔾 | 🔾 | 🔾 | 🔾 | 🔾 |
| Second or third trimester miscarriages | 🔾 | 🔾 | 🔾 | 🔾 | 🔾 | 🔾 | 🔾 | 🔾 | 🔾 |
| Loss of child under 10 years | 🔾 | 🔾 | 🔾 | 🔾 | 🔾 | 🔾 | 🔾 | 🔾 | 🔾 |
| Loss of child 10-18 years | 🔾 | 🔾 | 🔾 | 🔾 | 🔾 | 🔾 | 🔾 | 🔾 | 🔾 |
| Loss of adult child | 🔾 | 🔾 | 🔾 | 🔾 | 🔾 | 🔾 | 🔾 | 🔾 | 🔾 |
| Loss of sibling | 🔾 | 🔾 | 🔾 | 🔾 | 🔾 | 🔾 | 🔾 | 🔾 | 🔾 |
| Homelessness | 🔾 | 🔾 | 🔾 | 🔾 | 🔾 | 🔾 | 🔾 | 🔾 | 🔾 |
| Bankruptcy, foreclosure on parents | 🔾 | 🔾 | 🔾 | 🔾 | 🔾 | 🔾 | 🔾 | 🔾 | 🔾 |
| Bankruptcy, foreclosure on self | 🔾 | 🔾 | 🔾 | 🔾 | 🔾 | 🔾 | 🔾 | 🔾 | 🔾 |
| Job loss | 🔾 | 🔾 | 🔾 | 🔾 | 🔾 | 🔾 | 🔾 | 🔾 | 🔾 |
| Homicide in home | 🔾 | 🔾 | 🔾 | 🔾 | 🔾 | 🔾 | 🔾 | 🔾 | 🔾 |
| Life threatening illness in family | 🔾 | 🔾 | 🔾 | 🔾 | 🔾 | 🔾 | 🔾 | 🔾 | 🔾 |
| Long-term (≥ 1 year) physical disability in immediate family | 🔾 | 🔾 | 🔾 | 🔾 | 🔾 | 🔾 | 🔾 | 🔾 | 🔾 |
| Long-term (≥ 1 year) cognitive disability in family | 🔾 | 🔾 | 🔾 | 🔾 | 🔾 | 🔾 | 🔾 | 🔾 | 🔾 |
| Criminal legal proceedings | 🔾 | 🔾 | 🔾 | 🔾 | 🔾 | 🔾 | 🔾 | 🔾 | 🔾 |
| Civil legal proceedings | 🔾 | 🔾 | 🔾 | 🔾 | 🔾 | 🔾 | 🔾 | 🔾 | 🔾 |
| Prison sentence | 🔾 | 🔾 | 🔾 | 🔾 | 🔾 | 🔾 | 🔾 | 🔾 | 🔾 |
| Other | 🔾 | 🔾 | 🔾 | 🔾 | 🔾 | 🔾 | 🔾 | 🔾 | 🔾 |

|  | Last 3 Months? | | | | | | | | |
| --- | --- | --- | --- | --- | --- | --- | --- | --- | --- |
|  | Never | Once | Twice | 3 Times | 4 or More Times | Not Applicable, Male | Not Applicable, Minor | Do Not Remember | Declined |
| Military combat | 🔾 | 🔾 | 🔾 | 🔾 | 🔾 | 🔾 | 🔾 | 🔾 | 🔾 |
| Divorce of parents | 🔾 | 🔾 | 🔾 | 🔾 | 🔾 | 🔾 | 🔾 | 🔾 | 🔾 |
| Loss of parent | 🔾 | 🔾 | 🔾 | 🔾 | 🔾 | 🔾 | 🔾 | 🔾 | 🔾 |
| Loss of newborn child | 🔾 | 🔾 | 🔾 | 🔾 | 🔾 | 🔾 | 🔾 | 🔾 | 🔾 |
| First trimester miscarriages | 🔾 | 🔾 | 🔾 | 🔾 | 🔾 | 🔾 | 🔾 | 🔾 | 🔾 |
| Second or third trimester miscarriages | 🔾 | 🔾 | 🔾 | 🔾 | 🔾 | 🔾 | 🔾 | 🔾 | 🔾 |
| Loss of child under 10 years | 🔾 | 🔾 | 🔾 | 🔾 | 🔾 | 🔾 | 🔾 | 🔾 | 🔾 |
| Loss of child 10-18 years | 🔾 | 🔾 | 🔾 | 🔾 | 🔾 | 🔾 | 🔾 | 🔾 | 🔾 |
| Loss of adult child | 🔾 | 🔾 | 🔾 | 🔾 | 🔾 | 🔾 | 🔾 | 🔾 | 🔾 |
| Loss of sibling | 🔾 | 🔾 | 🔾 | 🔾 | 🔾 | 🔾 | 🔾 | 🔾 | 🔾 |
| Homelessness | 🔾 | 🔾 | 🔾 | 🔾 | 🔾 | 🔾 | 🔾 | 🔾 | 🔾 |
| Bankruptcy, foreclosure on parents | 🔾 | 🔾 | 🔾 | 🔾 | 🔾 | 🔾 | 🔾 | 🔾 | 🔾 |
| Bankruptcy, foreclosure on self | 🔾 | 🔾 | 🔾 | 🔾 | 🔾 | 🔾 | 🔾 | 🔾 | 🔾 |
| Job loss | 🔾 | 🔾 | 🔾 | 🔾 | 🔾 | 🔾 | 🔾 | 🔾 | 🔾 |
| Homicide in home | 🔾 | 🔾 | 🔾 | 🔾 | 🔾 | 🔾 | 🔾 | 🔾 | 🔾 |
| Life threatening illness in family | 🔾 | 🔾 | 🔾 | 🔾 | 🔾 | 🔾 | 🔾 | 🔾 | 🔾 |
| Long-term (≥ 1 year) physical disability in immediate family | 🔾 | 🔾 | 🔾 | 🔾 | 🔾 | 🔾 | 🔾 | 🔾 | 🔾 |
| Long-term (≥ 1 year) cognitive disability in family | 🔾 | 🔾 | 🔾 | 🔾 | 🔾 | 🔾 | 🔾 | 🔾 | 🔾 |
| Criminal legal proceedings | 🔾 | 🔾 | 🔾 | 🔾 | 🔾 | 🔾 | 🔾 | 🔾 | 🔾 |
| Civil legal proceedings | 🔾 | 🔾 | 🔾 | 🔾 | 🔾 | 🔾 | 🔾 | 🔾 | 🔾 |
| Prison sentence | 🔾 | 🔾 | 🔾 | 🔾 | 🔾 | 🔾 | 🔾 | 🔾 | 🔾 |
| Other | 🔾 | 🔾 | 🔾 | 🔾 | 🔾 | 🔾 | 🔾 | 🔾 | 🔾 |

**77) Other Diseases You have or Others in Your Immediate Family and Siblings Have (Check all that apply).**

|  | Myself | Father | Mother | Son | Daughter | Full Brother | Full Sister | Half Brother | Half Sister | Step or Adopted Brother | Step or Adopted Sister | Other |
| --- | --- | --- | --- | --- | --- | --- | --- | --- | --- | --- | --- | --- |
| Allergies | ❑ | ❑ | ❑ | ❑ | ❑ | ❑ | ❑ | ❑ | ❑ | ❑ | ❑ | ❑ |
| Asthma | ❑ | ❑ | ❑ | ❑ | ❑ | ❑ | ❑ | ❑ | ❑ | ❑ | ❑ | ❑ |
| Cancer | ❑ | ❑ | ❑ | ❑ | ❑ | ❑ | ❑ | ❑ | ❑ | ❑ | ❑ | ❑ |
| Lymphoma | ❑ | ❑ | ❑ | ❑ | ❑ | ❑ | ❑ | ❑ | ❑ | ❑ | ❑ | ❑ |
| Chronic respiratory diseases | ❑ | ❑ | ❑ | ❑ | ❑ | ❑ | ❑ | ❑ | ❑ | ❑ | ❑ | ❑ |
| Lupus erythematosus | ❑ | ❑ | ❑ | ❑ | ❑ | ❑ | ❑ | ❑ | ❑ | ❑ | ❑ | ❑ |
| Rheumatoid disorders | ❑ | ❑ | ❑ | ❑ | ❑ | ❑ | ❑ | ❑ | ❑ | ❑ | ❑ | ❑ |
| Type 1 Diabetes | ❑ | ❑ | ❑ | ❑ | ❑ | ❑ | ❑ | ❑ | ❑ | ❑ | ❑ | ❑ |
| Crohn's disease | ❑ | ❑ | ❑ | ❑ | ❑ | ❑ | ❑ | ❑ | ❑ | ❑ | ❑ | ❑ |
| Irritable bowel disease | ❑ | ❑ | ❑ | ❑ | ❑ | ❑ | ❑ | ❑ | ❑ | ❑ | ❑ | ❑ |
| Migraines | ❑ | ❑ | ❑ | ❑ | ❑ | ❑ | ❑ | ❑ | ❑ | ❑ | ❑ | ❑ |
| Myasthenia gravis | ❑ | ❑ | ❑ | ❑ | ❑ | ❑ | ❑ | ❑ | ❑ | ❑ | ❑ | ❑ |
| Psoriasis | ❑ | ❑ | ❑ | ❑ | ❑ | ❑ | ❑ | ❑ | ❑ | ❑ | ❑ | ❑ |

**78) Other diseases in Blood Relatives (Check all that apply)**

|  | Paternal Grandfather | Paternal Grandmother | Maternal Grandfather | Maternal Grandmother | Paternal Uncle | Paternal Aunt | Maternal Uncle | Maternal Aunt | Paternal Cousin | Maternal Cousin | Other |
| --- | --- | --- | --- | --- | --- | --- | --- | --- | --- | --- | --- |
| Allergies | ❑ | ❑ | ❑ | ❑ | ❑ | ❑ | ❑ | ❑ | ❑ | ❑ | ❑ |
| Asthma | ❑ | ❑ | ❑ | ❑ | ❑ | ❑ | ❑ | ❑ | ❑ | ❑ | ❑ |
| Cancer | ❑ | ❑ | ❑ | ❑ | ❑ | ❑ | ❑ | ❑ | ❑ | ❑ | ❑ |
| Lymphoma | ❑ | ❑ | ❑ | ❑ | ❑ | ❑ | ❑ | ❑ | ❑ | ❑ | ❑ |
| Chronic respiratory diseases | ❑ | ❑ | ❑ | ❑ | ❑ | ❑ | ❑ | ❑ | ❑ | ❑ | ❑ |
| Lupus erythematosus | ❑ | ❑ | ❑ | ❑ | ❑ | ❑ | ❑ | ❑ | ❑ | ❑ | ❑ |
| Rheumatoid disorders | ❑ | ❑ | ❑ | ❑ | ❑ | ❑ | ❑ | ❑ | ❑ | ❑ | ❑ |
| Type 1 Diabetes | ❑ | ❑ | ❑ | ❑ | ❑ | ❑ | ❑ | ❑ | ❑ | ❑ | ❑ |
| Crohn's disease | ❑ | ❑ | ❑ | ❑ | ❑ | ❑ | ❑ | ❑ | ❑ | ❑ | ❑ |
| Irritable bowel disease | ❑ | ❑ | ❑ | ❑ | ❑ | ❑ | ❑ | ❑ | ❑ | ❑ | ❑ |
| Migraines | ❑ | ❑ | ❑ | ❑ | ❑ | ❑ | ❑ | ❑ | ❑ | ❑ | ❑ |
| Myasthenia gravis | ❑ | ❑ | ❑ | ❑ | ❑ | ❑ | ❑ | ❑ | ❑ | ❑ | ❑ |
| Psoriasis | ❑ | ❑ | ❑ | ❑ | ❑ | ❑ | ❑ | ❑ | ❑ | ❑ | ❑ |

**79) Smoking and Second hand smoke**

|  | Non-MS Subject or Before Onset of MS? | | | After Onset of MS? | | | Last 3-months? | | |
| --- | --- | --- | --- | --- | --- | --- | --- | --- | --- |
|  | Yes | No | Declined | Yes | No | Declined | Yes | No | Declined |
| Did you ever smoke more than 100 cigarettes? | 🔾 | 🔾 | 🔾 | 🔾 | 🔾 | 🔾 | 🔾 | 🔾 | 🔾 |
| Did you smoke regularly? | 🔾 | 🔾 | 🔾 | 🔾 | 🔾 | 🔾 | 🔾 | 🔾 | 🔾 |
| Were you exposed to more than 100 cigarettes of second hand smoke | 🔾 | 🔾 | 🔾 | 🔾 | 🔾 | 🔾 | 🔾 | 🔾 | 🔾 |

**80) If you did not smoke, were you regularly exposed to Second-Hand Smoke (at Home or Work, e.g., Restaurant or Bar) corresponding to 100 or more cigarettes?**

|  | Non-MS Subject or Before Onset of MS? | | | | | | | | | | | | | |
| --- | --- | --- | --- | --- | --- | --- | --- | --- | --- | --- | --- | --- | --- | --- |
|  | N/A | Not exposed to smoke at this stage | 3 months or less | 3.01 to 6 months | 6.01 months to 1 year | 1.01 year to 2 years | 2.01 to 5 years | 5.01 years to 10 years | 10 years to 15 years | 16 years to 20 years | More than 20 years | Other (Specify) | Do Not Remember | Declined |
| Second Hand Smoke Exposure | 🔾 | 🔾 | 🔾 | 🔾 | 🔾 | 🔾 | 🔾 | 🔾 | 🔾 | 🔾 | 🔾 | 🔾 | 🔾 | 🔾 |

|  | After Onset of MS?? | | | | | | | | | | | | | |
| --- | --- | --- | --- | --- | --- | --- | --- | --- | --- | --- | --- | --- | --- | --- |
|  | N/A | Not exposed to smoke at this stage | 3 months or less | 3.01 to 6 months | 6.01 months to 1 year | 1.01 year to 2 years | 2.01 to 5 years | 5.01 years to 10 years | 10 years to 15 years | 16 years to 20 years | More than 20 years | Other (Specify) | Do Not Remember | Declined |
| Second Hand Smoke Exposure | 🔾 | 🔾 | 🔾 | 🔾 | 🔾 | 🔾 | 🔾 | 🔾 | 🔾 | 🔾 | 🔾 | 🔾 | 🔾 | 🔾 |

|  | Last 3-Months | | | | | | | | | | | | | |
| --- | --- | --- | --- | --- | --- | --- | --- | --- | --- | --- | --- | --- | --- | --- |
|  | N/A | Not exposed to smoke at this stage | 3 months or less | 3.01 to 6 months | 6.01 months to 1 year | 1.01 year to 2 years | 2.01 to 5 years | 5.01 years to 10 years | 10 years to 15 years | 16 years to 20 years | More than 20 years | Other (Specify) | Do Not Remember | Declined |
| Second Hand Smoke Exposure | 🔾 | 🔾 | 🔾 | 🔾 | 🔾 | 🔾 | 🔾 | 🔾 | 🔾 | 🔾 | 🔾 | 🔾 | 🔾 | 🔾 |

**81) Thinking about the whole time you smoked regularly, how many cigarettes per day did you smoke? One Pack is 20 cigarettes.**

|  | Non-MS Subject or Before Onset of MS? | | | | | | | | | | | | |
| --- | --- | --- | --- | --- | --- | --- | --- | --- | --- | --- | --- | --- | --- |
|  | N/A | Did not smoke during this stage | 1 pack or less per Week | 1-5 cigarettes per Day | 6-10 cigarettes per Day | 11-15 cigarettes per Day | 16-20 cigarettes (1 Pack) per Day | 21-30 cigarettes (1.5 packs) per Day | 31 cigarettes-2 Packs per day | 41 Cigarettes to 3 Packs per day | More than 3 packs per day | Smoked cigars, beedies or other product | Used smokeless tobacco product |
| Frequency | 🔾 | 🔾 | 🔾 | 🔾 | 🔾 | 🔾 | 🔾 | 🔾 | 🔾 | 🔾 | 🔾 | 🔾 | 🔾 |

|  | After Onset of MS? | | | | | | | | | | | | |
| --- | --- | --- | --- | --- | --- | --- | --- | --- | --- | --- | --- | --- | --- |
|  | N/A | Did not smoke during this stage | 1 pack or less per Week | 1-5 cigarettes per Day | 6-10 cigarettes per Day | 11-15 cigarettes per Day | 16-20 cigarettes (1 Pack) per Day | 21-30 cigarettes (1.5 packs) per Day | 31 cigarettes-2 Packs per day | 41 Cigarettes to 3 Packs per day | More than 3 packs per day | Smoked cigars, beedies or other product | Used smokeless tobacco product |
| Frequency | 🔾 | 🔾 | 🔾 | 🔾 | 🔾 | 🔾 | 🔾 | 🔾 | 🔾 | 🔾 | 🔾 | 🔾 | 🔾 |

|  | Last 3 Months? | | | | | | | | | | | | |
| --- | --- | --- | --- | --- | --- | --- | --- | --- | --- | --- | --- | --- | --- |
|  | N/A | Did not smoke during this stage | 1 pack or less per Week | 1-5 cigarettes per Day | 6-10 cigarettes per Day | 11-15 cigarettes per Day | 16-20 cigarettes (1 Pack) per Day | 21-30 cigarettes (1.5 packs) per Day | 31 cigarettes-2 Packs per day | 41 Cigarettes to 3 Packs per day | More than 3 packs per day | Smoked cigars, beedies or other product | Used smokeless tobacco product |
| Frequency | 🔾 | 🔾 | 🔾 | 🔾 | 🔾 | 🔾 | 🔾 | 🔾 | 🔾 | 🔾 | 🔾 | 🔾 | 🔾 |

**82) Thinking about the whole time you smoked regularly, how soon after waking did you usually smoke your first cigarette?**

|  | Non-MS Subject or Before Onset of MS? | | | | | | | | | | | |
| --- | --- | --- | --- | --- | --- | --- | --- | --- | --- | --- | --- | --- |
|  | N/A | Did not smoke during this stage | 5 minutes or less | 6 to 15 minutes | 16 minutes to 30 minutes | 31 minutes to 1 hour | 1.01 hours to 2 hours | 2.01 hours to 4 hours | 4 to 6 hours | More than 6 hours | Other | Declined |
| How soon? | 🔾 | 🔾 | 🔾 | 🔾 | 🔾 | 🔾 | 🔾 | 🔾 | 🔾 | 🔾 | 🔾 | 🔾 |

|  | After Onset of MS? | | | | | | | | | | | |
| --- | --- | --- | --- | --- | --- | --- | --- | --- | --- | --- | --- | --- |
|  | N/A | Did not smoke during this stage | 5 minutes or less | 6 to 15 minutes | 16 minutes to 30 minutes | 31 minutes to 1 hour | 1.01 hours to 2 hours | 2.01 hours to 4 hours | 4 to 6 hours | More than 6 hours | Other | Declined |
| How soon? | 🔾 | 🔾 | 🔾 | 🔾 | 🔾 | 🔾 | 🔾 | 🔾 | 🔾 | 🔾 | 🔾 | 🔾 |

|  | Last 3 Months? | | | | | | | | | | | |
| --- | --- | --- | --- | --- | --- | --- | --- | --- | --- | --- | --- | --- |
|  | N/A | Did not smoke during this stage | 5 minutes or less | 6 to 15 minutes | 16 minutes to 30 minutes | 31 minutes to 1 hour | 1.01 hours to 2 hours | 2.01 hours to 4 hours | 4 to 6 hours | More than 6 hours | Other | Declined |
| How soon? | 🔾 | 🔾 | 🔾 | 🔾 | 🔾 | 🔾 | 🔾 | 🔾 | 🔾 | 🔾 | 🔾 | 🔾 |

**83) Thinking about the whole time you smoked regularly, how many years did you smoke?**

|  | Non-MS Subject or Before Onset of MS? | | | | | | | | | | | |
| --- | --- | --- | --- | --- | --- | --- | --- | --- | --- | --- | --- | --- |
|  | N/A | Did not smoke during this stage | 3 months or less | 3.01 to 6 months | 6.01 months to 1 year | 1.01 year to 2 years | 2.01 to 5 years | 5.01 years to 10 years | 10 years to 15 years | 16 years to 20 years | More than 20 years | Declined |
| Duration of smoking | 🔾 | 🔾 | 🔾 | 🔾 | 🔾 | 🔾 | 🔾 | 🔾 | 🔾 | 🔾 | 🔾 | 🔾 |

|  | After Onset of MS? | | | | | | | | | | | |
| --- | --- | --- | --- | --- | --- | --- | --- | --- | --- | --- | --- | --- |
|  | N/A | Did not smoke during this stage | 3 months or less | 3.01 to 6 months | 6.01 months to 1 year | 1.01 year to 2 years | 2.01 to 5 years | 5.01 years to 10 years | 10 years to 15 years | 16 years to 20 years | More than 20 years | Declined |
| Duration of smoking | 🔾 | 🔾 | 🔾 | 🔾 | 🔾 | 🔾 | 🔾 | 🔾 | 🔾 | 🔾 | 🔾 | 🔾 |

|  | Last 3 Months? | | | | | | | | | | | |
| --- | --- | --- | --- | --- | --- | --- | --- | --- | --- | --- | --- | --- |
|  | N/A | Did not smoke during this stage | 3 months or less | 3.01 to 6 months | 6.01 months to 1 year | 1.01 year to 2 years | 2.01 to 5 years | 5.01 years to 10 years | 10 years to 15 years | 16 years to 20 years | More than 20 years | Declined |
| Duration of smoking | 🔾 | 🔾 | 🔾 | 🔾 | 🔾 | 🔾 | 🔾 | 🔾 | 🔾 | 🔾 | 🔾 | 🔾 |

**84) At what Age did you Start Smoking?**

|  | Non-MS Subject or Before Onset of MS? | | | | | | | | | | | | | |
| --- | --- | --- | --- | --- | --- | --- | --- | --- | --- | --- | --- | --- | --- | --- |
|  | 6 to 9 years | 10 to 12 years | 13 - 15 years | 16 to 18 year | 19 to 21 years | 22 to 25 years | 26 to 30 years | 31 to 40 years | 41 to 50 years | 51 years or above | Do not remember | Not Applicable at this stage | Not applicable | Declined |
| Age of Smoking | 🔾 | 🔾 | 🔾 | 🔾 | 🔾 | 🔾 | 🔾 | 🔾 | 🔾 | 🔾 | 🔾 | 🔾 | 🔾 | 🔾 |

|  | After Onset of MS? | | | | | | | | | | | | | |
| --- | --- | --- | --- | --- | --- | --- | --- | --- | --- | --- | --- | --- | --- | --- |
|  | 6 to 9 years | 10 to 12 years | 13 - 15 years | 16 to 18 year | 19 to 21 years | 22 to 25 years | 26 to 30 years | 31 to 40 years | 41 to 50 years | 51 years or above | Do not remember | Not Applicable at this stage | Not applicable | Declined |
| Age of Smoking | 🔾 | 🔾 | 🔾 | 🔾 | 🔾 | 🔾 | 🔾 | 🔾 | 🔾 | 🔾 | 🔾 | 🔾 | 🔾 | 🔾 |

**85) How many times have you Quit Smoking for More than 3 Months?**

|  | Non-MS Subject or Before Onset of MS? | | | | | | | | | | |
| --- | --- | --- | --- | --- | --- | --- | --- | --- | --- | --- | --- |
|  | Never Quit | Once | Twice | 3 to 5 times | 6 to 10 times | 10 to 20 times | More than 20 times | Do not remember | Not Applicable at this stage | Not applicable | Declined |
| Times Quit | 🔾 | 🔾 | 🔾 | 🔾 | 🔾 | 🔾 | 🔾 | 🔾 | 🔾 | 🔾 | 🔾 |

|  | After Onset of MS? | | | | | | | | | | |
| --- | --- | --- | --- | --- | --- | --- | --- | --- | --- | --- | --- |
|  | Never Quit | Once | Twice | 3 to 5 times | 6 to 10 times | 10 to 20 times | More than 20 times | Do not remember | Not Applicable at this stage | Not applicable | Declined |
| Times Quit | 🔾 | 🔾 | 🔾 | 🔾 | 🔾 | 🔾 | 🔾 | 🔾 | 🔾 | 🔾 | 🔾 |

**86) At what Age Did you last Quit Smoking for 3 Months or More?** _____________________

**87) Alcoholic Beverage Use.**

**Alcoholic Beverage refers to Beer, Wine, Hard Liquor etc. One Drink is defined as One 12-ounce Can or Bottle of Beer; a 4-Ounce Glass of Wine, a Drink with One Shot of Hard Liquor, or One 12-Ounce Bottle of Wine Cooler.**

**Have you had 12 Alcoholic Drinks in your Entire Life**

🔾 Yes 🔾 Declined

🔾 No 🔾 Other (please specify)

🔾 Not Applicable If you selected other, please specify _______________________________

**87) Thinking about the Whole Time you consumed Alcoholic Beverages, How Many Years did you Consume Alcoholic Beverages?**

|  | Non-MS Subject or Before Onset of MS? | | | | | | | | | | | |
| --- | --- | --- | --- | --- | --- | --- | --- | --- | --- | --- | --- | --- |
|  | N/A | Did not consume alcohol during this stage | 3 months or less | 3.01 to 6 months | 6.01 months to 1 year | 1.01 year to 2 years | 2.01 to 5 years | 5.01 years to 10 years | 10 years to 15 years | 16 years to 20 years | More than 20 years | Declined |
| Duration | 🔾 | 🔾 | 🔾 | 🔾 | 🔾 | 🔾 | 🔾 | 🔾 | 🔾 | 🔾 | 🔾 | 🔾 |

|  | After Onset of MS? | | | | | | | | | | | |
| --- | --- | --- | --- | --- | --- | --- | --- | --- | --- | --- | --- | --- |
|  | N/A | Did not consume alcohol during this stage | 3 months or less | 3.01 to 6 months | 6.01 months to 1 year | 1.01 year to 2 years | 2.01 to 5 years | 5.01 years to 10 years | 10 years to 15 years | 16 years to 20 years | More than 20 years | Declined |
| Duration | 🔾 | 🔾 | 🔾 | 🔾 | 🔾 | 🔾 | 🔾 | 🔾 | 🔾 | 🔾 | 🔾 | 🔾 |

|  | Last 3 Months? | | | | | | | | | | | |
| --- | --- | --- | --- | --- | --- | --- | --- | --- | --- | --- | --- | --- |
|  | N/A | Did not consume alcohol during this stage | 3 months or less | 3.01 to 6 months | 6.01 months to 1 year | 1.01 year to 2 years | 2.01 to 5 years | 5.01 years to 10 years | 10 years to 15 years | 16 years to 20 years | More than 20 years | Declined |
| Duration | 🔾 | 🔾 | 🔾 | 🔾 | 🔾 | 🔾 | 🔾 | 🔾 | 🔾 | 🔾 | 🔾 | 🔾 |

**88) Thinking about the Whole Time you consumed Alcoholic Beverages, how Many Drinks did you consume?**

|  | Non-MS Subject or Before Onset of MS? | | | | | | | | | | | |
| --- | --- | --- | --- | --- | --- | --- | --- | --- | --- | --- | --- | --- |
|  | N/A | Did not consume alcohol during this stage | Never or Less than One Drink Per Month | 1 to 3 Drinks Per Month | One Drink Per Week | 2-4 Drinks Per Week | 5-6 Drinks Per Week | One Drink Per Day | 2-3 Drinks Per Day | 4 or More Drinks Per Day | Other | Declined |
| Number of Drinks | 🔾 | 🔾 | 🔾 | 🔾 | 🔾 | 🔾 | 🔾 | 🔾 | 🔾 | 🔾 | 🔾 | 🔾 |

|  | After Onset of MS? | | | | | | | | | | | |
| --- | --- | --- | --- | --- | --- | --- | --- | --- | --- | --- | --- | --- |
|  | N/A | Did not consume alcohol during this stage | Never or Less than One Drink Per Month | 1 to 3 Drinks Per Month | One Drink Per Week | 2-4 Drinks Per Week | 5-6 Drinks Per Week | One Drink Per Day | 2-3 Drinks Per Day | 4 or More Drinks Per Day | Other | Declined |
| Number of Drinks | 🔾 | 🔾 | 🔾 | 🔾 | 🔾 | 🔾 | 🔾 | 🔾 | 🔾 | 🔾 | 🔾 | 🔾 |

|  | Last 3 Months? | | | | | | | | | | | |
| --- | --- | --- | --- | --- | --- | --- | --- | --- | --- | --- | --- | --- |
|  | N/A | Did not consume alcohol during this stage | Never or Less than One Drink Per Month | 1 to 3 Drinks Per Month | One Drink Per Week | 2-4 Drinks Per Week | 5-6 Drinks Per Week | One Drink Per Day | 2-3 Drinks Per Day | 4 or More Drinks Per Day | Other | Declined |
| Number of Drinks | 🔾 | 🔾 | 🔾 | 🔾 | 🔾 | 🔾 | 🔾 | 🔾 | 🔾 | 🔾 | 🔾 | 🔾 |

**89) Thinking about the Whole Time you consumed Alcoholic Beverages, what was your Preferred Drink?**

|  | Non-MS Subject or Before Onset of MS? | | | | | | | | | | | | |
| --- | --- | --- | --- | --- | --- | --- | --- | --- | --- | --- | --- | --- | --- |
|  | N/A | Did not consume alcohol during this stage | Beer | Wine Cooler | Red Wine | White Wine | Sparkling Wine, Champagne | Whiskey | Vodka | Gin | Other Distilled Spirit | Other (specify) | Declined |
| Drink Type | 🔾 | 🔾 | 🔾 | 🔾 | 🔾 | 🔾 | 🔾 | 🔾 | 🔾 | 🔾 | 🔾 | 🔾 | 🔾 |

|  | After Onset of MS? | | | | | | | | | | | | |
| --- | --- | --- | --- | --- | --- | --- | --- | --- | --- | --- | --- | --- | --- |
|  | N/A | Did not consume alcohol during this stage | Beer | Wine Cooler | Red Wine | White Wine | Sparkling Wine, Champagne | Whiskey | Vodka | Gin | Other Distilled Spirit | Other (specify) | Declined |
| Drink Type | 🔾 | 🔾 | 🔾 | 🔾 | 🔾 | 🔾 | 🔾 | 🔾 | 🔾 | 🔾 | 🔾 | 🔾 | 🔾 |

|  | Last 3 Months? | | | | | | | | | | | | |
| --- | --- | --- | --- | --- | --- | --- | --- | --- | --- | --- | --- | --- | --- |
|  | N/A | Did not consume alcohol during this stage | Beer | Wine Cooler | Red Wine | White Wine | Sparkling Wine, Champagne | Whiskey | Vodka | Gin | Other Distilled Spirit | Other (specify) | Declined |
| Drink Type | 🔾 | 🔾 | 🔾 | 🔾 | 🔾 | 🔾 | 🔾 | 🔾 | 🔾 | 🔾 | 🔾 | 🔾 | 🔾 |

**90) Excluding small tastings, how Old were you when you Started Drinking Alcoholic Beverages?**

|  | Non-MS Subject or Before Onset of MS? | | | | | | | | | | | | | |
| --- | --- | --- | --- | --- | --- | --- | --- | --- | --- | --- | --- | --- | --- | --- |
|  | 6 to 9 years | 10 to 12 years | 13 - 15 years | 16 to 18 year | 19 to 21 years | 22 to 25 years | 26 to 30 years | 31 to 40 years | 41 to 50 years | 51 years or more | Do not remem-ber | Not Applicable at this stage | Not appli-cable | Declined |
| Age of Drinking | 🔾 | 🔾 | 🔾 | 🔾 | 🔾 | 🔾 | 🔾 | 🔾 | 🔾 | 🔾 | 🔾 | 🔾 | 🔾 | 🔾 |

|  | After Onset of MS? | | | | | | | | | | | | | |
| --- | --- | --- | --- | --- | --- | --- | --- | --- | --- | --- | --- | --- | --- | --- |
|  | 6 to 9 years | 10 to 12 years | 13 - 15 years | 16 to 18 year | 19 to 21 years | 22 to 25 years | 26 to 30 years | 31 to 40 years | 41 to 50 years | 51 years or more | Do not remem-ber | Not Applicable at this stage | Not appli-cable | Declined |
| Age of Drinking | 🔾 | 🔾 | 🔾 | 🔾 | 🔾 | 🔾 | 🔾 | 🔾 | 🔾 | 🔾 | 🔾 | 🔾 | 🔾 | 🔾 |

**91) Thinking about the whole time you consumed Alcoholic Beverages, how many Years did you Smoke AND consume Alcoholic Beverages?**

|  | Non-MS Subject or Before Onset of MS? | | | | | | | | | | | |
| --- | --- | --- | --- | --- | --- | --- | --- | --- | --- | --- | --- | --- |
|  | N/A | Did not smoke AND consume alcohol during this stage | 3 months or less | 3.01 to 6 months | 6.01 months to 1 year | 1.01 year to 2 years | 2.01 to 5 years | 5.01 years to 10 years | 10 years to 15 years | 16 years to 20 years | More than 20 years | Declined |
| Duration of Smoking & Alcoholic Beverages | 🔾 | 🔾 | 🔾 | 🔾 | 🔾 | 🔾 | 🔾 | 🔾 | 🔾 | 🔾 | 🔾 | 🔾 |

|  | After Onset of MS? | | | | | | | | | | | |
| --- | --- | --- | --- | --- | --- | --- | --- | --- | --- | --- | --- | --- |
|  | N/A | Did not smoke AND consume alcohol during this stage | 3 months or less | 3.01 to 6 months | 6.01 months to 1 year | 1.01 year to 2 years | 2.01 to 5 years | 5.01 years to 10 years | 10 years to 15 years | 16 years to 20 years | More than 20 years | Declined |
| Duration of Smoking & Alcoholic Beverages | 🔾 | 🔾 | 🔾 | 🔾 | 🔾 | 🔾 | 🔾 | 🔾 | 🔾 | 🔾 | 🔾 | 🔾 |

|  | Last 3 Months? | | | | | | | | | | | |
| --- | --- | --- | --- | --- | --- | --- | --- | --- | --- | --- | --- | --- |
|  | N/A | Did not smoke AND consume alcohol during this stage | 3 months or less | 3.01 to 6 months | 6.01 months to 1 year | 1.01 year to 2 years | 2.01 to 5 years | 5.01 years to 10 years | 10 years to 15 years | 16 years to 20 years | More than 20 years | Declined |
| Duration of Smoking & Alcoholic Beverages | 🔾 | 🔾 | 🔾 | 🔾 | 🔾 | 🔾 | 🔾 | 🔾 | 🔾 | 🔾 | 🔾 | 🔾 |

**92) Have you ever used Illicit Drugs such as Marijuana, Heroin, Cocaine, etc. on more than three occasions? Non-MS Subjects to answer questions on "Non-MS Subject or Before MS Onset" and "Used in Last Month"**

🔾 Yes 🔾 No 🔾 Declined

**93) Illicit Drugs**

|  | Non-MS Subject or Before Onset of MS? | | | After Onset of MS? | | | Last 3-months? | | |
| --- | --- | --- | --- | --- | --- | --- | --- | --- | --- |
|  | Yes | No | Declined | Yes | No | Declined | Yes | No | Declined |
| Marijuana or hashish | 🔾 | 🔾 | 🔾 | 🔾 | 🔾 | 🔾 | 🔾 | 🔾 | 🔾 |
| Amphetamines, e.g., Adderall, Benzedrine, Dexedrine, Methamphetamine, Speed, Dexies, Preludin, Yellow Jackets, Mini-Thins, Stackers | 🔾 | 🔾 | 🔾 | 🔾 | 🔾 | 🔾 | 🔾 | 🔾 | 🔾 |
| Gamma hydroxybutyrate, GHB | 🔾 | 🔾 | 🔾 | 🔾 | 🔾 | 🔾 | 🔾 | 🔾 | 🔾 |
| Epitestosterone | 🔾 | 🔾 | 🔾 | 🔾 | 🔾 | 🔾 | 🔾 | 🔾 | 🔾 |
| Erythropoeitin, EPO, Epoidin | 🔾 | 🔾 | 🔾 | 🔾 | 🔾 | 🔾 | 🔾 | 🔾 | 🔾 |
| Ecstasy, MDMA, Molly | 🔾 | 🔾 | 🔾 | 🔾 | 🔾 | 🔾 | 🔾 | 🔾 | 🔾 |
| Heroin | 🔾 | 🔾 | 🔾 | 🔾 | 🔾 | 🔾 | 🔾 | 🔾 | 🔾 |
| Cocaine or Crack | 🔾 | 🔾 | 🔾 | 🔾 | 🔾 | 🔾 | 🔾 | 🔾 | 🔾 |
| Inhalants or solvents (not Nitrites) | 🔾 | 🔾 | 🔾 | 🔾 | 🔾 | 🔾 | 🔾 | 🔾 | 🔾 |
| Prescription opiates and painkillers | 🔾 | 🔾 | 🔾 | 🔾 | 🔾 | 🔾 | 🔾 | 🔾 | 🔾 |
| Ketamine, Oxycontin | 🔾 | 🔾 | 🔾 | 🔾 | 🔾 | 🔾 | 🔾 | 🔾 | 🔾 |
| Human growth hormone | 🔾 | 🔾 | 🔾 | 🔾 | 🔾 | 🔾 | 🔾 | 🔾 | 🔾 |
| Amyl nitrite, butyl nitrite, Rush | 🔾 | 🔾 | 🔾 | 🔾 | 🔾 | 🔾 | 🔾 | 🔾 | 🔾 |
| Nitrous oxide, Whipits | 🔾 | 🔾 | 🔾 | 🔾 | 🔾 | 🔾 | 🔾 | 🔾 | 🔾 |
| Other Prescription drugs (Ritalin etc.) | 🔾 | 🔾 | 🔾 | 🔾 | 🔾 | 🔾 | 🔾 | 🔾 | 🔾 |
| Dimethyltrytamine, Deemsters | 🔾 | 🔾 | 🔾 | 🔾 | 🔾 | 🔾 | 🔾 | 🔾 | 🔾 |
| Salvia divinorum | 🔾 | 🔾 | 🔾 | 🔾 | 🔾 | 🔾 | 🔾 | 🔾 | 🔾 |
| Other | 🔾 | 🔾 | 🔾 | 🔾 | 🔾 | 🔾 | 🔾 | 🔾 | 🔾 |

**94) Comments and other drugs used:**

____________________________________________________________

**95) Have you ever used Dietary Supplements such as Vitamins, Calcium, Fish Oil tablets, Creatinine etc. regularly for 3 months or more?**

🔾 Yes 🔾 No 🔾 Declined

**96) Dietary Supplements**

|  | Non-MS Subject or Before Onset of MS? | | | After Onset of MS? | | | Last 3-months? | | |
| --- | --- | --- | --- | --- | --- | --- | --- | --- | --- |
|  | Yes | No | Declined | Yes | No | Declined | Yes | No | Declined |
| Amino acids | 🔾 | 🔾 | 🔾 | 🔾 | 🔾 | 🔾 | 🔾 | 🔾 | 🔾 |
| B-Complex | 🔾 | 🔾 | 🔾 | 🔾 | 🔾 | 🔾 | 🔾 | 🔾 | 🔾 |
| Calcium | 🔾 | 🔾 | 🔾 | 🔾 | 🔾 | 🔾 | 🔾 | 🔾 | 🔾 |
| Calcium with VItamin D | 🔾 | 🔾 | 🔾 | 🔾 | 🔾 | 🔾 | 🔾 | 🔾 | 🔾 |
| Chondroitin sulfate | 🔾 | 🔾 | 🔾 | 🔾 | 🔾 | 🔾 | 🔾 | 🔾 | 🔾 |
| Chromium picolinate or chromium | 🔾 | 🔾 | 🔾 | 🔾 | 🔾 | 🔾 | 🔾 | 🔾 | 🔾 |
| Creatinine | 🔾 | 🔾 | 🔾 | 🔾 | 🔾 | 🔾 | 🔾 | 🔾 | 🔾 |
| DHEA | 🔾 | 🔾 | 🔾 | 🔾 | 🔾 | 🔾 | 🔾 | 🔾 | 🔾 |
| Fish oil | 🔾 | 🔾 | 🔾 | 🔾 | 🔾 | 🔾 | 🔾 | 🔾 | 🔾 |
| Flaxseed oil | 🔾 | 🔾 | 🔾 | 🔾 | 🔾 | 🔾 | 🔾 | 🔾 | 🔾 |
| Folic acid | 🔾 | 🔾 | 🔾 | 🔾 | 🔾 | 🔾 | 🔾 | 🔾 | 🔾 |
| Iron supplements | 🔾 | 🔾 | 🔾 | 🔾 | 🔾 | 🔾 | 🔾 | 🔾 | 🔾 |
| Magnesium supplements | 🔾 | 🔾 | 🔾 | 🔾 | 🔾 | 🔾 | 🔾 | 🔾 | 🔾 |
| Melatonin | 🔾 | 🔾 | 🔾 | 🔾 | 🔾 | 🔾 | 🔾 | 🔾 | 🔾 |
| Multivitamin Tablet | 🔾 | 🔾 | 🔾 | 🔾 | 🔾 | 🔾 | 🔾 | 🔾 | 🔾 |
| Niacin (Vitamin B3) | 🔾 | 🔾 | 🔾 | 🔾 | 🔾 | 🔾 | 🔾 | 🔾 | 🔾 |
| Pantothenic acid (Vitamin B5) | 🔾 | 🔾 | 🔾 | 🔾 | 🔾 | 🔾 | 🔾 | 🔾 | 🔾 |
| Psyllium/Senna, Fiber | 🔾 | 🔾 | 🔾 | 🔾 | 🔾 | 🔾 | 🔾 | 🔾 | 🔾 |
| Selenium | 🔾 | 🔾 | 🔾 | 🔾 | 🔾 | 🔾 | 🔾 | 🔾 | 🔾 |
| Thiamin (Vitamin B1) | 🔾 | 🔾 | 🔾 | 🔾 | 🔾 | 🔾 | 🔾 | 🔾 | 🔾 |
| Vitamin B12 | 🔾 | 🔾 | 🔾 | 🔾 | 🔾 | 🔾 | 🔾 | 🔾 | 🔾 |
| Vitamin C | 🔾 | 🔾 | 🔾 | 🔾 | 🔾 | 🔾 | 🔾 | 🔾 | 🔾 |
| Vitamin D | 🔾 | 🔾 | 🔾 | 🔾 | 🔾 | 🔾 | 🔾 | 🔾 | 🔾 |
| Vitamin E | 🔾 | 🔾 | 🔾 | 🔾 | 🔾 | 🔾 | 🔾 | 🔾 | 🔾 |
| Vitamin K | 🔾 | 🔾 | 🔾 | 🔾 | 🔾 | 🔾 | 🔾 | 🔾 | 🔾 |
| Zinc | 🔾 | 🔾 | 🔾 | 🔾 | 🔾 | 🔾 | 🔾 | 🔾 | 🔾 |
| Other | 🔾 | 🔾 | 🔾 | 🔾 | 🔾 | 🔾 | 🔾 | 🔾 | 🔾 |

**97) What are your main reasons for using Dietary Supplements?**

____________________________________________________________

**98) Have you ever used any Herbal Supplements for more than a month? Herbal supplements can range from Bee Pollen, Padma Basic, Red Yeast Rice, etc.**

🔾 Yes 🔾 No 🔾 Declined

**99) Herbal Supplements**

|  | Non-MS Subject or Before Onset of MS? | | | After Onset of MS? | | | Last 3-months? | | |
| --- | --- | --- | --- | --- | --- | --- | --- | --- | --- |
|  | Yes | No | Declined | Yes | No | Declined | Yes | No | Declined |
| Acai berry | 🔾 | 🔾 | 🔾 | 🔾 | 🔾 | 🔾 | 🔾 | 🔾 | 🔾 |
| Aloe vera extract | 🔾 | 🔾 | 🔾 | 🔾 | 🔾 | 🔾 | 🔾 | 🔾 | 🔾 |
| Bee pollen | 🔾 | 🔾 | 🔾 | 🔾 | 🔾 | 🔾 | 🔾 | 🔾 | 🔾 |
| Black cohosh | 🔾 | 🔾 | 🔾 | 🔾 | 🔾 | 🔾 | 🔾 | 🔾 | 🔾 |
| Cranberry fruit extract | 🔾 | 🔾 | 🔾 | 🔾 | 🔾 | 🔾 | 🔾 | 🔾 | 🔾 |
| Echinacea | 🔾 | 🔾 | 🔾 | 🔾 | 🔾 | 🔾 | 🔾 | 🔾 | 🔾 |
| Ephedra, Ma Huang | 🔾 | 🔾 | 🔾 | 🔾 | 🔾 | 🔾 | 🔾 | 🔾 | 🔾 |
| Evening primrose | 🔾 | 🔾 | 🔾 | 🔾 | 🔾 | 🔾 | 🔾 | 🔾 | 🔾 |
| Fenugreek | 🔾 | 🔾 | 🔾 | 🔾 | 🔾 | 🔾 | 🔾 | 🔾 | 🔾 |
| Garlic supplement (other than for cooking) | 🔾 | 🔾 | 🔾 | 🔾 | 🔾 | 🔾 | 🔾 | 🔾 | 🔾 |
| Ginger supplement (other than for cooking) | 🔾 | 🔾 | 🔾 | 🔾 | 🔾 | 🔾 | 🔾 | 🔾 | 🔾 |
| Gingko | 🔾 | 🔾 | 🔾 | 🔾 | 🔾 | 🔾 | 🔾 | 🔾 | 🔾 |
| Green tea extract | 🔾 | 🔾 | 🔾 | 🔾 | 🔾 | 🔾 | 🔾 | 🔾 | 🔾 |
| Kava | 🔾 | 🔾 | 🔾 | 🔾 | 🔾 | 🔾 | 🔾 | 🔾 | 🔾 |
| Milk thistle | 🔾 | 🔾 | 🔾 | 🔾 | 🔾 | 🔾 | 🔾 | 🔾 | 🔾 |
| Padma basic | 🔾 | 🔾 | 🔾 | 🔾 | 🔾 | 🔾 | 🔾 | 🔾 | 🔾 |
| Peppermint | 🔾 | 🔾 | 🔾 | 🔾 | 🔾 | 🔾 | 🔾 | 🔾 | 🔾 |
| Red yeast rice | 🔾 | 🔾 | 🔾 | 🔾 | 🔾 | 🔾 | 🔾 | 🔾 | 🔾 |
| Resveratrol | 🔾 | 🔾 | 🔾 | 🔾 | 🔾 | 🔾 | 🔾 | 🔾 | 🔾 |
| Rosehips | 🔾 | 🔾 | 🔾 | 🔾 | 🔾 | 🔾 | 🔾 | 🔾 | 🔾 |
| Saw palmetto | 🔾 | 🔾 | 🔾 | 🔾 | 🔾 | 🔾 | 🔾 | 🔾 | 🔾 |
| Sea kelp | 🔾 | 🔾 | 🔾 | 🔾 | 🔾 | 🔾 | 🔾 | 🔾 | 🔾 |
| Soy estrogens | 🔾 | 🔾 | 🔾 | 🔾 | 🔾 | 🔾 | 🔾 | 🔾 | 🔾 |
| St. Johns Wort | 🔾 | 🔾 | 🔾 | 🔾 | 🔾 | 🔾 | 🔾 | 🔾 | 🔾 |
| Stevia extract | 🔾 | 🔾 | 🔾 | 🔾 | 🔾 | 🔾 | 🔾 | 🔾 | 🔾 |
| Turmeric | 🔾 | 🔾 | 🔾 | 🔾 | 🔾 | 🔾 | 🔾 | 🔾 | 🔾 |
| Winter cherry (Ashwagandha) | 🔾 | 🔾 | 🔾 | 🔾 | 🔾 | 🔾 | 🔾 | 🔾 | 🔾 |
| Yohimbe bark | 🔾 | 🔾 | 🔾 | 🔾 | 🔾 | 🔾 | 🔾 | 🔾 | 🔾 |
| Herbal combinations for appetite control | 🔾 | 🔾 | 🔾 | 🔾 | 🔾 | 🔾 | 🔾 | 🔾 | 🔾 |
| Herbal combinations for energy and fatigue control | 🔾 | 🔾 | 🔾 | 🔾 | 🔾 | 🔾 | 🔾 | 🔾 | 🔾 |
| Herbal combinations for menstrual symptoms | 🔾 | 🔾 | 🔾 | 🔾 | 🔾 | 🔾 | 🔾 | 🔾 | 🔾 |
| Herbal combinations for strength muscle-building | 🔾 | 🔾 | 🔾 | 🔾 | 🔾 | 🔾 | 🔾 | 🔾 | 🔾 |
| Herbal combinations for libido, sexual performance | 🔾 | 🔾 | 🔾 | 🔾 | 🔾 | 🔾 | 🔾 | 🔾 | 🔾 |
| Other | 🔾 | 🔾 | 🔾 | 🔾 | 🔾 | 🔾 | 🔾 | 🔾 | 🔾 |

**100) What are your main reasons for using Herbal Supplements?**

____________________________________________________________

**101) Have you ever used Alternative or Complementary Medicine approaches such as chiropractic, homeopathy, massage, hypnosis etc. for 3 months or more?**

🔾 Yes 🔾 No 🔾 Declined

**102) Complementary and Alternative Medicine**

|  | Non-MS Subject or Before Onset of MS? | | | After Onset of MS? | | | Last 3-months? | | |
| --- | --- | --- | --- | --- | --- | --- | --- | --- | --- |
|  | Yes | No | Declined | Yes | No | Declined | Yes | No | Declined |
| Acupuncture | 🔾 | 🔾 | 🔾 | 🔾 | 🔾 | 🔾 | 🔾 | 🔾 | 🔾 |
| Aromatherapy | 🔾 | 🔾 | 🔾 | 🔾 | 🔾 | 🔾 | 🔾 | 🔾 | 🔾 |
| Ayurveda | 🔾 | 🔾 | 🔾 | 🔾 | 🔾 | 🔾 | 🔾 | 🔾 | 🔾 |
| Chinese herbal medicine | 🔾 | 🔾 | 🔾 | 🔾 | 🔾 | 🔾 | 🔾 | 🔾 | 🔾 |
| Chiropractor | 🔾 | 🔾 | 🔾 | 🔾 | 🔾 | 🔾 | 🔾 | 🔾 | 🔾 |
| Electromagnetic therapy | 🔾 | 🔾 | 🔾 | 🔾 | 🔾 | 🔾 | 🔾 | 🔾 | 🔾 |
| Homeopathy | 🔾 | 🔾 | 🔾 | 🔾 | 🔾 | 🔾 | 🔾 | 🔾 | 🔾 |
| Hypnosis | 🔾 | 🔾 | 🔾 | 🔾 | 🔾 | 🔾 | 🔾 | 🔾 | 🔾 |
| Massage | 🔾 | 🔾 | 🔾 | 🔾 | 🔾 | 🔾 | 🔾 | 🔾 | 🔾 |
| Naturopathy | 🔾 | 🔾 | 🔾 | 🔾 | 🔾 | 🔾 | 🔾 | 🔾 | 🔾 |
| Qi gong, Reiki | 🔾 | 🔾 | 🔾 | 🔾 | 🔾 | 🔾 | 🔾 | 🔾 | 🔾 |
| Therapeutic touch | 🔾 | 🔾 | 🔾 | 🔾 | 🔾 | 🔾 | 🔾 | 🔾 | 🔾 |
| Other | 🔾 | 🔾 | 🔾 | 🔾 | 🔾 | 🔾 | 🔾 | 🔾 | 🔾 |
| Bee Stings | 🔾 | 🔾 | 🔾 | 🔾 | 🔾 | 🔾 | 🔾 | 🔾 | 🔾 |

**103) What are your main reasons for seeking alternative and complementary therapies?**

____________________________________________________________

**104) Do you have any allergies to foods, animals, insects, plant pollens, mold, latex?**

🔾 Yes 🔾 No 🔾 Don't Know 🔾 Declined

**105) Allergies to Foods, Animals, Insects, Plant pollens, Mold, Latex etc.**

|  | Non-MS Subject or Before Onset of MS? | | | After Onset of MS? | | | Last 3-months? | | |
| --- | --- | --- | --- | --- | --- | --- | --- | --- | --- |
|  | Yes | No | Declined | Yes | No | Declined | Yes | No | Declined |
| Eggs | 🔾 | 🔾 | 🔾 | 🔾 | 🔾 | 🔾 | 🔾 | 🔾 | 🔾 |
| Milk | 🔾 | 🔾 | 🔾 | 🔾 | 🔾 | 🔾 | 🔾 | 🔾 | 🔾 |
| Peanuts | 🔾 | 🔾 | 🔾 | 🔾 | 🔾 | 🔾 | 🔾 | 🔾 | 🔾 |
| Tree nuts, e.g., almonds, walnuts, etc. | 🔾 | 🔾 | 🔾 | 🔾 | 🔾 | 🔾 | 🔾 | 🔾 | 🔾 |
| Wheat | 🔾 | 🔾 | 🔾 | 🔾 | 🔾 | 🔾 | 🔾 | 🔾 | 🔾 |
| Soy | 🔾 | 🔾 | 🔾 | 🔾 | 🔾 | 🔾 | 🔾 | 🔾 | 🔾 |
| Fish, e.g., bass, cod, flounder | 🔾 | 🔾 | 🔾 | 🔾 | 🔾 | 🔾 | 🔾 | 🔾 | 🔾 |
| Shellfish, e.g., crab, lobster, shrimp | 🔾 | 🔾 | 🔾 | 🔾 | 🔾 | 🔾 | 🔾 | 🔾 | 🔾 |
| Cat allergy, Fel d1 | 🔾 | 🔾 | 🔾 | 🔾 | 🔾 | 🔾 | 🔾 | 🔾 | 🔾 |
| Fur and dander | 🔾 | 🔾 | 🔾 | 🔾 | 🔾 | 🔾 | 🔾 | 🔾 | 🔾 |
| Wool | 🔾 | 🔾 | 🔾 | 🔾 | 🔾 | 🔾 | 🔾 | 🔾 | 🔾 |
| Bee sting | 🔾 | 🔾 | 🔾 | 🔾 | 🔾 | 🔾 | 🔾 | 🔾 | 🔾 |
| Cockroach | 🔾 | 🔾 | 🔾 | 🔾 | 🔾 | 🔾 | 🔾 | 🔾 | 🔾 |
| Dust mite excretion | 🔾 | 🔾 | 🔾 | 🔾 | 🔾 | 🔾 | 🔾 | 🔾 | 🔾 |
| Mosquito sting | 🔾 | 🔾 | 🔾 | 🔾 | 🔾 | 🔾 | 🔾 | 🔾 | 🔾 |
| Wasp sting | 🔾 | 🔾 | 🔾 | 🔾 | 🔾 | 🔾 | 🔾 | 🔾 | 🔾 |
| Grass, e.g., rye grass | 🔾 | 🔾 | 🔾 | 🔾 | 🔾 | 🔾 | 🔾 | 🔾 | 🔾 |
| Tree, e.g., birch, alder etc. | 🔾 | 🔾 | 🔾 | 🔾 | 🔾 | 🔾 | 🔾 | 🔾 | 🔾 |
| Weeds, e.g., ragweed, nettle | 🔾 | 🔾 | 🔾 | 🔾 | 🔾 | 🔾 | 🔾 | 🔾 | 🔾 |
| Mold spores | 🔾 | 🔾 | 🔾 | 🔾 | 🔾 | 🔾 | 🔾 | 🔾 | 🔾 |
| Latex | 🔾 | 🔾 | 🔾 | 🔾 | 🔾 | 🔾 | 🔾 | 🔾 | 🔾 |
| Metals | 🔾 | 🔾 | 🔾 | 🔾 | 🔾 | 🔾 | 🔾 | 🔾 | 🔾 |
| Solvents | 🔾 | 🔾 | 🔾 | 🔾 | 🔾 | 🔾 | 🔾 | 🔾 | 🔾 |
| Other | 🔾 | 🔾 | 🔾 | 🔾 | 🔾 | 🔾 | 🔾 | 🔾 | 🔾 |

**106) Did you have a fracture or dislocation of the arm, leg, knee, shoulder, skull, vertebra etc.?**

🔾 Yes 🔾 No 🔾 Declined 🔾 Not applicable

**107) Fracture History. Distinguish between traumatic and non-traumatic fractures here. Traumatic fractures are due to accidents, automobile collisions, falls from heights, sports injuries. Non-traumatic fractures are falls from standing height due to loss of balance, weakness etc.**

|  | Non-MS Subject or Before Onset of MS? | | | | After Onset of MS? | | | | Last 3-months? | | | |
| --- | --- | --- | --- | --- | --- | --- | --- | --- | --- | --- | --- | --- |
|  | Yes, Traumatic | Yes, Non-traumatic | No | Declined | Yes, Traumatic | Yes, Non-traumatic | No | Declined | Yes, Traumatic | Yes, Non-traumatic | No | Declined |
| Arm | 🔾 | 🔾 | 🔾 | 🔾 | 🔾 | 🔾 | 🔾 | 🔾 | 🔾 | 🔾 | 🔾 | 🔾 |
| Hand, wrist, finger | 🔾 | 🔾 | 🔾 | 🔾 | 🔾 | 🔾 | 🔾 | 🔾 | 🔾 | 🔾 | 🔾 | 🔾 |
| Hip | 🔾 | 🔾 | 🔾 | 🔾 | 🔾 | 🔾 | 🔾 | 🔾 | 🔾 | 🔾 | 🔾 | 🔾 |
| Leg, knee, foot | 🔾 | 🔾 | 🔾 | 🔾 | 🔾 | 🔾 | 🔾 | 🔾 | 🔾 | 🔾 | 🔾 | 🔾 |
| Rib | 🔾 | 🔾 | 🔾 | 🔾 | 🔾 | 🔾 | 🔾 | 🔾 | 🔾 | 🔾 | 🔾 | 🔾 |
| Shoulder dislocation | 🔾 | 🔾 | 🔾 | 🔾 | 🔾 | 🔾 | 🔾 | 🔾 | 🔾 | 🔾 | 🔾 | 🔾 |
| Skull | 🔾 | 🔾 | 🔾 | 🔾 | 🔾 | 🔾 | 🔾 | 🔾 | 🔾 | 🔾 | 🔾 | 🔾 |
| Vertebra | 🔾 | 🔾 | 🔾 | 🔾 | 🔾 | 🔾 | 🔾 | 🔾 | 🔾 | 🔾 | 🔾 | 🔾 |
| Spine, neck | 🔾 | 🔾 | 🔾 | 🔾 | 🔾 | 🔾 | 🔾 | 🔾 | 🔾 | 🔾 | 🔾 | 🔾 |
| Other | 🔾 | 🔾 | 🔾 | 🔾 | 🔾 | 🔾 | 🔾 | 🔾 | 🔾 | 🔾 | 🔾 | 🔾 |

**108) Comments regarding fracture history.**

____________________________________________________________

**109) Recreational History. Consider the last three months and provide an average estimate in hours per week you  were involved in the following activities.**

|  | None | Less than 1 hour/week | 1 to 2 hours/week | 3 to 5 hours/week | 6 to 9 hours/week | 10 to 12 hours/week | 12 to 18 hours/week | Over 18 hours/week | Declined |
| --- | --- | --- | --- | --- | --- | --- | --- | --- | --- |
| Reading books | 🔾 | 🔾 | 🔾 | 🔾 | 🔾 | 🔾 | 🔾 | 🔾 | 🔾 |
| Newspaper reading | 🔾 | 🔾 | 🔾 | 🔾 | 🔾 | 🔾 | 🔾 | 🔾 | 🔾 |
| Magazine reading | 🔾 | 🔾 | 🔾 | 🔾 | 🔾 | 🔾 | 🔾 | 🔾 | 🔾 |
| Job-related reading | 🔾 | 🔾 | 🔾 | 🔾 | 🔾 | 🔾 | 🔾 | 🔾 | 🔾 |
| Puzzle or crossword solving | 🔾 | 🔾 | 🔾 | 🔾 | 🔾 | 🔾 | 🔾 | 🔾 | 🔾 |
| Video games | 🔾 | 🔾 | 🔾 | 🔾 | 🔾 | 🔾 | 🔾 | 🔾 | 🔾 |
| Movies (Theater or video) | 🔾 | 🔾 | 🔾 | 🔾 | 🔾 | 🔾 | 🔾 | 🔾 | 🔾 |
| Television | 🔾 | 🔾 | 🔾 | 🔾 | 🔾 | 🔾 | 🔾 | 🔾 | 🔾 |
| Internet browsing | 🔾 | 🔾 | 🔾 | 🔾 | 🔾 | 🔾 | 🔾 | 🔾 | 🔾 |
| Social network sites (e.g., Facebook, SecondLife, etc.) | 🔾 | 🔾 | 🔾 | 🔾 | 🔾 | 🔾 | 🔾 | 🔾 | 🔾 |
| Email | 🔾 | 🔾 | 🔾 | 🔾 | 🔾 | 🔾 | 🔾 | 🔾 | 🔾 |
| Poker, Bridge, Cards, Board games, Chess | 🔾 | 🔾 | 🔾 | 🔾 | 🔾 | 🔾 | 🔾 | 🔾 | 🔾 |
| Prayer | 🔾 | 🔾 | 🔾 | 🔾 | 🔾 | 🔾 | 🔾 | 🔾 | 🔾 |
| Meditation or other spiritual activity | 🔾 | 🔾 | 🔾 | 🔾 | 🔾 | 🔾 | 🔾 | 🔾 | 🔾 |

**110) What is the color of your eyes?**

🔾 Light blue, light gray, light green: Level 0 🔾 Dark brown 🔾 Other

🔾 Blue, gray or green: Level 1 🔾 Black 🔾 Declined

🔾 Dark blue or hazel: Level 2

**111) What is or was the color of the Natural Color of your Hair. If you are graying or have hair loss, indicate the Color before you turned gray or before you lost your hair.**

🔾 Sandy or Red: Level 0 🔾 Dark brown 🔾 Other

🔾 Blonde: Level 1 🔾 Black 🔾 Declined

🔾 Chestnut or Dark Blonde: Level 2

**112) What is the color of the Non-Exposed areas of Skin?**

🔾 Very Light or "Nordic", Tends to have freckles, red or blond hair, blue or green eyes, Type 1

🔾 Light Skinned European, Tends to have light hair, blue/green or brown eyes, Type 2

🔾 Light Intermediate skin, Dark-skinned European or "Average Caucasian", Tends to have brown hair and eyes, Type 3

🔾 Dark-Intermediate skin, "Mediterranean" or "Olive", Tends to have dark brown hair and eyes, Type 4

🔾 Dark or Brown skin, Naturally Black-brown skin, Dark brown eyes and hair, Type 5

🔾 Very Dark skin, Naturally Black-brown skin, , Black-brown eyes and hair, Type 6

🔾 Other

🔾 Declined

**113) Do you have freckles on unexposed area of skin?**

🔾 Many: Level 0 🔾 Incidental 🔾 Other

🔾 Several: Level 1 🔾 None 🔾 Declined

🔾 Few: Level 2

**114) What happens when you stay in the sun too long?**

🔾 Painful redness, blistering, peeling: Level 0 🔾 Never had burns: Level 4

🔾 Blistering followed by peeling: Level 1 🔾 Don't know.

🔾 Burns sometimes followed by peeling: Level 2 🔾 Declined

🔾 Rarely burns: Level 3

**115) To what degree do you turn brown?**

🔾 Hardly or not at all: Level 0 🔾 Turns dark brown quickly: Level 4

🔾 Light color tan: Level 1 🔾 Don't know.

🔾 Reasonable tan: Level 2 🔾 Declined

🔾 Tans easily: Level 3

**116) Do you turn brown within several hours of sun exposure?**

🔾 Never: Level 0 🔾 Often: Level 3 🔾 Don't know.

🔾 Seldom: Level 1 🔾 Always: Level 4 🔾 Declined

🔾 Sometimes: Level 2

**117) How does your face react to sun exposure?**

🔾 Very sensitive: Level 0 🔾 Very resistant: Level 3 🔾 Don't know.

🔾 Seldom: Level 1 🔾 Never had a problem: Level 4 🔾 Declined

🔾 Normal: Level 2

**118) Sun exposure in Childhood and teenage years until age 18 years. Provide your answers considering the entire period. If you had MS prior to age 18, provide the answers for the period until onset.**

|  | Childhood Summer Exposure, Days Per Summer | | | | | | | | | | |
| --- | --- | --- | --- | --- | --- | --- | --- | --- | --- | --- | --- |
|  | None | 1 day | 2 to 3 days | 4 to 6 days | 1 Week | 2-3 Weeks | 4 Weeks or 1 Month | 2 Months | 3 Months | 3-6 Months | Not known |
| Job-related sun exposure, e.g., Lifeguard | 🔾 | 🔾 | 🔾 | 🔾 | 🔾 | 🔾 | 🔾 | 🔾 | 🔾 | 🔾 | 🔾 |
| Exercise related sun exposure, e.g., Skiing, cycling | 🔾 | 🔾 | 🔾 | 🔾 | 🔾 | 🔾 | 🔾 | 🔾 | 🔾 | 🔾 | 🔾 |
| Leisure related sun exposure, e.g., Mowing, gardening | 🔾 | 🔾 | 🔾 | 🔾 | 🔾 | 🔾 | 🔾 | 🔾 | 🔾 | 🔾 | 🔾 |
| Beach, vacation related exposure | 🔾 | 🔾 | 🔾 | 🔾 | 🔾 | 🔾 | 🔾 | 🔾 | 🔾 | 🔾 | 🔾 |
| Sun bathing | 🔾 | 🔾 | 🔾 | 🔾 | 🔾 | 🔾 | 🔾 | 🔾 | 🔾 | 🔾 | 🔾 |
| Tanning bed | 🔾 | 🔾 | 🔾 | 🔾 | 🔾 | 🔾 | 🔾 | 🔾 | 🔾 | 🔾 | 🔾 |
| Other | 🔾 | 🔾 | 🔾 | 🔾 | 🔾 | 🔾 | 🔾 | 🔾 | 🔾 | 🔾 | 🔾 |

|  | Childhood Winter Exposure, Days Per Winter | | | | | | | | | | |
| --- | --- | --- | --- | --- | --- | --- | --- | --- | --- | --- | --- |
|  | None | 1 day | 2 to 3 days | 4 to 6 days | 1 Week | 2-3 Weeks | 4 Weeks or 1 Month | 2 Months | 3 Months | 3-6 Months | Not known |
| Job-related sun exposure, e.g., Lifeguard | 🔾 | 🔾 | 🔾 | 🔾 | 🔾 | 🔾 | 🔾 | 🔾 | 🔾 | 🔾 | 🔾 |
| Exercise related sun exposure, e.g., Skiing, cycling | 🔾 | 🔾 | 🔾 | 🔾 | 🔾 | 🔾 | 🔾 | 🔾 | 🔾 | 🔾 | 🔾 |
| Leisure related sun exposure, e.g., Mowing, gardening | 🔾 | 🔾 | 🔾 | 🔾 | 🔾 | 🔾 | 🔾 | 🔾 | 🔾 | 🔾 | 🔾 |
| Beach, vacation related exposure | 🔾 | 🔾 | 🔾 | 🔾 | 🔾 | 🔾 | 🔾 | 🔾 | 🔾 | 🔾 | 🔾 |
| Sun bathing | 🔾 | 🔾 | 🔾 | 🔾 | 🔾 | 🔾 | 🔾 | 🔾 | 🔾 | 🔾 | 🔾 |
| Tanning bed | 🔾 | 🔾 | 🔾 | 🔾 | 🔾 | 🔾 | 🔾 | 🔾 | 🔾 | 🔾 | 🔾 |
| Other | 🔾 | 🔾 | 🔾 | 🔾 | 🔾 | 🔾 | 🔾 | 🔾 | 🔾 | 🔾 | 🔾 |

**119) Comments on Childhood Sun Exposure.**

____________________________________________________________

**120) Sun exposure in the Last Two Years. Provide your answers considering the entire period.**

|  | Last 2-Years Summer Exposure, Days Per summer | | | | | | | | | | |
| --- | --- | --- | --- | --- | --- | --- | --- | --- | --- | --- | --- |
|  | None | 1 day | 2 to 3 days | 4 to 6 days | 1 Week | 2-3 Weeks | 4 Weeks or 1 Month | 2 Months | 3 Months | 3-6 Months | Not known |
| Job-related sun exposure, e.g., Lifeguard | 🔾 | 🔾 | 🔾 | 🔾 | 🔾 | 🔾 | 🔾 | 🔾 | 🔾 | 🔾 | 🔾 |
| Exercise related sun exposure, e.g., Skiing, cycling | 🔾 | 🔾 | 🔾 | 🔾 | 🔾 | 🔾 | 🔾 | 🔾 | 🔾 | 🔾 | 🔾 |
| Leisure related sun exposure, e.g., Mowing, gardening | 🔾 | 🔾 | 🔾 | 🔾 | 🔾 | 🔾 | 🔾 | 🔾 | 🔾 | 🔾 | 🔾 |
| Beach, vacation related exposure | 🔾 | 🔾 | 🔾 | 🔾 | 🔾 | 🔾 | 🔾 | 🔾 | 🔾 | 🔾 | 🔾 |
| Sun bathing | 🔾 | 🔾 | 🔾 | 🔾 | 🔾 | 🔾 | 🔾 | 🔾 | 🔾 | 🔾 | 🔾 |
| Tanning bed | 🔾 | 🔾 | 🔾 | 🔾 | 🔾 | 🔾 | 🔾 | 🔾 | 🔾 | 🔾 | 🔾 |
| Other | 🔾 | 🔾 | 🔾 | 🔾 | 🔾 | 🔾 | 🔾 | 🔾 | 🔾 | 🔾 | 🔾 |

|  | Last 2-Years Winter Exposure, Days Per winter | | | | | | | | | | |
| --- | --- | --- | --- | --- | --- | --- | --- | --- | --- | --- | --- |
|  | None | 1 day | 2 to 3 days | 4 to 6 days | 1 Week | 2-3 Weeks | 4 Weeks or 1 Month | 2 Months | 3 Months | 3-6 Months | Not known |
| Job-related sun exposure, e.g., Lifeguard | 🔾 | 🔾 | 🔾 | 🔾 | 🔾 | 🔾 | 🔾 | 🔾 | 🔾 | 🔾 | 🔾 |
| Exercise related sun exposure, e.g., Skiing, cycling | 🔾 | 🔾 | 🔾 | 🔾 | 🔾 | 🔾 | 🔾 | 🔾 | 🔾 | 🔾 | 🔾 |
| Leisure related sun exposure, e.g., Mowing, gardening | 🔾 | 🔾 | 🔾 | 🔾 | 🔾 | 🔾 | 🔾 | 🔾 | 🔾 | 🔾 | 🔾 |
| Beach, vacation related exposure | 🔾 | 🔾 | 🔾 | 🔾 | 🔾 | 🔾 | 🔾 | 🔾 | 🔾 | 🔾 | 🔾 |
| Sun bathing | 🔾 | 🔾 | 🔾 | 🔾 | 🔾 | 🔾 | 🔾 | 🔾 | 🔾 | 🔾 | 🔾 |
| Tanning bed | 🔾 | 🔾 | 🔾 | 🔾 | 🔾 | 🔾 | 🔾 | 🔾 | 🔾 | 🔾 | 🔾 |
| Other | 🔾 | 🔾 | 🔾 | 🔾 | 🔾 | 🔾 | 🔾 | 🔾 | 🔾 | 🔾 | 🔾 |

**121) Comments on Sun Exposure in Last Two Years.**

____________________________________________________________

**122) Vaccinations**

|  | Vaccination Status | | | | |
| --- | --- | --- | --- | --- | --- |
|  | Yes | Yes but not complete course | No | Don't know | Not applicable |
| Tetanus-Diphtheria-Pertussis (TDP) vaccine | 🔾 | 🔾 | 🔾 | 🔾 | 🔾 |
| Hepatitis A vaccine | 🔾 | 🔾 | 🔾 | 🔾 | 🔾 |
| Hepatitis B vaccine | 🔾 | 🔾 | 🔾 | 🔾 | 🔾 |
| HiB (H. influenzae) vaccine | 🔾 | 🔾 | 🔾 | 🔾 | 🔾 |
| Influenza vaccine | 🔾 | 🔾 | 🔾 | 🔾 | 🔾 |
| Measles-Mumps-Rubella (MMR vaccine) | 🔾 | 🔾 | 🔾 | 🔾 | 🔾 |
| Polio (inactivated vaccine) | 🔾 | 🔾 | 🔾 | 🔾 | 🔾 |
| Pneumococcal vaccine | 🔾 | 🔾 | 🔾 | 🔾 | 🔾 |
| Rotavirus vaccine | 🔾 | 🔾 | 🔾 | 🔾 | 🔾 |
| Varicella (chicken pox) vaccine | 🔾 | 🔾 | 🔾 | 🔾 | 🔾 |
| TDP vaccine | 🔾 | 🔾 | 🔾 | 🔾 | 🔾 |
| Human papillomavirus vaccine (for girls, mark not applicable for males) | 🔾 | 🔾 | 🔾 | 🔾 | 🔾 |
| Meningococcus vaccine | 🔾 | 🔾 | 🔾 | 🔾 | 🔾 |
| Tuberculosis vaccine | 🔾 | 🔾 | 🔾 | 🔾 | 🔾 |
| Other | 🔾 | 🔾 | 🔾 | 🔾 | 🔾 |

**123) Occurrence of Infections**

|  | Occurrence of Disease | | | | |
| --- | --- | --- | --- | --- | --- |
|  | No | Yes | Don't know | Not applicable | Declined |
| Mononucleosis | 🔾 | 🔾 | 🔾 | 🔾 | 🔾 |
| Hepatitis A | 🔾 | 🔾 | 🔾 | 🔾 | 🔾 |
| Hepatitis B | 🔾 | 🔾 | 🔾 | 🔾 | 🔾 |
| Hepatitis C | 🔾 | 🔾 | 🔾 | 🔾 | 🔾 |
| Hepatitis Type unknown | 🔾 | 🔾 | 🔾 | 🔾 | 🔾 |
| Measles | 🔾 | 🔾 | 🔾 | 🔾 | 🔾 |
| Mumps | 🔾 | 🔾 | 🔾 | 🔾 | 🔾 |
| German measles or Rubella | 🔾 | 🔾 | 🔾 | 🔾 | 🔾 |
| Chicken pox | 🔾 | 🔾 | 🔾 | 🔾 | 🔾 |
| Whooping cough or Pertussis | 🔾 | 🔾 | 🔾 | 🔾 | 🔾 |
| Tuberculosis | 🔾 | 🔾 | 🔾 | 🔾 | 🔾 |
| Human papillomavirus vaccine (for girls, mark N/A for males) | 🔾 | 🔾 | 🔾 | 🔾 | 🔾 |
| Meningococcus vaccine | 🔾 | 🔾 | 🔾 | 🔾 | 🔾 |
| Tuberculosis vaccine | 🔾 | 🔾 | 🔾 | 🔾 | 🔾 |
| Other | 🔾 | 🔾 | 🔾 | 🔾 | 🔾 |

**124) Comments Regarding Occurrence of Infections**

____________________________________________________________

**125) Have you ever had any Parasitic Infections such as Head or Body Louse, Scabies, Tapeworm, Malaria etc.?**

🔾 Yes 🔾 No 🔾 Don't know 🔾 Don't Remember 🔾 Declined

**126) Occurrence of Parasitic Infections. Parasitic infections include head or body louse, tapeworm, malaria, scabies etc.**

|  | Non-MS Subject or Before Onset of MS | | | | | |
| --- | --- | --- | --- | --- | --- | --- |
|  | No | Yes | Don't remember | Don't know | Not applicable | Declined |
| Amoebiasis: Parasitic Worms | 🔾 | 🔾 | 🔾 | 🔾 | 🔾 | 🔾 |
| Coccidia: Parasitic Worms | 🔾 | 🔾 | 🔾 | 🔾 | 🔾 | 🔾 |
| Giardia: Parasitic Worms | 🔾 | 🔾 | 🔾 | 🔾 | 🔾 | 🔾 |
| Guinea worm: Parasitic Worms | 🔾 | 🔾 | 🔾 | 🔾 | 🔾 | 🔾 |
| Head, body, or crab louse: Parasitic Worms | 🔾 | 🔾 | 🔾 | 🔾 | 🔾 | 🔾 |
| Liver fluke: Parasitic Worms | 🔾 | 🔾 | 🔾 | 🔾 | 🔾 | 🔾 |
| Pinworm: Parasitic Worms | 🔾 | 🔾 | 🔾 | 🔾 | 🔾 | 🔾 |
| Roundworm: Parasitic Worms | 🔾 | 🔾 | 🔾 | 🔾 | 🔾 | 🔾 |
| Scabies: Parasitic Worms | 🔾 | 🔾 | 🔾 | 🔾 | 🔾 | 🔾 |
| Strongyloidiasis: Parasitic Worms | 🔾 | 🔾 | 🔾 | 🔾 | 🔾 | 🔾 |
| Tapeworm: Parasitic Worms | 🔾 | 🔾 | 🔾 | 🔾 | 🔾 | 🔾 |
| Toxocariasis: Parasitic Worms | 🔾 | 🔾 | 🔾 | 🔾 | 🔾 | 🔾 |
| Toxoplasmosis: Parasitic Worms | 🔾 | 🔾 | 🔾 | 🔾 | 🔾 | 🔾 |
| Trichinosis: Parasitic Worms | 🔾 | 🔾 | 🔾 | 🔾 | 🔾 | 🔾 |
| Whipworm: Parasitic Worms | 🔾 | 🔾 | 🔾 | 🔾 | 🔾 | 🔾 |
| Type Not Known: Parasitic Worms | 🔾 | 🔾 | 🔾 | 🔾 | 🔾 | 🔾 |
| African trypanosomiasis | 🔾 | 🔾 | 🔾 | 🔾 | 🔾 | 🔾 |
| Chagas disease: Tropical parasite | 🔾 | 🔾 | 🔾 | 🔾 | 🔾 | 🔾 |
| Dengue fever: Tropical parasite | 🔾 | 🔾 | 🔾 | 🔾 | 🔾 | 🔾 |
| Leishmaniasis: Tropical parasite | 🔾 | 🔾 | 🔾 | 🔾 | 🔾 | 🔾 |
| Lymphatic filariasis: Tropical parasite | 🔾 | 🔾 | 🔾 | 🔾 | 🔾 | 🔾 |
| Malaria: Tropical parasite | 🔾 | 🔾 | 🔾 | 🔾 | 🔾 | 🔾 |
| Onchocerciasis: Tropical parasite | 🔾 | 🔾 | 🔾 | 🔾 | 🔾 | 🔾 |
| Schistosomiasis: Tropical parasite | 🔾 | 🔾 | 🔾 | 🔾 | 🔾 | 🔾 |
| Trachoma: Tropical parasite | 🔾 | 🔾 | 🔾 | 🔾 | 🔾 | 🔾 |
| Type Not Known: Tropical parasite | 🔾 | 🔾 | 🔾 | 🔾 | 🔾 | 🔾 |
| Other (Specify) | 🔾 | 🔾 | 🔾 | 🔾 | 🔾 | 🔾 |

|  | After Onset of MS | | | | | |
| --- | --- | --- | --- | --- | --- | --- |
|  | No | Yes | Don't remember | Don't know | Not applicable | Declined |
| Amoebiasis: Parasitic Worms | 🔾 | 🔾 | 🔾 | 🔾 | 🔾 | 🔾 |
| Coccidia: Parasitic Worms | 🔾 | 🔾 | 🔾 | 🔾 | 🔾 | 🔾 |
| Giardia: Parasitic Worms | 🔾 | 🔾 | 🔾 | 🔾 | 🔾 | 🔾 |
| Guinea worm: Parasitic Worms | 🔾 | 🔾 | 🔾 | 🔾 | 🔾 | 🔾 |
| Head, body, or crab louse: Parasitic Worms | 🔾 | 🔾 | 🔾 | 🔾 | 🔾 | 🔾 |
| Liver fluke: Parasitic Worms | 🔾 | 🔾 | 🔾 | 🔾 | 🔾 | 🔾 |
| Pinworm: Parasitic Worms | 🔾 | 🔾 | 🔾 | 🔾 | 🔾 | 🔾 |
| Roundworm: Parasitic Worms | 🔾 | 🔾 | 🔾 | 🔾 | 🔾 | 🔾 |
| Scabies: Parasitic Worms | 🔾 | 🔾 | 🔾 | 🔾 | 🔾 | 🔾 |
| Strongyloidiasis: Parasitic Worms | 🔾 | 🔾 | 🔾 | 🔾 | 🔾 | 🔾 |
| Tapeworm: Parasitic Worms | 🔾 | 🔾 | 🔾 | 🔾 | 🔾 | 🔾 |
| Toxocariasis: Parasitic Worms | 🔾 | 🔾 | 🔾 | 🔾 | 🔾 | 🔾 |
| Toxoplasmosis: Parasitic Worms | 🔾 | 🔾 | 🔾 | 🔾 | 🔾 | 🔾 |
| Trichinosis: Parasitic Worms | 🔾 | 🔾 | 🔾 | 🔾 | 🔾 | 🔾 |
| Whipworm: Parasitic Worms | 🔾 | 🔾 | 🔾 | 🔾 | 🔾 | 🔾 |
| Type Not Known: Parasitic Worms | 🔾 | 🔾 | 🔾 | 🔾 | 🔾 | 🔾 |
| African trypanosomiasis | 🔾 | 🔾 | 🔾 | 🔾 | 🔾 | 🔾 |
| Chagas disease: Tropical parasite | 🔾 | 🔾 | 🔾 | 🔾 | 🔾 | 🔾 |
| Dengue fever: Tropical parasite | 🔾 | 🔾 | 🔾 | 🔾 | 🔾 | 🔾 |
| Leishmaniasis: Tropical parasite | 🔾 | 🔾 | 🔾 | 🔾 | 🔾 | 🔾 |
| Lymphatic filariasis: Tropical parasite | 🔾 | 🔾 | 🔾 | 🔾 | 🔾 | 🔾 |
| Malaria: Tropical parasite | 🔾 | 🔾 | 🔾 | 🔾 | 🔾 | 🔾 |
| Onchocerciasis: Tropical parasite | 🔾 | 🔾 | 🔾 | 🔾 | 🔾 | 🔾 |
| Schistosomiasis: Tropical parasite | 🔾 | 🔾 | 🔾 | 🔾 | 🔾 | 🔾 |
| Trachoma: Tropical parasite | 🔾 | 🔾 | 🔾 | 🔾 | 🔾 | 🔾 |
| Type Not Known: Tropical parasite | 🔾 | 🔾 | 🔾 | 🔾 | 🔾 | 🔾 |
| Other (Specify) | 🔾 | 🔾 | 🔾 | 🔾 | 🔾 | 🔾 |

|  | Last 3-Months? | | | | | |
| --- | --- | --- | --- | --- | --- | --- |
|  | No | Yes | Don't remember | Don't know | Not applicable | Declined |
| Amoebiasis: Parasitic Worms | 🔾 | 🔾 | 🔾 | 🔾 | 🔾 | 🔾 |
| Coccidia: Parasitic Worms | 🔾 | 🔾 | 🔾 | 🔾 | 🔾 | 🔾 |
| Giardia: Parasitic Worms | 🔾 | 🔾 | 🔾 | 🔾 | 🔾 | 🔾 |
| Guinea worm: Parasitic Worms | 🔾 | 🔾 | 🔾 | 🔾 | 🔾 | 🔾 |
| Head, body, or crab louse: Parasitic Worms | 🔾 | 🔾 | 🔾 | 🔾 | 🔾 | 🔾 |
| Liver fluke: Parasitic Worms | 🔾 | 🔾 | 🔾 | 🔾 | 🔾 | 🔾 |
| Pinworm: Parasitic Worms | 🔾 | 🔾 | 🔾 | 🔾 | 🔾 | 🔾 |
| Roundworm: Parasitic Worms | 🔾 | 🔾 | 🔾 | 🔾 | 🔾 | 🔾 |
| Scabies: Parasitic Worms | 🔾 | 🔾 | 🔾 | 🔾 | 🔾 | 🔾 |
| Strongyloidiasis: Parasitic Worms | 🔾 | 🔾 | 🔾 | 🔾 | 🔾 | 🔾 |
| Tapeworm: Parasitic Worms | 🔾 | 🔾 | 🔾 | 🔾 | 🔾 | 🔾 |
| Toxocariasis: Parasitic Worms | 🔾 | 🔾 | 🔾 | 🔾 | 🔾 | 🔾 |
| Toxoplasmosis: Parasitic Worms | 🔾 | 🔾 | 🔾 | 🔾 | 🔾 | 🔾 |
| Trichinosis: Parasitic Worms | 🔾 | 🔾 | 🔾 | 🔾 | 🔾 | 🔾 |
| Whipworm: Parasitic Worms | 🔾 | 🔾 | 🔾 | 🔾 | 🔾 | 🔾 |
| Type Not Known: Parasitic Worms | 🔾 | 🔾 | 🔾 | 🔾 | 🔾 | 🔾 |
| African trypanosomiasis | 🔾 | 🔾 | 🔾 | 🔾 | 🔾 | 🔾 |
| Chagas disease: Tropical parasite | 🔾 | 🔾 | 🔾 | 🔾 | 🔾 | 🔾 |
| Dengue fever: Tropical parasite | 🔾 | 🔾 | 🔾 | 🔾 | 🔾 | 🔾 |
| Leishmaniasis: Tropical parasite | 🔾 | 🔾 | 🔾 | 🔾 | 🔾 | 🔾 |
| Lymphatic filariasis: Tropical parasite | 🔾 | 🔾 | 🔾 | 🔾 | 🔾 | 🔾 |
| Malaria: Tropical parasite | 🔾 | 🔾 | 🔾 | 🔾 | 🔾 | 🔾 |
| Onchocerciasis: Tropical parasite | 🔾 | 🔾 | 🔾 | 🔾 | 🔾 | 🔾 |
| Schistosomiasis: Tropical parasite | 🔾 | 🔾 | 🔾 | 🔾 | 🔾 | 🔾 |
| Trachoma: Tropical parasite | 🔾 | 🔾 | 🔾 | 🔾 | 🔾 | 🔾 |
| Type Not Known: Tropical parasite | 🔾 | 🔾 | 🔾 | 🔾 | 🔾 | 🔾 |
| Other (Specify) | 🔾 | 🔾 | 🔾 | 🔾 | 🔾 | 🔾 |

**127) Comments regarding Parasitic Infections**

____________________________________________________________

**128) Have you ever had surgery (not dental surgery), implants, transfusions, piercings or tattoos?**

🔾 Yes 🔾 No 🔾 Declined

**129) Surgeries, Implants, Transplants, Piercings, Tattoos.**

|  | Non-MS Subject or Before Onset of MS? | | | After Onset of MS? | | | Last 3-months? | | |
| --- | --- | --- | --- | --- | --- | --- | --- | --- | --- |
|  | Yes | No | Declined | Yes | No | Declined | Yes | No | Declined |
| Transfusions | 🔾 | 🔾 | 🔾 | 🔾 | 🔾 | 🔾 | 🔾 | 🔾 | 🔾 |
| Bone marrow transplant | 🔾 | 🔾 | 🔾 | 🔾 | 🔾 | 🔾 | 🔾 | 🔾 | 🔾 |
| Heart transplant | 🔾 | 🔾 | 🔾 | 🔾 | 🔾 | 🔾 | 🔾 | 🔾 | 🔾 |
| Kidney transplant | 🔾 | 🔾 | 🔾 | 🔾 | 🔾 | 🔾 | 🔾 | 🔾 | 🔾 |
| Liver transplant | 🔾 | 🔾 | 🔾 | 🔾 | 🔾 | 🔾 | 🔾 | 🔾 | 🔾 |
| Lung transplant | 🔾 | 🔾 | 🔾 | 🔾 | 🔾 | 🔾 | 🔾 | 🔾 | 🔾 |
| Hair implant | 🔾 | 🔾 | 🔾 | 🔾 | 🔾 | 🔾 | 🔾 | 🔾 | 🔾 |
| Breast implant | 🔾 | 🔾 | 🔾 | 🔾 | 🔾 | 🔾 | 🔾 | 🔾 | 🔾 |
| Cosmetic surgery | 🔾 | 🔾 | 🔾 | 🔾 | 🔾 | 🔾 | 🔾 | 🔾 | 🔾 |
| Gastric band | 🔾 | 🔾 | 🔾 | 🔾 | 🔾 | 🔾 | 🔾 | 🔾 | 🔾 |
| Gastric bypass | 🔾 | 🔾 | 🔾 | 🔾 | 🔾 | 🔾 | 🔾 | 🔾 | 🔾 |
| Lumpectomy | 🔾 | 🔾 | 🔾 | 🔾 | 🔾 | 🔾 | 🔾 | 🔾 | 🔾 |
| Mastectomy | 🔾 | 🔾 | 🔾 | 🔾 | 🔾 | 🔾 | 🔾 | 🔾 | 🔾 |
| Bee Stings | 🔾 | 🔾 | 🔾 | 🔾 | 🔾 | 🔾 | 🔾 | 🔾 | 🔾 |
| Breast reduction | 🔾 | 🔾 | 🔾 | 🔾 | 🔾 | 🔾 | 🔾 | 🔾 | 🔾 |
| Tonsillectomy | 🔾 | 🔾 | 🔾 | 🔾 | 🔾 | 🔾 | 🔾 | 🔾 | 🔾 |
| Hysterectomy (Uterus removed) | 🔾 | 🔾 | 🔾 | 🔾 | 🔾 | 🔾 | 🔾 | 🔾 | 🔾 |
| Ovariectomy (Ovaries removed) | 🔾 | 🔾 | 🔾 | 🔾 | 🔾 | 🔾 | 🔾 | 🔾 | 🔾 |
| Hip replacement | 🔾 | 🔾 | 🔾 | 🔾 | 🔾 | 🔾 | 🔾 | 🔾 | 🔾 |
| Knee replacement | 🔾 | 🔾 | 🔾 | 🔾 | 🔾 | 🔾 | 🔾 | 🔾 | 🔾 |
| Ostomy | 🔾 | 🔾 | 🔾 | 🔾 | 🔾 | 🔾 | 🔾 | 🔾 | 🔾 |
| Pacemaker, defibrillator, cardiac devices | 🔾 | 🔾 | 🔾 | 🔾 | 🔾 | 🔾 | 🔾 | 🔾 | 🔾 |
| Insulin pump | 🔾 | 🔾 | 🔾 | 🔾 | 🔾 | 🔾 | 🔾 | 🔾 | 🔾 |
| Contraceptive implant | 🔾 | 🔾 | 🔾 | 🔾 | 🔾 | 🔾 | 🔾 | 🔾 | 🔾 |
| Vasectomy | 🔾 | 🔾 | 🔾 | 🔾 | 🔾 | 🔾 | 🔾 | 🔾 | 🔾 |
| Tubal ligation | 🔾 | 🔾 | 🔾 | 🔾 | 🔾 | 🔾 | 🔾 | 🔾 | 🔾 |
| Skin graft | 🔾 | 🔾 | 🔾 | 🔾 | 🔾 | 🔾 | 🔾 | 🔾 | 🔾 |
| Tattoos, Body Art | 🔾 | 🔾 | 🔾 | 🔾 | 🔾 | 🔾 | 🔾 | 🔾 | 🔾 |
| More than three piercings (Other than ears) | 🔾 | 🔾 | 🔾 | 🔾 | 🔾 | 🔾 | 🔾 | 🔾 | 🔾 |
| Other | 🔾 | 🔾 | 🔾 | 🔾 | 🔾 | 🔾 | 🔾 | 🔾 | 🔾 |

**130) Did you live with or have Contact with Pet Animals for Three months or more?**

🔾 Yes 🔾 No 🔾 Don't Know 🔾 Declined

**131) Type of Pet Animal**

|  | Non-MS Subject or Before Onset of MS | | | | | | | | | | | | | | | |
| --- | --- | --- | --- | --- | --- | --- | --- | --- | --- | --- | --- | --- | --- | --- | --- | --- |
|  | Dogs | Cats | Birds | Fish | Mice | Rats | Hamsters | Guinea Pigs | Rabbits | Lizards | Snakes or other Reptiles | Primates, monkeys, apes | Don't remember pet type | Other | Not applicable at this time | Declined |
| Pet 1 | 🔾 | 🔾 | 🔾 | 🔾 | 🔾 | 🔾 | 🔾 | 🔾 | 🔾 | 🔾 | 🔾 | 🔾 | 🔾 | 🔾 | 🔾 | 🔾 |
| Pet 2 | 🔾 | 🔾 | 🔾 | 🔾 | 🔾 | 🔾 | 🔾 | 🔾 | 🔾 | 🔾 | 🔾 | 🔾 | 🔾 | 🔾 | 🔾 | 🔾 |
| Pet 3 | 🔾 | 🔾 | 🔾 | 🔾 | 🔾 | 🔾 | 🔾 | 🔾 | 🔾 | 🔾 | 🔾 | 🔾 | 🔾 | 🔾 | 🔾 | 🔾 |
| Pet 4 | 🔾 | 🔾 | 🔾 | 🔾 | 🔾 | 🔾 | 🔾 | 🔾 | 🔾 | 🔾 | 🔾 | 🔾 | 🔾 | 🔾 | 🔾 | 🔾 |
| Pet 5 | 🔾 | 🔾 | 🔾 | 🔾 | 🔾 | 🔾 | 🔾 | 🔾 | 🔾 | 🔾 | 🔾 | 🔾 | 🔾 | 🔾 | 🔾 | 🔾 |
| Pet 6 | 🔾 | 🔾 | 🔾 | 🔾 | 🔾 | 🔾 | 🔾 | 🔾 | 🔾 | 🔾 | 🔾 | 🔾 | 🔾 | 🔾 | 🔾 | 🔾 |
| Pet 7 | 🔾 | 🔾 | 🔾 | 🔾 | 🔾 | 🔾 | 🔾 | 🔾 | 🔾 | 🔾 | 🔾 | 🔾 | 🔾 | 🔾 | 🔾 | 🔾 |

|  | After Onset of MS | | | | | | | | | | | | | | | |
| --- | --- | --- | --- | --- | --- | --- | --- | --- | --- | --- | --- | --- | --- | --- | --- | --- |
|  | Dogs | Cats | Birds | Fish | Mice | Rats | Hamsters | Guinea Pigs | Rabbits | Lizards | Snakes or other Reptiles | Primates, monkeys, apes | Don't remember pet type | Other | Not applicable at this time | Declined |
| Pet 1 | 🔾 | 🔾 | 🔾 | 🔾 | 🔾 | 🔾 | 🔾 | 🔾 | 🔾 | 🔾 | 🔾 | 🔾 | 🔾 | 🔾 | 🔾 | 🔾 |
| Pet 2 | 🔾 | 🔾 | 🔾 | 🔾 | 🔾 | 🔾 | 🔾 | 🔾 | 🔾 | 🔾 | 🔾 | 🔾 | 🔾 | 🔾 | 🔾 | 🔾 |
| Pet 3 | 🔾 | 🔾 | 🔾 | 🔾 | 🔾 | 🔾 | 🔾 | 🔾 | 🔾 | 🔾 | 🔾 | 🔾 | 🔾 | 🔾 | 🔾 | 🔾 |
| Pet 4 | 🔾 | 🔾 | 🔾 | 🔾 | 🔾 | 🔾 | 🔾 | 🔾 | 🔾 | 🔾 | 🔾 | 🔾 | 🔾 | 🔾 | 🔾 | 🔾 |
| Pet 5 | 🔾 | 🔾 | 🔾 | 🔾 | 🔾 | 🔾 | 🔾 | 🔾 | 🔾 | 🔾 | 🔾 | 🔾 | 🔾 | 🔾 | 🔾 | 🔾 |
| Pet 6 | 🔾 | 🔾 | 🔾 | 🔾 | 🔾 | 🔾 | 🔾 | 🔾 | 🔾 | 🔾 | 🔾 | 🔾 | 🔾 | 🔾 | 🔾 | 🔾 |
| Pet 7 | 🔾 | 🔾 | 🔾 | 🔾 | 🔾 | 🔾 | 🔾 | 🔾 | 🔾 | 🔾 | 🔾 | 🔾 | 🔾 | 🔾 | 🔾 | 🔾 |

|  | Last 3-Months | | | | | | | | | | | | | | | |
| --- | --- | --- | --- | --- | --- | --- | --- | --- | --- | --- | --- | --- | --- | --- | --- | --- |
|  | Dogs | Cats | Birds | Fish | Mice | Rats | Hamsters | Guinea Pigs | Rabbits | Lizards | Snakes or other Reptiles | Primates, monkeys, apes | Don't remember pet type | Other | Not applicable at this time | Declined |
| Pet 1 | 🔾 | 🔾 | 🔾 | 🔾 | 🔾 | 🔾 | 🔾 | 🔾 | 🔾 | 🔾 | 🔾 | 🔾 | 🔾 | 🔾 | 🔾 | 🔾 |
| Pet 2 | 🔾 | 🔾 | 🔾 | 🔾 | 🔾 | 🔾 | 🔾 | 🔾 | 🔾 | 🔾 | 🔾 | 🔾 | 🔾 | 🔾 | 🔾 | 🔾 |
| Pet 3 | 🔾 | 🔾 | 🔾 | 🔾 | 🔾 | 🔾 | 🔾 | 🔾 | 🔾 | 🔾 | 🔾 | 🔾 | 🔾 | 🔾 | 🔾 | 🔾 |
| Pet 4 | 🔾 | 🔾 | 🔾 | 🔾 | 🔾 | 🔾 | 🔾 | 🔾 | 🔾 | 🔾 | 🔾 | 🔾 | 🔾 | 🔾 | 🔾 | 🔾 |
| Pet 5 | 🔾 | 🔾 | 🔾 | 🔾 | 🔾 | 🔾 | 🔾 | 🔾 | 🔾 | 🔾 | 🔾 | 🔾 | 🔾 | 🔾 | 🔾 | 🔾 |
| Pet 6 | 🔾 | 🔾 | 🔾 | 🔾 | 🔾 | 🔾 | 🔾 | 🔾 | 🔾 | 🔾 | 🔾 | 🔾 | 🔾 | 🔾 | 🔾 | 🔾 |
| Pet 7 | 🔾 | 🔾 | 🔾 | 🔾 | 🔾 | 🔾 | 🔾 | 🔾 | 🔾 | 🔾 | 🔾 | 🔾 | 🔾 | 🔾 | 🔾 | 🔾 |

**132) Indicate the Total Duration of contact with the Types of Pet.**

|  | Non-MS Subject or Before Onset of MS | | | | | | | | | | | | |
| --- | --- | --- | --- | --- | --- | --- | --- | --- | --- | --- | --- | --- | --- |
|  | 0 to 3 months | 4 to 6 months | 7 months to 1 year | 1.01 to 2 years | 2.01 to 3 years | 3.01 to 5 years | 6 to 10 years | 11 to 15 years | 16 to 20 years | More than 20 years | Don't remember | Not applicable | Declined |
| Dogs | 🔾 | 🔾 | 🔾 | 🔾 | 🔾 | 🔾 | 🔾 | 🔾 | 🔾 | 🔾 | 🔾 | 🔾 | 🔾 |
| Cats | 🔾 | 🔾 | 🔾 | 🔾 | 🔾 | 🔾 | 🔾 | 🔾 | 🔾 | 🔾 | 🔾 | 🔾 | 🔾 |
| Birds | 🔾 | 🔾 | 🔾 | 🔾 | 🔾 | 🔾 | 🔾 | 🔾 | 🔾 | 🔾 | 🔾 | 🔾 | 🔾 |
| Fish | 🔾 | 🔾 | 🔾 | 🔾 | 🔾 | 🔾 | 🔾 | 🔾 | 🔾 | 🔾 | 🔾 | 🔾 | 🔾 |
| Mice | 🔾 | 🔾 | 🔾 | 🔾 | 🔾 | 🔾 | 🔾 | 🔾 | 🔾 | 🔾 | 🔾 | 🔾 | 🔾 |
| Rats | 🔾 | 🔾 | 🔾 | 🔾 | 🔾 | 🔾 | 🔾 | 🔾 | 🔾 | 🔾 | 🔾 | 🔾 | 🔾 |
| Hamsters | 🔾 | 🔾 | 🔾 | 🔾 | 🔾 | 🔾 | 🔾 | 🔾 | 🔾 | 🔾 | 🔾 | 🔾 | 🔾 |
| Guinea Pigs | 🔾 | 🔾 | 🔾 | 🔾 | 🔾 | 🔾 | 🔾 | 🔾 | 🔾 | 🔾 | 🔾 | 🔾 | 🔾 |
| Rabbits | 🔾 | 🔾 | 🔾 | 🔾 | 🔾 | 🔾 | 🔾 | 🔾 | 🔾 | 🔾 | 🔾 | 🔾 | 🔾 |
| Lizards | 🔾 | 🔾 | 🔾 | 🔾 | 🔾 | 🔾 | 🔾 | 🔾 | 🔾 | 🔾 | 🔾 | 🔾 | 🔾 |
| Snakes, other Reptiles | 🔾 | 🔾 | 🔾 | 🔾 | 🔾 | 🔾 | 🔾 | 🔾 | 🔾 | 🔾 | 🔾 | 🔾 | 🔾 |
| Primates, monkeys, apes | 🔾 | 🔾 | 🔾 | 🔾 | 🔾 | 🔾 | 🔾 | 🔾 | 🔾 | 🔾 | 🔾 | 🔾 | 🔾 |
| Other | 🔾 | 🔾 | 🔾 | 🔾 | 🔾 | 🔾 | 🔾 | 🔾 | 🔾 | 🔾 | 🔾 | 🔾 | 🔾 |

|  | After Onset of MS | | | | | | | | | | | | |
| --- | --- | --- | --- | --- | --- | --- | --- | --- | --- | --- | --- | --- | --- |
|  | 0 to 3 months | 4 to 6 months | 7 months to 1 year | 1.01 to 2 years | 2.01 to 3 years | 3.01 to 5 years | 6 to 10 years | 11 to 15 years | 16 to 20 years | More than 20 years | Don't remember | Not applicable | Declined |
| Dogs | 🔾 | 🔾 | 🔾 | 🔾 | 🔾 | 🔾 | 🔾 | 🔾 | 🔾 | 🔾 | 🔾 | 🔾 | 🔾 |
| Cats | 🔾 | 🔾 | 🔾 | 🔾 | 🔾 | 🔾 | 🔾 | 🔾 | 🔾 | 🔾 | 🔾 | 🔾 | 🔾 |
| Birds | 🔾 | 🔾 | 🔾 | 🔾 | 🔾 | 🔾 | 🔾 | 🔾 | 🔾 | 🔾 | 🔾 | 🔾 | 🔾 |
| Fish | 🔾 | 🔾 | 🔾 | 🔾 | 🔾 | 🔾 | 🔾 | 🔾 | 🔾 | 🔾 | 🔾 | 🔾 | 🔾 |
| Mice | 🔾 | 🔾 | 🔾 | 🔾 | 🔾 | 🔾 | 🔾 | 🔾 | 🔾 | 🔾 | 🔾 | 🔾 | 🔾 |
| Rats | 🔾 | 🔾 | 🔾 | 🔾 | 🔾 | 🔾 | 🔾 | 🔾 | 🔾 | 🔾 | 🔾 | 🔾 | 🔾 |
| Hamsters | 🔾 | 🔾 | 🔾 | 🔾 | 🔾 | 🔾 | 🔾 | 🔾 | 🔾 | 🔾 | 🔾 | 🔾 | 🔾 |
| Guinea Pigs | 🔾 | 🔾 | 🔾 | 🔾 | 🔾 | 🔾 | 🔾 | 🔾 | 🔾 | 🔾 | 🔾 | 🔾 | 🔾 |
| Rabbits | 🔾 | 🔾 | 🔾 | 🔾 | 🔾 | 🔾 | 🔾 | 🔾 | 🔾 | 🔾 | 🔾 | 🔾 | 🔾 |
| Lizards | 🔾 | 🔾 | 🔾 | 🔾 | 🔾 | 🔾 | 🔾 | 🔾 | 🔾 | 🔾 | 🔾 | 🔾 | 🔾 |
| Snakes, other Reptiles | 🔾 | 🔾 | 🔾 | 🔾 | 🔾 | 🔾 | 🔾 | 🔾 | 🔾 | 🔾 | 🔾 | 🔾 | 🔾 |
| Primates, monkeys, apes | 🔾 | 🔾 | 🔾 | 🔾 | 🔾 | 🔾 | 🔾 | 🔾 | 🔾 | 🔾 | 🔾 | 🔾 | 🔾 |
| Other | 🔾 | 🔾 | 🔾 | 🔾 | 🔾 | 🔾 | 🔾 | 🔾 | 🔾 | 🔾 | 🔾 | 🔾 | 🔾 |

**133) Comments Regarding Pets** ____________________________________________________________

**134) Did you have contact with Farm Animals for more than Three Months? Please answer the question if you had regular contact or were involved in cleaning, care or contact with manure. These contacts would be routine in agricultural settings.**

🔾 Yes 🔾 No 🔾 Don't Know 🔾 Declined

**135) Type of Farm Animal.**

Non-MS Subject or Before Onset of MS

|  | Farm Animal 1 | Farm Animal 2 | Farm Animal 3 | Farm Animal 4 | Farm Animal 5 | Farm Animal 6 |
| --- | --- | --- | --- | --- | --- | --- |
| Cows, Buffalo, Cattle | 🔾 | 🔾 | 🔾 | 🔾 | 🔾 | 🔾 |
| Pigs, Swine | 🔾 | 🔾 | 🔾 | 🔾 | 🔾 | 🔾 |
| Chickens, Hens | 🔾 | 🔾 | 🔾 | 🔾 | 🔾 | 🔾 |
| Ducks, Geese | 🔾 | 🔾 | 🔾 | 🔾 | 🔾 | 🔾 |
| Goats | 🔾 | 🔾 | 🔾 | 🔾 | 🔾 | 🔾 |
| Sheep | 🔾 | 🔾 | 🔾 | 🔾 | 🔾 | 🔾 |
| Horses | 🔾 | 🔾 | 🔾 | 🔾 | 🔾 | 🔾 |
| Donkeys, Mules | 🔾 | 🔾 | 🔾 | 🔾 | 🔾 | 🔾 |
| Camels, Llamas, vicuna | 🔾 | 🔾 | 🔾 | 🔾 | 🔾 | 🔾 |
| Zoo animals | 🔾 | 🔾 | 🔾 | 🔾 | 🔾 | 🔾 |
| Fish farming | 🔾 | 🔾 | 🔾 | 🔾 | 🔾 | 🔾 |
| Oysters, shrimp, lobsters | 🔾 | 🔾 | 🔾 | 🔾 | 🔾 | 🔾 |
| Marine animals | 🔾 | 🔾 | 🔾 | 🔾 | 🔾 | 🔾 |
| Bees, e.g. beekeeping | 🔾 | 🔾 | 🔾 | 🔾 | 🔾 | 🔾 |
| Other (specify) | 🔾 | 🔾 | 🔾 | 🔾 | 🔾 | 🔾 |
| Declined | 🔾 | 🔾 | 🔾 | 🔾 | 🔾 | 🔾 |
| Not applicable at this time | 🔾 | 🔾 | 🔾 | 🔾 | 🔾 | 🔾 |

After Onset of MS

|  | Farm Animal 1 | Farm Animal 2 | Farm Animal 3 | Farm Animal 4 | Farm Animal 5 | Farm Animal 6 |
| --- | --- | --- | --- | --- | --- | --- |
| Cows, Buffalo, Cattle | 🔾 | 🔾 | 🔾 | 🔾 | 🔾 | 🔾 |
| Pigs, Swine | 🔾 | 🔾 | 🔾 | 🔾 | 🔾 | 🔾 |
| Chickens, Hens | 🔾 | 🔾 | 🔾 | 🔾 | 🔾 | 🔾 |
| Ducks, Geese | 🔾 | 🔾 | 🔾 | 🔾 | 🔾 | 🔾 |
| Goats | 🔾 | 🔾 | 🔾 | 🔾 | 🔾 | 🔾 |
| Sheep | 🔾 | 🔾 | 🔾 | 🔾 | 🔾 | 🔾 |
| Horses | 🔾 | 🔾 | 🔾 | 🔾 | 🔾 | 🔾 |
| Donkeys, Mules | 🔾 | 🔾 | 🔾 | 🔾 | 🔾 | 🔾 |
| Camels, Llamas, vicuna | 🔾 | 🔾 | 🔾 | 🔾 | 🔾 | 🔾 |
| Zoo animals | 🔾 | 🔾 | 🔾 | 🔾 | 🔾 | 🔾 |
| Fish farming | 🔾 | 🔾 | 🔾 | 🔾 | 🔾 | 🔾 |
| Oysters, shrimp, lobsters | 🔾 | 🔾 | 🔾 | 🔾 | 🔾 | 🔾 |
| Marine animals | 🔾 | 🔾 | 🔾 | 🔾 | 🔾 | 🔾 |
| Bees, e.g. beekeeping | 🔾 | 🔾 | 🔾 | 🔾 | 🔾 | 🔾 |
| Other (specify) | 🔾 | 🔾 | 🔾 | 🔾 | 🔾 | 🔾 |
| Declined | 🔾 | 🔾 | 🔾 | 🔾 | 🔾 | 🔾 |
| Not applicable at this time | 🔾 | 🔾 | 🔾 | 🔾 | 🔾 | 🔾 |

Last 3-months?

|  | Farm Animal 1 | Farm Animal 2 | Farm Animal 3 | Farm Animal 4 | Farm Animal 5 | Farm Animal 6 |
| --- | --- | --- | --- | --- | --- | --- |
| Cows, Buffalo, Cattle | 🔾 | 🔾 | 🔾 | 🔾 | 🔾 | 🔾 |
| Pigs, Swine | 🔾 | 🔾 | 🔾 | 🔾 | 🔾 | 🔾 |
| Chickens, Hens | 🔾 | 🔾 | 🔾 | 🔾 | 🔾 | 🔾 |
| Ducks, Geese | 🔾 | 🔾 | 🔾 | 🔾 | 🔾 | 🔾 |
| Goats | 🔾 | 🔾 | 🔾 | 🔾 | 🔾 | 🔾 |
| Sheep | 🔾 | 🔾 | 🔾 | 🔾 | 🔾 | 🔾 |
| Horses | 🔾 | 🔾 | 🔾 | 🔾 | 🔾 | 🔾 |
| Donkeys, Mules | 🔾 | 🔾 | 🔾 | 🔾 | 🔾 | 🔾 |
| Camels, Llamas, vicuna | 🔾 | 🔾 | 🔾 | 🔾 | 🔾 | 🔾 |
| Zoo animals | 🔾 | 🔾 | 🔾 | 🔾 | 🔾 | 🔾 |
| Fish farming | 🔾 | 🔾 | 🔾 | 🔾 | 🔾 | 🔾 |
| Oysters, shrimp, lobsters | 🔾 | 🔾 | 🔾 | 🔾 | 🔾 | 🔾 |
| Marine animals | 🔾 | 🔾 | 🔾 | 🔾 | 🔾 | 🔾 |
| Bees, e.g. beekeeping | 🔾 | 🔾 | 🔾 | 🔾 | 🔾 | 🔾 |
| Other (specify) | 🔾 | 🔾 | 🔾 | 🔾 | 🔾 | 🔾 |
| Declined | 🔾 | 🔾 | 🔾 | 🔾 | 🔾 | 🔾 |
| Not applicable at this time | 🔾 | 🔾 | 🔾 | 🔾 | 🔾 | 🔾 |

**136) Indicate the Total Duration of contact with the Types of Farm Animal Above.**

|  | Non-MS Subject or Before Onset of MS | | | | | | | | | | | | | | |
| --- | --- | --- | --- | --- | --- | --- | --- | --- | --- | --- | --- | --- | --- | --- | --- |
|  | 0 to 3 months | 4 to 6 months | 7 months to 1 yr | 1.01 to 2 yrs | 2.01 to 3 years | 3.01 to 5 years | 6 to 10 years | 11 to 15 years | 16 to 20 years | More than 20 years | Don't remember | | Not applicable at this time | | Declined |
| Cows, Buffalo, Cattle | 🔾 | 🔾 | 🔾 | 🔾 | 🔾 | 🔾 | 🔾 | 🔾 | 🔾 | 🔾 | 🔾 | 🔾 | | 🔾 | |
| Pigs, Swine | 🔾 | 🔾 | 🔾 | 🔾 | 🔾 | 🔾 | 🔾 | 🔾 | 🔾 | 🔾 | 🔾 | 🔾 | | 🔾 | |
| Chickens, hens | 🔾 | 🔾 | 🔾 | 🔾 | 🔾 | 🔾 | 🔾 | 🔾 | 🔾 | 🔾 | 🔾 | 🔾 | | 🔾 | |
| Ducks, Geese | 🔾 | 🔾 | 🔾 | 🔾 | 🔾 | 🔾 | 🔾 | 🔾 | 🔾 | 🔾 | 🔾 | 🔾 | | 🔾 | |
| Goats | 🔾 | 🔾 | 🔾 | 🔾 | 🔾 | 🔾 | 🔾 | 🔾 | 🔾 | 🔾 | 🔾 | 🔾 | | 🔾 | |
| Sheep | 🔾 | 🔾 | 🔾 | 🔾 | 🔾 | 🔾 | 🔾 | 🔾 | 🔾 | 🔾 | 🔾 | 🔾 | | 🔾 | |
| Horses | 🔾 | 🔾 | 🔾 | 🔾 | 🔾 | 🔾 | 🔾 | 🔾 | 🔾 | 🔾 | 🔾 | 🔾 | | 🔾 | |
| Donkeys, Mules | 🔾 | 🔾 | 🔾 | 🔾 | 🔾 | 🔾 | 🔾 | 🔾 | 🔾 | 🔾 | 🔾 | 🔾 | | 🔾 | |
| Camels, Llamas, Vicunas | 🔾 | 🔾 | 🔾 | 🔾 | 🔾 | 🔾 | 🔾 | 🔾 | 🔾 | 🔾 | 🔾 | 🔾 | | 🔾 | |
| Zoo Animals | 🔾 | 🔾 | 🔾 | 🔾 | 🔾 | 🔾 | 🔾 | 🔾 | 🔾 | 🔾 | 🔾 | 🔾 | | 🔾 | |
| Fish farming | 🔾 | 🔾 | 🔾 | 🔾 | 🔾 | 🔾 | 🔾 | 🔾 | 🔾 | 🔾 | 🔾 | 🔾 | | 🔾 | |
| Oysters, Shrimp, Lobsters | 🔾 | 🔾 | 🔾 | 🔾 | 🔾 | 🔾 | 🔾 | 🔾 | 🔾 | 🔾 | 🔾 | 🔾 | | 🔾 | |
| Marine animals | 🔾 | 🔾 | 🔾 | 🔾 | 🔾 | 🔾 | 🔾 | 🔾 | 🔾 | 🔾 | 🔾 | 🔾 | | 🔾 | |
| Bees, e.g., beekeeping | 🔾 | 🔾 | 🔾 | 🔾 | 🔾 | 🔾 | 🔾 | 🔾 | 🔾 | 🔾 | 🔾 | 🔾 | | 🔾 | |
| Other (specify) | 🔾 | 🔾 | 🔾 | 🔾 | 🔾 | 🔾 | 🔾 | 🔾 | 🔾 | 🔾 | 🔾 | 🔾 | | 🔾 | |
| Not applicable | 🔾 | 🔾 | 🔾 | 🔾 | 🔾 | 🔾 | 🔾 | 🔾 | 🔾 | 🔾 | 🔾 | 🔾 | | 🔾 | |
| None at this stage | 🔾 | 🔾 | 🔾 | 🔾 | 🔾 | 🔾 | 🔾 | 🔾 | 🔾 | 🔾 | 🔾 | 🔾 | | 🔾 | |
| Declined | 🔾 | 🔾 | 🔾 | 🔾 | 🔾 | 🔾 | 🔾 | 🔾 | 🔾 | 🔾 | 🔾 | 🔾 | | 🔾 | |

|  | After Onset of MS | | | | | | | | | | | | |
| --- | --- | --- | --- | --- | --- | --- | --- | --- | --- | --- | --- | --- | --- |
|  | 0 to 3 months | 4 to 6 months | 7 months to 1 yr | 1.01 to 2 yrs | 2.01 to 3 years | 3.01 to 5 years | 6 to 10 years | 11 to 15 years | 16 to 20 years | More than 20 years | Don't remember | Not applicable at this time | Declined |
| Cows, Buffalo, Cattle | 🔾 | 🔾 | 🔾 | 🔾 | 🔾 | 🔾 | 🔾 | 🔾 | 🔾 | 🔾 | 🔾 | 🔾 | 🔾 |
| Pigs, Swine | 🔾 | 🔾 | 🔾 | 🔾 | 🔾 | 🔾 | 🔾 | 🔾 | 🔾 | 🔾 | 🔾 | 🔾 | 🔾 |
| Chickens, hens | 🔾 | 🔾 | 🔾 | 🔾 | 🔾 | 🔾 | 🔾 | 🔾 | 🔾 | 🔾 | 🔾 | 🔾 | 🔾 |
| Ducks, Geese | 🔾 | 🔾 | 🔾 | 🔾 | 🔾 | 🔾 | 🔾 | 🔾 | 🔾 | 🔾 | 🔾 | 🔾 | 🔾 |
| Goats | 🔾 | 🔾 | 🔾 | 🔾 | 🔾 | 🔾 | 🔾 | 🔾 | 🔾 | 🔾 | 🔾 | 🔾 | 🔾 |
| Sheep | 🔾 | 🔾 | 🔾 | 🔾 | 🔾 | 🔾 | 🔾 | 🔾 | 🔾 | 🔾 | 🔾 | 🔾 | 🔾 |
| Horses | 🔾 | 🔾 | 🔾 | 🔾 | 🔾 | 🔾 | 🔾 | 🔾 | 🔾 | 🔾 | 🔾 | 🔾 | 🔾 |
| Donkeys, Mules | 🔾 | 🔾 | 🔾 | 🔾 | 🔾 | 🔾 | 🔾 | 🔾 | 🔾 | 🔾 | 🔾 | 🔾 | 🔾 |
| Camels, Llamas, Vicunas | 🔾 | 🔾 | 🔾 | 🔾 | 🔾 | 🔾 | 🔾 | 🔾 | 🔾 | 🔾 | 🔾 | 🔾 | 🔾 |
| Zoo Animals | 🔾 | 🔾 | 🔾 | 🔾 | 🔾 | 🔾 | 🔾 | 🔾 | 🔾 | 🔾 | 🔾 | 🔾 | 🔾 |
| Fish farming | 🔾 | 🔾 | 🔾 | 🔾 | 🔾 | 🔾 | 🔾 | 🔾 | 🔾 | 🔾 | 🔾 | 🔾 | 🔾 |
| Oysters, Shrimp, Lobsters | 🔾 | 🔾 | 🔾 | 🔾 | 🔾 | 🔾 | 🔾 | 🔾 | 🔾 | 🔾 | 🔾 | 🔾 | 🔾 |
| Marine animals | 🔾 | 🔾 | 🔾 | 🔾 | 🔾 | 🔾 | 🔾 | 🔾 | 🔾 | 🔾 | 🔾 | 🔾 | 🔾 |
| Bees, e.g., beekeeping | 🔾 | 🔾 | 🔾 | 🔾 | 🔾 | 🔾 | 🔾 | 🔾 | 🔾 | 🔾 | 🔾 | 🔾 | 🔾 |
| Other (specify) | 🔾 | 🔾 | 🔾 | 🔾 | 🔾 | 🔾 | 🔾 | 🔾 | 🔾 | 🔾 | 🔾 | 🔾 | 🔾 |
| Not applicable | 🔾 | 🔾 | 🔾 | 🔾 | 🔾 | 🔾 | 🔾 | 🔾 | 🔾 | 🔾 | 🔾 | 🔾 | 🔾 |
| None at this stage | 🔾 | 🔾 | 🔾 | 🔾 | 🔾 | 🔾 | 🔾 | 🔾 | 🔾 | 🔾 | 🔾 | 🔾 | 🔾 |
| Declined | 🔾 | 🔾 | 🔾 | 🔾 | 🔾 | 🔾 | 🔾 | 🔾 | 🔾 | 🔾 | 🔾 | 🔾 | 🔾 |

**137) Comments regarding Farm Animals** _________________________________________________________

**138) Did you have contact with tissues, pathogens, meat (not for consumption), blood, feces for more than Three months as part of your job or otherwise.**

🔾 Yes 🔾 No 🔾 Don't Know 🔾 Declined

**139) Type of tissue, pathogen or meat.**

Non-MS Subject or Before Onset of MS

|  | Tissue Type 1 | Tissue Type 2 | Tissue Type 3 | Tissue Type 4 | Tissue Type 5 | Tissue Type 6 |
| --- | --- | --- | --- | --- | --- | --- |
| Human Surgery | 🔾 | 🔾 | 🔾 | 🔾 | 🔾 | 🔾 |
| Human Corpses | 🔾 | 🔾 | 🔾 | 🔾 | 🔾 | 🔾 |
| Human Blood, Pathology specimens | 🔾 | 🔾 | 🔾 | 🔾 | 🔾 | 🔾 |
| Patients with Infectious Diseases | 🔾 | 🔾 | 🔾 | 🔾 | 🔾 | 🔾 |
| Bovine Brain or Spinal Tissue | 🔾 | 🔾 | 🔾 | 🔾 | 🔾 | 🔾 |
| Pig Brain or Spinal Tissue | 🔾 | 🔾 | 🔾 | 🔾 | 🔾 | 🔾 |
| Meat Processing | 🔾 | 🔾 | 🔾 | 🔾 | 🔾 | 🔾 |
| Fish Processing | 🔾 | 🔾 | 🔾 | 🔾 | 🔾 | 🔾 |
| Wild Game Processing | 🔾 | 🔾 | 🔾 | 🔾 | 🔾 | 🔾 |
| Dissection, Surgery of Rodents, Other Animals | 🔾 | 🔾 | 🔾 | 🔾 | 🔾 | 🔾 |
| Bacteria, Virus, Cell Cultures | 🔾 | 🔾 | 🔾 | 🔾 | 🔾 | 🔾 |
| Other (specify) | 🔾 | 🔾 | 🔾 | 🔾 | 🔾 | 🔾 |
| Not applicable at this time | | | | | | |
| Declined | | | | | | |

After Onset of MS

|  | Tissue Type 1 | Tissue Type 2 | Tissue Type 3 | Tissue Type 4 | Tissue Type 5 | Tissue Type 6 |
| --- | --- | --- | --- | --- | --- | --- |
| Human Surgery | 🔾 | 🔾 | 🔾 | 🔾 | 🔾 | 🔾 |
| Human Corpses | 🔾 | 🔾 | 🔾 | 🔾 | 🔾 | 🔾 |
| Human Blood, Pathology specimens | 🔾 | 🔾 | 🔾 | 🔾 | 🔾 | 🔾 |
| Patients with Infectious Diseases | 🔾 | 🔾 | 🔾 | 🔾 | 🔾 | 🔾 |
| Bovine Brain or Spinal Tissue | 🔾 | 🔾 | 🔾 | 🔾 | 🔾 | 🔾 |
| Pig Brain or Spinal Tissue | 🔾 | 🔾 | 🔾 | 🔾 | 🔾 | 🔾 |
| Meat Processing | 🔾 | 🔾 | 🔾 | 🔾 | 🔾 | 🔾 |
| Fish Processing | 🔾 | 🔾 | 🔾 | 🔾 | 🔾 | 🔾 |
| Wild Game Processing | 🔾 | 🔾 | 🔾 | 🔾 | 🔾 | 🔾 |
| Dissection, Surgery of Rodents, Other Animals | 🔾 | 🔾 | 🔾 | 🔾 | 🔾 | 🔾 |
| Bacteria, Virus, Cell Cultures | 🔾 | 🔾 | 🔾 | 🔾 | 🔾 | 🔾 |
| Other (specify) | 🔾 | 🔾 | 🔾 | 🔾 | 🔾 | 🔾 |
| Not applicable at this time | | | | | | |
| Declined | | | | | | |

Last 3-Months?

|  | Tissue Type 1 | Tissue Type 2 | Tissue Type 3 | Tissue Type 4 | Tissue Type 5 | Tissue Type 6 |
| --- | --- | --- | --- | --- | --- | --- |
| Human Surgery | 🔾 | 🔾 | 🔾 | 🔾 | 🔾 | 🔾 |
| Human Corpses | 🔾 | 🔾 | 🔾 | 🔾 | 🔾 | 🔾 |
| Human Blood, Pathology specimens | 🔾 | 🔾 | 🔾 | 🔾 | 🔾 | 🔾 |
| Patients with Infectious Diseases | 🔾 | 🔾 | 🔾 | 🔾 | 🔾 | 🔾 |
| Bovine Brain or Spinal Tissue | 🔾 | 🔾 | 🔾 | 🔾 | 🔾 | 🔾 |
| Pig Brain or Spinal Tissue | 🔾 | 🔾 | 🔾 | 🔾 | 🔾 | 🔾 |
| Meat Processing | 🔾 | 🔾 | 🔾 | 🔾 | 🔾 | 🔾 |
| Fish Processing | 🔾 | 🔾 | 🔾 | 🔾 | 🔾 | 🔾 |
| Wild Game Processing | 🔾 | 🔾 | 🔾 | 🔾 | 🔾 | 🔾 |
| Dissection, Surgery of Rodents, Other Animals | 🔾 | 🔾 | 🔾 | 🔾 | 🔾 | 🔾 |
| Bacteria, Virus, Cell Cultures | 🔾 | 🔾 | 🔾 | 🔾 | 🔾 | 🔾 |
| Other (specify) | 🔾 | 🔾 | 🔾 | 🔾 | 🔾 | 🔾 |
| Not applicable at this time | | | | | | |
| Declined | | | | | | |

**140) Indicate the Total Duration of contact with the Types of Tissue, Pathogen or Meat Above.**

|  | Non-MS Subject or Before Onset of MS | | | | | | | | | | | | |
| --- | --- | --- | --- | --- | --- | --- | --- | --- | --- | --- | --- | --- | --- |
|  | 0 to 3 months | 4 to 6 months | 7 months to 1 year | 1.01 to 2 years | 2.01 to 3 years | 3.01 to 5 years | 6 to 10 years | 11 to 15 years | 16 to 20 years | More than 20 years | Don't remember | Not applicable at this time | Declined |
| Human Surgery | 🔾 | 🔾 | 🔾 | 🔾 | 🔾 | 🔾 | 🔾 | 🔾 | 🔾 | 🔾 | 🔾 | 🔾 | 🔾 |
| Human Corpses | 🔾 | 🔾 | 🔾 | 🔾 | 🔾 | 🔾 | 🔾 | 🔾 | 🔾 | 🔾 | 🔾 | 🔾 | 🔾 |
| Human Blood, Pathology Specimens | 🔾 | 🔾 | 🔾 | 🔾 | 🔾 | 🔾 | 🔾 | 🔾 | 🔾 | 🔾 | 🔾 | 🔾 | 🔾 |
| Patients with Infectious Diseases | 🔾 | 🔾 | 🔾 | 🔾 | 🔾 | 🔾 | 🔾 | 🔾 | 🔾 | 🔾 | 🔾 | 🔾 | 🔾 |
| Bovine Brain or Spinal Tissue | 🔾 | 🔾 | 🔾 | 🔾 | 🔾 | 🔾 | 🔾 | 🔾 | 🔾 | 🔾 | 🔾 | 🔾 | 🔾 |
| Pig Brain or Spinal Tissue | 🔾 | 🔾 | 🔾 | 🔾 | 🔾 | 🔾 | 🔾 | 🔾 | 🔾 | 🔾 | 🔾 | 🔾 | 🔾 |
| Meat Processing | 🔾 | 🔾 | 🔾 | 🔾 | 🔾 | 🔾 | 🔾 | 🔾 | 🔾 | 🔾 | 🔾 | 🔾 | 🔾 |
| Fish Processing | 🔾 | 🔾 | 🔾 | 🔾 | 🔾 | 🔾 | 🔾 | 🔾 | 🔾 | 🔾 | 🔾 | 🔾 | 🔾 |
| Wild Game Processing | 🔾 | 🔾 | 🔾 | 🔾 | 🔾 | 🔾 | 🔾 | 🔾 | 🔾 | 🔾 | 🔾 | 🔾 | 🔾 |
| Dissection, Surgery of Rodents, Other Animals | 🔾 | 🔾 | 🔾 | 🔾 | 🔾 | 🔾 | 🔾 | 🔾 | 🔾 | 🔾 | 🔾 | 🔾 | 🔾 |
| Other | 🔾 | 🔾 | 🔾 | 🔾 | 🔾 | 🔾 | 🔾 | 🔾 | 🔾 | 🔾 | 🔾 | 🔾 | 🔾 |
| Not applicable | 🔾 | 🔾 | 🔾 | 🔾 | 🔾 | 🔾 | 🔾 | 🔾 | 🔾 | 🔾 | 🔾 | 🔾 | 🔾 |
| None at this stage | 🔾 | 🔾 | 🔾 | 🔾 | 🔾 | 🔾 | 🔾 | 🔾 | 🔾 | 🔾 | 🔾 | 🔾 | 🔾 |
| Declined | 🔾 | 🔾 | 🔾 | 🔾 | 🔾 | 🔾 | 🔾 | 🔾 | 🔾 | 🔾 | 🔾 | 🔾 | 🔾 |

|  | After Onset of MS | | | | | | | | | | | | |
| --- | --- | --- | --- | --- | --- | --- | --- | --- | --- | --- | --- | --- | --- |
|  | 0 to 3 months | 4 to 6 months | 7 months to 1 year | 1.01 to 2 years | 2.01 to 3 years | 3.01 to 5 years | 6 to 10 years | 11 to 15 years | 16 to 20 years | More than 20 years | Don't remember | Not applicable at this time | Declined |
| Human Surgery | 🔾 | 🔾 | 🔾 | 🔾 | 🔾 | 🔾 | 🔾 | 🔾 | 🔾 | 🔾 | 🔾 | 🔾 | 🔾 |
| Human Corpses | 🔾 | 🔾 | 🔾 | 🔾 | 🔾 | 🔾 | 🔾 | 🔾 | 🔾 | 🔾 | 🔾 | 🔾 | 🔾 |
| Human Blood, Pathology Specimens | 🔾 | 🔾 | 🔾 | 🔾 | 🔾 | 🔾 | 🔾 | 🔾 | 🔾 | 🔾 | 🔾 | 🔾 | 🔾 |
| Patients with Infectious Diseases | 🔾 | 🔾 | 🔾 | 🔾 | 🔾 | 🔾 | 🔾 | 🔾 | 🔾 | 🔾 | 🔾 | 🔾 | 🔾 |
| Bovine Brain or Spinal Tissue | 🔾 | 🔾 | 🔾 | 🔾 | 🔾 | 🔾 | 🔾 | 🔾 | 🔾 | 🔾 | 🔾 | 🔾 | 🔾 |
| Pig Brain or Spinal Tissue | 🔾 | 🔾 | 🔾 | 🔾 | 🔾 | 🔾 | 🔾 | 🔾 | 🔾 | 🔾 | 🔾 | 🔾 | 🔾 |
| Meat Processing | 🔾 | 🔾 | 🔾 | 🔾 | 🔾 | 🔾 | 🔾 | 🔾 | 🔾 | 🔾 | 🔾 | 🔾 | 🔾 |
| Fish Processing | 🔾 | 🔾 | 🔾 | 🔾 | 🔾 | 🔾 | 🔾 | 🔾 | 🔾 | 🔾 | 🔾 | 🔾 | 🔾 |
| Wild Game Processing | 🔾 | 🔾 | 🔾 | 🔾 | 🔾 | 🔾 | 🔾 | 🔾 | 🔾 | 🔾 | 🔾 | 🔾 | 🔾 |
| Dissection, Surgery of Rodents, Other Animals | 🔾 | 🔾 | 🔾 | 🔾 | 🔾 | 🔾 | 🔾 | 🔾 | 🔾 | 🔾 | 🔾 | 🔾 | 🔾 |
| Other | 🔾 | 🔾 | 🔾 | 🔾 | 🔾 | 🔾 | 🔾 | 🔾 | 🔾 | 🔾 | 🔾 | 🔾 | 🔾 |
| Not applicable | 🔾 | 🔾 | 🔾 | 🔾 | 🔾 | 🔾 | 🔾 | 🔾 | 🔾 | 🔾 | 🔾 | 🔾 | 🔾 |
| None at this stage | 🔾 | 🔾 | 🔾 | 🔾 | 🔾 | 🔾 | 🔾 | 🔾 | 🔾 | 🔾 | 🔾 | 🔾 | 🔾 |
| Declined | 🔾 | 🔾 | 🔾 | 🔾 | 🔾 | 🔾 | 🔾 | 🔾 | 🔾 | 🔾 | 🔾 | 🔾 | 🔾 |

**141) Comments regarding Tissues, Pathogen or Meat** ____________________________________________

**142) Did you have regular contact with Chemicals or Extreme Physical Conditions on a regular basis as part of your job or otherwise.**

🔾 Yes 🔾 No 🔾 Don't Know 🔾 Declined

**143) Type of Chemical or Physical Condition.**

| Non-MS Subject or Before Onset of MS | | | | | |
| --- | --- | --- | --- | --- | --- |
|  | Chemical or condition 1 | Chemical or condition 2 | Chemical or condition 3 | Chemical or condition 4 | Chemical or condition 5 |
| Lead, zinc, heavy metals | 🔾 | 🔾 | 🔾 | 🔾 | 🔾 |
| Mining, ores | 🔾 | 🔾 | 🔾 | 🔾 | 🔾 |
| Steelmaking, smelting, foundry operations | 🔾 | 🔾 | 🔾 | 🔾 | 🔾 |
| Coal, coal dust, coal boilers, coal fires | 🔾 | 🔾 | 🔾 | 🔾 | 🔾 |
| Pesticides | 🔾 | 🔾 | 🔾 | 🔾 | 🔾 |
| Herbicides | 🔾 | 🔾 | 🔾 | 🔾 | 🔾 |
| Fungicides | 🔾 | 🔾 | 🔾 | 🔾 | 🔾 |
| Dyes, printing inks | 🔾 | 🔾 | 🔾 | 🔾 | 🔾 |
| Adhesives | 🔾 | 🔾 | 🔾 | 🔾 | 🔾 |
| Paint, paint thinners, paint strippers, varnishes | 🔾 | 🔾 | 🔾 | 🔾 | 🔾 |
| Cutting, cooling, or lubricating oils. | 🔾 | 🔾 | 🔾 | 🔾 | 🔾 |
| Gasoline, diesel, aviation fuel | 🔾 | 🔾 | 🔾 | 🔾 | 🔾 |
| Antifreeze, refrigerants | 🔾 | 🔾 | 🔾 | 🔾 | 🔾 |
| Petrochemicals, plastics, monomers | 🔾 | 🔾 | 🔾 | 🔾 | 🔾 |
| Aromatic solvents, benzene, toluene, xylene, phenol | 🔾 | 🔾 | 🔾 | 🔾 | 🔾 |
| Automobile exhaust, dust, particulates | 🔾 | 🔾 | 🔾 | 🔾 | 🔾 |
| Soaps, detergents manufacturing | 🔾 | 🔾 | 🔾 | 🔾 | 🔾 |
| Fine chemicals, perfumes, drugs manufacture or dispensing | 🔾 | 🔾 | 🔾 | 🔾 | 🔾 |
| Purified biochemicals, enzymes | 🔾 | 🔾 | 🔾 | 🔾 | 🔾 |
| Inorganic acids, bases | 🔾 | 🔾 | 🔾 | 🔾 | 🔾 |
| Organic acids, bases | 🔾 | 🔾 | 🔾 | 🔾 | 🔾 |
| Solvents, degreasing agents | 🔾 | 🔾 | 🔾 | 🔾 | 🔾 |
| Dry cleaning agents | 🔾 | 🔾 | 🔾 | 🔾 | 🔾 |
| Nail salon products | 🔾 | 🔾 | 🔾 | 🔾 | 🔾 |
| Hairdressing products. | 🔾 | 🔾 | 🔾 | 🔾 | 🔾 |
| Building materials, cement, concrete. | 🔾 | 🔾 | 🔾 | 🔾 | 🔾 |
| Road, highway public works, asphalt, coal tar. | 🔾 | 🔾 | 🔾 | 🔾 | 🔾 |
| Mud, sand, stone, quarrying, farming | 🔾 | 🔾 | 🔾 | 🔾 | 🔾 |
| Forest, forestry, logging, saw mill | 🔾 | 🔾 | 🔾 | 🔾 | 🔾 |
| Intense heat: Fires, fire fighting, furnaces, ovens, kilns | 🔾 | 🔾 | 🔾 | 🔾 | 🔾 |
| Waste water, pond water, lake water, marshland, sewage | 🔾 | 🔾 | 🔾 | 🔾 | 🔾 |
| Other (specify) | 🔾 | 🔾 | 🔾 | 🔾 | 🔾 |
| Not applicable at this time | 🔾 | 🔾 | 🔾 | 🔾 | 🔾 |
| Declined | 🔾 | 🔾 | 🔾 | 🔾 | 🔾 |

| After Onset of MS | | | | | |
| --- | --- | --- | --- | --- | --- |
|  | Chemical or condition 1 | Chemical or condition 2 | Chemical or condition 3 | Chemical or condition 4 | Chemical or condition 5 |
| Lead, zinc, heavy metals | 🔾 | 🔾 | 🔾 | 🔾 | 🔾 |
| Mining, ores | 🔾 | 🔾 | 🔾 | 🔾 | 🔾 |
| Steelmaking, smelting, foundry operations | 🔾 | 🔾 | 🔾 | 🔾 | 🔾 |
| Coal, coal dust, coal boilers, coal fires | 🔾 | 🔾 | 🔾 | 🔾 | 🔾 |
| Pesticides | 🔾 | 🔾 | 🔾 | 🔾 | 🔾 |
| Herbicides | 🔾 | 🔾 | 🔾 | 🔾 | 🔾 |
| Fungicides | 🔾 | 🔾 | 🔾 | 🔾 | 🔾 |
| Dyes, printing inks | 🔾 | 🔾 | 🔾 | 🔾 | 🔾 |
| Adhesives | 🔾 | 🔾 | 🔾 | 🔾 | 🔾 |
| Paint, paint thinners, paint strippers, varnishes | 🔾 | 🔾 | 🔾 | 🔾 | 🔾 |
| Cutting, cooling, or lubricating oils. | 🔾 | 🔾 | 🔾 | 🔾 | 🔾 |
| Gasoline, diesel, aviation fuel | 🔾 | 🔾 | 🔾 | 🔾 | 🔾 |
| Antifreeze, refrigerants | 🔾 | 🔾 | 🔾 | 🔾 | 🔾 |
| Petrochemicals, plastics, monomers | 🔾 | 🔾 | 🔾 | 🔾 | 🔾 |
| Aromatic solvents, benzene, toluene, xylene, phenol | 🔾 | 🔾 | 🔾 | 🔾 | 🔾 |
| Automobile exhaust, dust, particulates | 🔾 | 🔾 | 🔾 | 🔾 | 🔾 |
| Soaps, detergents manufacturing | 🔾 | 🔾 | 🔾 | 🔾 | 🔾 |
| Fine chemicals, perfumes, drugs manufacture or dispensing | 🔾 | 🔾 | 🔾 | 🔾 | 🔾 |
| Purified biochemicals, enzymes | 🔾 | 🔾 | 🔾 | 🔾 | 🔾 |
| Inorganic acids, bases | 🔾 | 🔾 | 🔾 | 🔾 | 🔾 |
| Organic acids, bases | 🔾 | 🔾 | 🔾 | 🔾 | 🔾 |
| Solvents, degreasing agents | 🔾 | 🔾 | 🔾 | 🔾 | 🔾 |
| Dry cleaning agents | 🔾 | 🔾 | 🔾 | 🔾 | 🔾 |
| Nail salon products | 🔾 | 🔾 | 🔾 | 🔾 | 🔾 |
| Hairdressing products. | 🔾 | 🔾 | 🔾 | 🔾 | 🔾 |
| Building materials, cement, concrete. | 🔾 | 🔾 | 🔾 | 🔾 | 🔾 |
| Road, highway public works, asphalt, coal tar. | 🔾 | 🔾 | 🔾 | 🔾 | 🔾 |
| Mud, sand, stone, quarrying, farming | 🔾 | 🔾 | 🔾 | 🔾 | 🔾 |
| Forest, forestry, logging, saw mill | 🔾 | 🔾 | 🔾 | 🔾 | 🔾 |
| Intense heat: Fires, fire fighting, furnaces, ovens, kilns | 🔾 | 🔾 | 🔾 | 🔾 | 🔾 |
| Waste water, pond water, lake water, marshland, sewage | 🔾 | 🔾 | 🔾 | 🔾 | 🔾 |
| Other (specify) | 🔾 | 🔾 | 🔾 | 🔾 | 🔾 |
| Not applicable at this time | 🔾 | 🔾 | 🔾 | 🔾 | 🔾 |
| Declined | 🔾 | 🔾 | 🔾 | 🔾 | 🔾 |

**144) Indicate the Total Duration of contact with the Chemical or Extreme Physical Condition Above.**

|  | Non-MS Subject or Before Onset of MS | | | | | | | | | | | | |
| --- | --- | --- | --- | --- | --- | --- | --- | --- | --- | --- | --- | --- | --- |
|  | 0 to 3 months | 4 to 6 months | 7 months to 1 year | 1.01 to 2 years | 2.01 to 3 years | 3.01 to 5 years | 6 to 10 years | 11 to 15 years | 16 to 20 years | More than 20 years | Don't remember | Not applicable at this time | Declined |
| Lead, zinc, heavy metals | 🔾 | 🔾 | 🔾 | 🔾 | 🔾 | 🔾 | 🔾 | 🔾 | 🔾 | 🔾 | 🔾 | 🔾 | 🔾 |
| Mining, ores | 🔾 | 🔾 | 🔾 | 🔾 | 🔾 | 🔾 | 🔾 | 🔾 | 🔾 | 🔾 | 🔾 | 🔾 | 🔾 |
| Steelmaking, smelting, foundry operations | 🔾 | 🔾 | 🔾 | 🔾 | 🔾 | 🔾 | 🔾 | 🔾 | 🔾 | 🔾 | 🔾 | 🔾 | 🔾 |
| Coal, coal dust, coal boilers, coal fires | 🔾 | 🔾 | 🔾 | 🔾 | 🔾 | 🔾 | 🔾 | 🔾 | 🔾 | 🔾 | 🔾 | 🔾 | 🔾 |
| Pesticides | 🔾 | 🔾 | 🔾 | 🔾 | 🔾 | 🔾 | 🔾 | 🔾 | 🔾 | 🔾 | 🔾 | 🔾 | 🔾 |
| Herbicides | 🔾 | 🔾 | 🔾 | 🔾 | 🔾 | 🔾 | 🔾 | 🔾 | 🔾 | 🔾 | 🔾 | 🔾 | 🔾 |
| Fungicides | 🔾 | 🔾 | 🔾 | 🔾 | 🔾 | 🔾 | 🔾 | 🔾 | 🔾 | 🔾 | 🔾 | 🔾 | 🔾 |
| Dyes, printing inks | 🔾 | 🔾 | 🔾 | 🔾 | 🔾 | 🔾 | 🔾 | 🔾 | 🔾 | 🔾 | 🔾 | 🔾 | 🔾 |
| Adhesives | 🔾 | 🔾 | 🔾 | 🔾 | 🔾 | 🔾 | 🔾 | 🔾 | 🔾 | 🔾 | 🔾 | 🔾 | 🔾 |
| Paint, paint thinners, paint strippers, varnishes | 🔾 | 🔾 | 🔾 | 🔾 | 🔾 | 🔾 | 🔾 | 🔾 | 🔾 | 🔾 | 🔾 | 🔾 | 🔾 |
| Cutting, cooling, or lubricating oils. | 🔾 | 🔾 | 🔾 | 🔾 | 🔾 | 🔾 | 🔾 | 🔾 | 🔾 | 🔾 | 🔾 | 🔾 | 🔾 |
| Gasoline, diesel, aviation fuel | 🔾 | 🔾 | 🔾 | 🔾 | 🔾 | 🔾 | 🔾 | 🔾 | 🔾 | 🔾 | 🔾 | 🔾 | 🔾 |
| Antifreeze, refrigerants | 🔾 | 🔾 | 🔾 | 🔾 | 🔾 | 🔾 | 🔾 | 🔾 | 🔾 | 🔾 | 🔾 | 🔾 | 🔾 |
| Petrochemicals, plastics, monomers | 🔾 | 🔾 | 🔾 | 🔾 | 🔾 | 🔾 | 🔾 | 🔾 | 🔾 | 🔾 | 🔾 | 🔾 | 🔾 |
| Aromatic solvents, benzene, toluene, xylene, phenol | 🔾 | 🔾 | 🔾 | 🔾 | 🔾 | 🔾 | 🔾 | 🔾 | 🔾 | 🔾 | 🔾 | 🔾 | 🔾 |
| Automobile exhaust, dust, particulates | 🔾 | 🔾 | 🔾 | 🔾 | 🔾 | 🔾 | 🔾 | 🔾 | 🔾 | 🔾 | 🔾 | 🔾 | 🔾 |
| Soaps, detergents manufacturing | 🔾 | 🔾 | 🔾 | 🔾 | 🔾 | 🔾 | 🔾 | 🔾 | 🔾 | 🔾 | 🔾 | 🔾 | 🔾 |
| Fine chemicals, perfumes, drugs manufacture or dispensing | 🔾 | 🔾 | 🔾 | 🔾 | 🔾 | 🔾 | 🔾 | 🔾 | 🔾 | 🔾 | 🔾 | 🔾 | 🔾 |
| Purified biochemicals, enzymes | 🔾 | 🔾 | 🔾 | 🔾 | 🔾 | 🔾 | 🔾 | 🔾 | 🔾 | 🔾 | 🔾 | 🔾 | 🔾 |
| Inorganic acids, bases | 🔾 | 🔾 | 🔾 | 🔾 | 🔾 | 🔾 | 🔾 | 🔾 | 🔾 | 🔾 | 🔾 | 🔾 | 🔾 |
| Organic acids, bases | 🔾 | 🔾 | 🔾 | 🔾 | 🔾 | 🔾 | 🔾 | 🔾 | 🔾 | 🔾 | 🔾 | 🔾 | 🔾 |
| Solvents, degreasing agents | 🔾 | 🔾 | 🔾 | 🔾 | 🔾 | 🔾 | 🔾 | 🔾 | 🔾 | 🔾 | 🔾 | 🔾 | 🔾 |
| Dry cleaning agents | 🔾 | 🔾 | 🔾 | 🔾 | 🔾 | 🔾 | 🔾 | 🔾 | 🔾 | 🔾 | 🔾 | 🔾 | 🔾 |
| Nail salon products | 🔾 | 🔾 | 🔾 | 🔾 | 🔾 | 🔾 | 🔾 | 🔾 | 🔾 | 🔾 | 🔾 | 🔾 | 🔾 |
| Hairdressing products. | 🔾 | 🔾 | 🔾 | 🔾 | 🔾 | 🔾 | 🔾 | 🔾 | 🔾 | 🔾 | 🔾 | 🔾 | 🔾 |
| Building materials, cement, concrete. | 🔾 | 🔾 | 🔾 | 🔾 | 🔾 | 🔾 | 🔾 | 🔾 | 🔾 | 🔾 | 🔾 | 🔾 | 🔾 |
| Road, highway public works, asphalt, coal tar. | 🔾 | 🔾 | 🔾 | 🔾 | 🔾 | 🔾 | 🔾 | 🔾 | 🔾 | 🔾 | 🔾 | 🔾 | 🔾 |
| Mud, sand, stone, quarrying, farming | 🔾 | 🔾 | 🔾 | 🔾 | 🔾 | 🔾 | 🔾 | 🔾 | 🔾 | 🔾 | 🔾 | 🔾 | 🔾 |
| Forest, forestry, logging, saw mill | 🔾 | 🔾 | 🔾 | 🔾 | 🔾 | 🔾 | 🔾 | 🔾 | 🔾 | 🔾 | 🔾 | 🔾 | 🔾 |
| Intense heat: Fires, fire fighting, furnaces, ovens, kilns | 🔾 | 🔾 | 🔾 | 🔾 | 🔾 | 🔾 | 🔾 | 🔾 | 🔾 | 🔾 | 🔾 | 🔾 | 🔾 |
| Waste water, pond water, lake water, marshland, sewage | 🔾 | 🔾 | 🔾 | 🔾 | 🔾 | 🔾 | 🔾 | 🔾 | 🔾 | 🔾 | 🔾 | 🔾 | 🔾 |
| Other (specify) | 🔾 | 🔾 | 🔾 | 🔾 | 🔾 | 🔾 | 🔾 | 🔾 | 🔾 | 🔾 | 🔾 | 🔾 | 🔾 |
| Not applicable at this time | 🔾 | 🔾 | 🔾 | 🔾 | 🔾 | 🔾 | 🔾 | 🔾 | 🔾 | 🔾 | 🔾 | 🔾 | 🔾 |
| Declined | 🔾 | 🔾 | 🔾 | 🔾 | 🔾 | 🔾 | 🔾 | 🔾 | 🔾 | 🔾 | 🔾 | 🔾 | 🔾 |

|  | After Onset of MS | | | | | | | | | | | | |
| --- | --- | --- | --- | --- | --- | --- | --- | --- | --- | --- | --- | --- | --- |
|  | 0 to 3 months | 4 to 6 months | 7 months to 1 year | 1.01 to 2 years | 2.01 to 3 years | 3.01 to 5 years | 6 to 10 years | 11 to 15 years | 16 to 20 years | More than 20 years | Don't remember | Not applicable at this time | Declined |
| Lead, zinc, heavy metals | 🔾 | 🔾 | 🔾 | 🔾 | 🔾 | 🔾 | 🔾 | 🔾 | 🔾 | 🔾 | 🔾 | 🔾 | 🔾 |
| Mining, ores | 🔾 | 🔾 | 🔾 | 🔾 | 🔾 | 🔾 | 🔾 | 🔾 | 🔾 | 🔾 | 🔾 | 🔾 | 🔾 |
| Steelmaking, smelting, foundry operations | 🔾 | 🔾 | 🔾 | 🔾 | 🔾 | 🔾 | 🔾 | 🔾 | 🔾 | 🔾 | 🔾 | 🔾 | 🔾 |
| Coal, coal dust, coal boilers, coal fires | 🔾 | 🔾 | 🔾 | 🔾 | 🔾 | 🔾 | 🔾 | 🔾 | 🔾 | 🔾 | 🔾 | 🔾 | 🔾 |
| Pesticides | 🔾 | 🔾 | 🔾 | 🔾 | 🔾 | 🔾 | 🔾 | 🔾 | 🔾 | 🔾 | 🔾 | 🔾 | 🔾 |
| Herbicides | 🔾 | 🔾 | 🔾 | 🔾 | 🔾 | 🔾 | 🔾 | 🔾 | 🔾 | 🔾 | 🔾 | 🔾 | 🔾 |
| Fungicides | 🔾 | 🔾 | 🔾 | 🔾 | 🔾 | 🔾 | 🔾 | 🔾 | 🔾 | 🔾 | 🔾 | 🔾 | 🔾 |
| Dyes, printing inks | 🔾 | 🔾 | 🔾 | 🔾 | 🔾 | 🔾 | 🔾 | 🔾 | 🔾 | 🔾 | 🔾 | 🔾 | 🔾 |
| Adhesives | 🔾 | 🔾 | 🔾 | 🔾 | 🔾 | 🔾 | 🔾 | 🔾 | 🔾 | 🔾 | 🔾 | 🔾 | 🔾 |
| Paint, paint thinners, paint strippers, varnishes | 🔾 | 🔾 | 🔾 | 🔾 | 🔾 | 🔾 | 🔾 | 🔾 | 🔾 | 🔾 | 🔾 | 🔾 | 🔾 |
| Cutting, cooling, or lubricating oils. | 🔾 | 🔾 | 🔾 | 🔾 | 🔾 | 🔾 | 🔾 | 🔾 | 🔾 | 🔾 | 🔾 | 🔾 | 🔾 |
| Gasoline, diesel, aviation fuel | 🔾 | 🔾 | 🔾 | 🔾 | 🔾 | 🔾 | 🔾 | 🔾 | 🔾 | 🔾 | 🔾 | 🔾 | 🔾 |
| Antifreeze, refrigerants | 🔾 | 🔾 | 🔾 | 🔾 | 🔾 | 🔾 | 🔾 | 🔾 | 🔾 | 🔾 | 🔾 | 🔾 | 🔾 |
| Petrochemicals, plastics, monomers | 🔾 | 🔾 | 🔾 | 🔾 | 🔾 | 🔾 | 🔾 | 🔾 | 🔾 | 🔾 | 🔾 | 🔾 | 🔾 |
| Aromatic solvents, benzene, toluene, xylene, phenol | 🔾 | 🔾 | 🔾 | 🔾 | 🔾 | 🔾 | 🔾 | 🔾 | 🔾 | 🔾 | 🔾 | 🔾 | 🔾 |
| Automobile exhaust, dust, particulates | 🔾 | 🔾 | 🔾 | 🔾 | 🔾 | 🔾 | 🔾 | 🔾 | 🔾 | 🔾 | 🔾 | 🔾 | 🔾 |
| Soaps, detergents manufacturing | 🔾 | 🔾 | 🔾 | 🔾 | 🔾 | 🔾 | 🔾 | 🔾 | 🔾 | 🔾 | 🔾 | 🔾 | 🔾 |
| Fine chemicals, perfumes, drugs manufacture or dispensing | 🔾 | 🔾 | 🔾 | 🔾 | 🔾 | 🔾 | 🔾 | 🔾 | 🔾 | 🔾 | 🔾 | 🔾 | 🔾 |
| Purified biochemicals, enzymes | 🔾 | 🔾 | 🔾 | 🔾 | 🔾 | 🔾 | 🔾 | 🔾 | 🔾 | 🔾 | 🔾 | 🔾 | 🔾 |
| Inorganic acids, bases | 🔾 | 🔾 | 🔾 | 🔾 | 🔾 | 🔾 | 🔾 | 🔾 | 🔾 | 🔾 | 🔾 | 🔾 | 🔾 |
| Organic acids, bases | 🔾 | 🔾 | 🔾 | 🔾 | 🔾 | 🔾 | 🔾 | 🔾 | 🔾 | 🔾 | 🔾 | 🔾 | 🔾 |
| Solvents, degreasing agents | 🔾 | 🔾 | 🔾 | 🔾 | 🔾 | 🔾 | 🔾 | 🔾 | 🔾 | 🔾 | 🔾 | 🔾 | 🔾 |
| Dry cleaning agents | 🔾 | 🔾 | 🔾 | 🔾 | 🔾 | 🔾 | 🔾 | 🔾 | 🔾 | 🔾 | 🔾 | 🔾 | 🔾 |
| Nail salon products | 🔾 | 🔾 | 🔾 | 🔾 | 🔾 | 🔾 | 🔾 | 🔾 | 🔾 | 🔾 | 🔾 | 🔾 | 🔾 |
| Hairdressing products. | 🔾 | 🔾 | 🔾 | 🔾 | 🔾 | 🔾 | 🔾 | 🔾 | 🔾 | 🔾 | 🔾 | 🔾 | 🔾 |
| Building materials, cement, concrete. | 🔾 | 🔾 | 🔾 | 🔾 | 🔾 | 🔾 | 🔾 | 🔾 | 🔾 | 🔾 | 🔾 | 🔾 | 🔾 |
| Road, highway public works, asphalt, coal tar. | 🔾 | 🔾 | 🔾 | 🔾 | 🔾 | 🔾 | 🔾 | 🔾 | 🔾 | 🔾 | 🔾 | 🔾 | 🔾 |
| Mud, sand, stone, quarrying, farming | 🔾 | 🔾 | 🔾 | 🔾 | 🔾 | 🔾 | 🔾 | 🔾 | 🔾 | 🔾 | 🔾 | 🔾 | 🔾 |
| Forest, forestry, logging, saw mill | 🔾 | 🔾 | 🔾 | 🔾 | 🔾 | 🔾 | 🔾 | 🔾 | 🔾 | 🔾 | 🔾 | 🔾 | 🔾 |
| Intense heat: Fires, fire fighting, furnaces, ovens, kilns | 🔾 | 🔾 | 🔾 | 🔾 | 🔾 | 🔾 | 🔾 | 🔾 | 🔾 | 🔾 | 🔾 | 🔾 | 🔾 |
| Waste water, pond water, lake water, marshland, sewage | 🔾 | 🔾 | 🔾 | 🔾 | 🔾 | 🔾 | 🔾 | 🔾 | 🔾 | 🔾 | 🔾 | 🔾 | 🔾 |
| Other (specify) | 🔾 | 🔾 | 🔾 | 🔾 | 🔾 | 🔾 | 🔾 | 🔾 | 🔾 | 🔾 | 🔾 | 🔾 | 🔾 |
| Not applicable at this time | 🔾 | 🔾 | 🔾 | 🔾 | 🔾 | 🔾 | 🔾 | 🔾 | 🔾 | 🔾 | 🔾 | 🔾 | 🔾 |
| Declined | 🔾 | 🔾 | 🔾 | 🔾 | 🔾 | 🔾 | 🔾 | 🔾 | 🔾 | 🔾 | 🔾 | 🔾 | 🔾 |

**145) Comments regarding Chemical or Extreme Physical Condition.**

____________________________________________________________

**146) Dietary Restraints. Indicate the Types of Foods that You do Not Eat or the Dietary Restraints you follow. Check all the relevant boxes.**

|  | Non-MS Subject or Before Onset of MS | | | | | | |
| --- | --- | --- | --- | --- | --- | --- | --- |
|  | I Do NOT Eat this type of food | I Eat this type of food | Don’t Remember | Not Applicable | Not Applicable at this stage | Other | Declined |
| Meat | 🔾 | 🔾 | 🔾 | 🔾 | 🔾 | 🔾 | 🔾 |
| Pork | 🔾 | 🔾 | 🔾 | 🔾 | 🔾 | 🔾 | 🔾 |
| Beef | 🔾 | 🔾 | 🔾 | 🔾 | 🔾 | 🔾 | 🔾 |
| Chicken, Turkey, Poultry etc. | 🔾 | 🔾 | 🔾 | 🔾 | 🔾 | 🔾 | 🔾 |
| Fish | 🔾 | 🔾 | 🔾 | 🔾 | 🔾 | 🔾 | 🔾 |
| Shrimp, Lobster etc | 🔾 | 🔾 | 🔾 | 🔾 | 🔾 | 🔾 | 🔾 |
| Shellfish, Oysters etc | 🔾 | 🔾 | 🔾 | 🔾 | 🔾 | 🔾 | 🔾 |
| Eggs | 🔾 | 🔾 | 🔾 | 🔾 | 🔾 | 🔾 | 🔾 |
| Milk, Dairy | 🔾 | 🔾 | 🔾 | 🔾 | 🔾 | 🔾 | 🔾 |
| Exclusively Vegetarian Diet with Dairy, with Eggs | 🔾 | 🔾 | 🔾 | 🔾 | 🔾 | 🔾 | 🔾 |
| Exclusively Vegetarian Diet with Dairy, No Eggs | 🔾 | 🔾 | 🔾 | 🔾 | 🔾 | 🔾 | 🔾 |
| Exclusively Vegan Diet, No Dairy, No Eggs | 🔾 | 🔾 | 🔾 | 🔾 | 🔾 | 🔾 | 🔾 |
| Exclusively Kosher Diet | 🔾 | 🔾 | 🔾 | 🔾 | 🔾 | 🔾 | 🔾 |
| Other (specify) | 🔾 | 🔾 | 🔾 | 🔾 | 🔾 | 🔾 | 🔾 |

|  | After Onset of MS | | | | | | |
| --- | --- | --- | --- | --- | --- | --- | --- |
|  | I Do NOT Eat this type of food | I Eat this type of food | Don’t Remember | Not Applicable | Not Applicable at this stage | Other | Declined |
| Meat | 🔾 | 🔾 | 🔾 | 🔾 | 🔾 | 🔾 | 🔾 |
| Pork | 🔾 | 🔾 | 🔾 | 🔾 | 🔾 | 🔾 | 🔾 |
| Beef | 🔾 | 🔾 | 🔾 | 🔾 | 🔾 | 🔾 | 🔾 |
| Chicken, Turkey, Poultry etc. | 🔾 | 🔾 | 🔾 | 🔾 | 🔾 | 🔾 | 🔾 |
| Fish | 🔾 | 🔾 | 🔾 | 🔾 | 🔾 | 🔾 | 🔾 |
| Shrimp, Lobster etc | 🔾 | 🔾 | 🔾 | 🔾 | 🔾 | 🔾 | 🔾 |
| Shellfish, Oysters etc | 🔾 | 🔾 | 🔾 | 🔾 | 🔾 | 🔾 | 🔾 |
| Eggs | 🔾 | 🔾 | 🔾 | 🔾 | 🔾 | 🔾 | 🔾 |
| Milk, Dairy | 🔾 | 🔾 | 🔾 | 🔾 | 🔾 | 🔾 | 🔾 |
| Exclusively Vegetarian Diet with Dairy, with Eggs | 🔾 | 🔾 | 🔾 | 🔾 | 🔾 | 🔾 | 🔾 |
| Exclusively Vegetarian Diet with Dairy, No Eggs | 🔾 | 🔾 | 🔾 | 🔾 | 🔾 | 🔾 | 🔾 |
| Exclusively Vegan Diet, No Dairy, No Eggs | 🔾 | 🔾 | 🔾 | 🔾 | 🔾 | 🔾 | 🔾 |
| Exclusively Kosher Diet | 🔾 | 🔾 | 🔾 | 🔾 | 🔾 | 🔾 | 🔾 |
| Other (specify) | 🔾 | 🔾 | 🔾 | 🔾 | 🔾 | 🔾 | 🔾 |

|  | Last 3-Months? | | | | | | |
| --- | --- | --- | --- | --- | --- | --- | --- |
|  | I Do NOT Eat this type of food | I Eat this type of food | Don’t Remember | Not Applicable | Not Applicable at this stage | Other | Declined |
| Meat | 🔾 | 🔾 | 🔾 | 🔾 | 🔾 | 🔾 | 🔾 |
| Pork | 🔾 | 🔾 | 🔾 | 🔾 | 🔾 | 🔾 | 🔾 |
| Beef | 🔾 | 🔾 | 🔾 | 🔾 | 🔾 | 🔾 | 🔾 |
| Chicken, Turkey, Poultry etc. | 🔾 | 🔾 | 🔾 | 🔾 | 🔾 | 🔾 | 🔾 |
| Fish | 🔾 | 🔾 | 🔾 | 🔾 | 🔾 | 🔾 | 🔾 |
| Shrimp, Lobster etc | 🔾 | 🔾 | 🔾 | 🔾 | 🔾 | 🔾 | 🔾 |
| Shellfish, Oysters etc | 🔾 | 🔾 | 🔾 | 🔾 | 🔾 | 🔾 | 🔾 |
| Eggs | 🔾 | 🔾 | 🔾 | 🔾 | 🔾 | 🔾 | 🔾 |
| Milk, Dairy | 🔾 | 🔾 | 🔾 | 🔾 | 🔾 | 🔾 | 🔾 |
| Exclusively Vegetarian Diet with Dairy, with Eggs | 🔾 | 🔾 | 🔾 | 🔾 | 🔾 | 🔾 | 🔾 |
| Exclusively Vegetarian Diet with Dairy, No Eggs | 🔾 | 🔾 | 🔾 | 🔾 | 🔾 | 🔾 | 🔾 |
| Exclusively Vegan Diet, No Dairy, No Eggs | 🔾 | 🔾 | 🔾 | 🔾 | 🔾 | 🔾 | 🔾 |
| Exclusively Kosher Diet | 🔾 | 🔾 | 🔾 | 🔾 | 🔾 | 🔾 | 🔾 |
| Other (specify) | 🔾 | 🔾 | 🔾 | 🔾 | 🔾 | 🔾 | 🔾 |

**147) Please Summarize Your Overall Eating Patterns. Include breakfast, lunch and dinner.**

|  | Non-MS Subject or Before Onset of MS | | | | | | | | | | | | |
| --- | --- | --- | --- | --- | --- | --- | --- | --- | --- | --- | --- | --- | --- |
|  | Never | Less than once a month | 1-3 times a month | Once a week | 2-4 times a week | 5-6 times a week | Once a day | 2-3 times a day | 4 or more times a day | Don’t Remember | Not Applicable | Not Applicable at this stage | Other (specify) |
| Any kind of meat? | 🔾 | 🔾 | 🔾 | 🔾 | 🔾 | 🔾 | 🔾 | 🔾 | 🔾 | 🔾 | 🔾 | 🔾 | 🔾 |
| Any kind of fish, tuna, seafood? | 🔾 | 🔾 | 🔾 | 🔾 | 🔾 | 🔾 | 🔾 | 🔾 | 🔾 | 🔾 | 🔾 | 🔾 | 🔾 |
| Any kind of Vegetables? | 🔾 | 🔾 | 🔾 | 🔾 | 🔾 | 🔾 | 🔾 | 🔾 | 🔾 | 🔾 | 🔾 | 🔾 | 🔾 |
| Any kind of Raw Vegetable, e.g., salads, carrots etc.? | 🔾 | 🔾 | 🔾 | 🔾 | 🔾 | 🔾 | 🔾 | 🔾 | 🔾 | 🔾 | 🔾 | 🔾 | 🔾 |
| Any kind of Fresh Fruit? | 🔾 | 🔾 | 🔾 | 🔾 | 🔾 | 🔾 | 🔾 | 🔾 | 🔾 | 🔾 | 🔾 | 🔾 | 🔾 |
| Any kind of Fruit Juice, e.g., OJ | 🔾 | 🔾 | 🔾 | 🔾 | 🔾 | 🔾 | 🔾 | 🔾 | 🔾 | 🔾 | 🔾 | 🔾 | 🔾 |
| Any kind of Nuts, e.g., Peanuts, Cashews, Almonds? | 🔾 | 🔾 | 🔾 | 🔾 | 🔾 | 🔾 | 🔾 | 🔾 | 🔾 | 🔾 | 🔾 | 🔾 | 🔾 |

|  | After Onset of MS | | | | | | | | | | | | |
| --- | --- | --- | --- | --- | --- | --- | --- | --- | --- | --- | --- | --- | --- |
|  | Never | Less than once a month | 1-3 times a month | Once a week | 2-4 times a week | 5-6 times a week | Once a day | 2-3 times a day | 4 or more times a day | Don’t Remember | Not Applicable | Not Applicable at this stage | Other (specify) |
| Any kind of meat? | 🔾 | 🔾 | 🔾 | 🔾 | 🔾 | 🔾 | 🔾 | 🔾 | 🔾 | 🔾 | 🔾 | 🔾 | 🔾 |
| Any kind of fish, tuna, seafood? | 🔾 | 🔾 | 🔾 | 🔾 | 🔾 | 🔾 | 🔾 | 🔾 | 🔾 | 🔾 | 🔾 | 🔾 | 🔾 |
| Any kind of Vegetables? | 🔾 | 🔾 | 🔾 | 🔾 | 🔾 | 🔾 | 🔾 | 🔾 | 🔾 | 🔾 | 🔾 | 🔾 | 🔾 |
| Any kind of Raw Vegetable, e.g., salads, carrots etc.? | 🔾 | 🔾 | 🔾 | 🔾 | 🔾 | 🔾 | 🔾 | 🔾 | 🔾 | 🔾 | 🔾 | 🔾 | 🔾 |
| Any kind of Fresh Fruit? | 🔾 | 🔾 | 🔾 | 🔾 | 🔾 | 🔾 | 🔾 | 🔾 | 🔾 | 🔾 | 🔾 | 🔾 | 🔾 |
| Any kind of Fruit Juice, e.g., OJ | 🔾 | 🔾 | 🔾 | 🔾 | 🔾 | 🔾 | 🔾 | 🔾 | 🔾 | 🔾 | 🔾 | 🔾 | 🔾 |
| Any kind of Nuts, e.g., Peanuts, Cashews, Almonds? | 🔾 | 🔾 | 🔾 | 🔾 | 🔾 | 🔾 | 🔾 | 🔾 | 🔾 | 🔾 | 🔾 | 🔾 | 🔾 |

|  | Last 3 Months? | | | | | | | | | | | | |
| --- | --- | --- | --- | --- | --- | --- | --- | --- | --- | --- | --- | --- | --- |
|  | Never | Less than once a month | 1-3 times a month | Once a week | 2-4 times a week | 5-6 times a week | Once a day | 2-3 times a day | 4 or more times a day | Don’t Remember | Not Applicable | Not Applicable at this stage | Other (specify) |
| Any kind of meat? | 🔾 | 🔾 | 🔾 | 🔾 | 🔾 | 🔾 | 🔾 | 🔾 | 🔾 | 🔾 | 🔾 | 🔾 | 🔾 |
| Any kind of fish, tuna, seafood? | 🔾 | 🔾 | 🔾 | 🔾 | 🔾 | 🔾 | 🔾 | 🔾 | 🔾 | 🔾 | 🔾 | 🔾 | 🔾 |
| Any kind of Vegetables? | 🔾 | 🔾 | 🔾 | 🔾 | 🔾 | 🔾 | 🔾 | 🔾 | 🔾 | 🔾 | 🔾 | 🔾 | 🔾 |
| Any kind of Raw Vegetable, e.g., salads, carrots etc.? | 🔾 | 🔾 | 🔾 | 🔾 | 🔾 | 🔾 | 🔾 | 🔾 | 🔾 | 🔾 | 🔾 | 🔾 | 🔾 |
| Any kind of Fresh Fruit? | 🔾 | 🔾 | 🔾 | 🔾 | 🔾 | 🔾 | 🔾 | 🔾 | 🔾 | 🔾 | 🔾 | 🔾 | 🔾 |
| Any kind of Fruit Juice, e.g., OJ | 🔾 | 🔾 | 🔾 | 🔾 | 🔾 | 🔾 | 🔾 | 🔾 | 🔾 | 🔾 | 🔾 | 🔾 | 🔾 |
| Any kind of Nuts, e.g., Peanuts, Cashews, Almonds? | 🔾 | 🔾 | 🔾 | 🔾 | 🔾 | 🔾 | 🔾 | 🔾 | 🔾 | 🔾 | 🔾 | 🔾 | 🔾 |

**148) Comments Regarding Overall Eating Patterns** _______________________________________________

**149) Oils and Fats: What Oils and Fats were Commonly Used in Your Household for Cooking, Frying, Sauteeing etc.?**

|  | Non-MS Subject or Before Onset of MS | | | | | | | | | | | | | |
| --- | --- | --- | --- | --- | --- | --- | --- | --- | --- | --- | --- | --- | --- | --- |
|  | Never | Less than once a month | 1-3 times a month | Once a week | 2-4 times a week | 5-6 times a week | Once a day | 2-3 times a day | 4 or more times a day | Don’t Remember | Not Applicable | Not Applicable at this stage | Other (specify) | Declined |
| Peanut Oil | 🔾 | 🔾 | 🔾 | 🔾 | 🔾 | 🔾 | 🔾 | 🔾 | 🔾 | 🔾 | 🔾 | 🔾 | 🔾 | 🔾 |
| Sesame Oil | 🔾 | 🔾 | 🔾 | 🔾 | 🔾 | 🔾 | 🔾 | 🔾 | 🔾 | 🔾 | 🔾 | 🔾 | 🔾 | 🔾 |
| Butter | 🔾 | 🔾 | 🔾 | 🔾 | 🔾 | 🔾 | 🔾 | 🔾 | 🔾 | 🔾 | 🔾 | 🔾 | 🔾 | 🔾 |
| Coconut Oil | 🔾 | 🔾 | 🔾 | 🔾 | 🔾 | 🔾 | 🔾 | 🔾 | 🔾 | 🔾 | 🔾 | 🔾 | 🔾 | 🔾 |
| Clarified Butter, Ghee | 🔾 | 🔾 | 🔾 | 🔾 | 🔾 | 🔾 | 🔾 | 🔾 | 🔾 | 🔾 | 🔾 | 🔾 | 🔾 | 🔾 |
| Olive Oil | 🔾 | 🔾 | 🔾 | 🔾 | 🔾 | 🔾 | 🔾 | 🔾 | 🔾 | 🔾 | 🔾 | 🔾 | 🔾 | 🔾 |
| Corn Oil | 🔾 | 🔾 | 🔾 | 🔾 | 🔾 | 🔾 | 🔾 | 🔾 | 🔾 | 🔾 | 🔾 | 🔾 | 🔾 | 🔾 |
| Sunflower Oil | 🔾 | 🔾 | 🔾 | 🔾 | 🔾 | 🔾 | 🔾 | 🔾 | 🔾 | 🔾 | 🔾 | 🔾 | 🔾 | 🔾 |
| Lard | 🔾 | 🔾 | 🔾 | 🔾 | 🔾 | 🔾 | 🔾 | 🔾 | 🔾 | 🔾 | 🔾 | 🔾 | 🔾 | 🔾 |
| Fish Oil | 🔾 | 🔾 | 🔾 | 🔾 | 🔾 | 🔾 | 🔾 | 🔾 | 🔾 | 🔾 | 🔾 | 🔾 | 🔾 | 🔾 |
| Mustard or Rapeseed Oild | 🔾 | 🔾 | 🔾 | 🔾 | 🔾 | 🔾 | 🔾 | 🔾 | 🔾 | 🔾 | 🔾 | 🔾 | 🔾 | 🔾 |
| Other (Specify) | 🔾 | 🔾 | 🔾 | 🔾 | 🔾 | 🔾 | 🔾 | 🔾 | 🔾 | 🔾 | 🔾 | 🔾 | 🔾 | 🔾 |

|  | After Onset of MS | | | | | | | | | | | | | |
| --- | --- | --- | --- | --- | --- | --- | --- | --- | --- | --- | --- | --- | --- | --- |
|  | Never | Less than once a month | 1-3 times a month | Once a week | 2-4 times a week | 5-6 times a week | Once a day | 2-3 times a day | 4 or more times a day | Don’t Remember | Not Applicable | Not Applicable at this stage | Other (specify) | Declined |
| Peanut Oil | 🔾 | 🔾 | 🔾 | 🔾 | 🔾 | 🔾 | 🔾 | 🔾 | 🔾 | 🔾 | 🔾 | 🔾 | 🔾 | 🔾 |
| Sesame Oil | 🔾 | 🔾 | 🔾 | 🔾 | 🔾 | 🔾 | 🔾 | 🔾 | 🔾 | 🔾 | 🔾 | 🔾 | 🔾 | 🔾 |
| Butter | 🔾 | 🔾 | 🔾 | 🔾 | 🔾 | 🔾 | 🔾 | 🔾 | 🔾 | 🔾 | 🔾 | 🔾 | 🔾 | 🔾 |
| Coconut Oil | 🔾 | 🔾 | 🔾 | 🔾 | 🔾 | 🔾 | 🔾 | 🔾 | 🔾 | 🔾 | 🔾 | 🔾 | 🔾 | 🔾 |
| Clarified Butter, Ghee | 🔾 | 🔾 | 🔾 | 🔾 | 🔾 | 🔾 | 🔾 | 🔾 | 🔾 | 🔾 | 🔾 | 🔾 | 🔾 | 🔾 |
| Olive Oil | 🔾 | 🔾 | 🔾 | 🔾 | 🔾 | 🔾 | 🔾 | 🔾 | 🔾 | 🔾 | 🔾 | 🔾 | 🔾 | 🔾 |
| Corn Oil | 🔾 | 🔾 | 🔾 | 🔾 | 🔾 | 🔾 | 🔾 | 🔾 | 🔾 | 🔾 | 🔾 | 🔾 | 🔾 | 🔾 |
| Sunflower Oil | 🔾 | 🔾 | 🔾 | 🔾 | 🔾 | 🔾 | 🔾 | 🔾 | 🔾 | 🔾 | 🔾 | 🔾 | 🔾 | 🔾 |
| Lard | 🔾 | 🔾 | 🔾 | 🔾 | 🔾 | 🔾 | 🔾 | 🔾 | 🔾 | 🔾 | 🔾 | 🔾 | 🔾 | 🔾 |
| Fish Oil | 🔾 | 🔾 | 🔾 | 🔾 | 🔾 | 🔾 | 🔾 | 🔾 | 🔾 | 🔾 | 🔾 | 🔾 | 🔾 | 🔾 |
| Mustard or Rapeseed Oild | 🔾 | 🔾 | 🔾 | 🔾 | 🔾 | 🔾 | 🔾 | 🔾 | 🔾 | 🔾 | 🔾 | 🔾 | 🔾 | 🔾 |
| Other (Specify) | 🔾 | 🔾 | 🔾 | 🔾 | 🔾 | 🔾 | 🔾 | 🔾 | 🔾 | 🔾 | 🔾 | 🔾 | 🔾 | 🔾 |

|  | Last 3-Months? | | | | | | | | | | | | | |
| --- | --- | --- | --- | --- | --- | --- | --- | --- | --- | --- | --- | --- | --- | --- |
|  | Never | Less than once a month | 1-3 times a month | Once a week | 2-4 times a week | 5-6 times a week | Once a day | 2-3 times a day | 4 or more times a day | Don’t Remember | Not Applicable | Not Applicable at this stage | Other (specify) | Declined |
| Peanut Oil | 🔾 | 🔾 | 🔾 | 🔾 | 🔾 | 🔾 | 🔾 | 🔾 | 🔾 | 🔾 | 🔾 | 🔾 | 🔾 | 🔾 |
| Sesame Oil | 🔾 | 🔾 | 🔾 | 🔾 | 🔾 | 🔾 | 🔾 | 🔾 | 🔾 | 🔾 | 🔾 | 🔾 | 🔾 | 🔾 |
| Butter | 🔾 | 🔾 | 🔾 | 🔾 | 🔾 | 🔾 | 🔾 | 🔾 | 🔾 | 🔾 | 🔾 | 🔾 | 🔾 | 🔾 |
| Coconut Oil | 🔾 | 🔾 | 🔾 | 🔾 | 🔾 | 🔾 | 🔾 | 🔾 | 🔾 | 🔾 | 🔾 | 🔾 | 🔾 | 🔾 |
| Clarified Butter, Ghee | 🔾 | 🔾 | 🔾 | 🔾 | 🔾 | 🔾 | 🔾 | 🔾 | 🔾 | 🔾 | 🔾 | 🔾 | 🔾 | 🔾 |
| Olive Oil | 🔾 | 🔾 | 🔾 | 🔾 | 🔾 | 🔾 | 🔾 | 🔾 | 🔾 | 🔾 | 🔾 | 🔾 | 🔾 | 🔾 |
| Corn Oil | 🔾 | 🔾 | 🔾 | 🔾 | 🔾 | 🔾 | 🔾 | 🔾 | 🔾 | 🔾 | 🔾 | 🔾 | 🔾 | 🔾 |
| Sunflower Oil | 🔾 | 🔾 | 🔾 | 🔾 | 🔾 | 🔾 | 🔾 | 🔾 | 🔾 | 🔾 | 🔾 | 🔾 | 🔾 | 🔾 |
| Lard | 🔾 | 🔾 | 🔾 | 🔾 | 🔾 | 🔾 | 🔾 | 🔾 | 🔾 | 🔾 | 🔾 | 🔾 | 🔾 | 🔾 |
| Fish Oil | 🔾 | 🔾 | 🔾 | 🔾 | 🔾 | 🔾 | 🔾 | 🔾 | 🔾 | 🔾 | 🔾 | 🔾 | 🔾 | 🔾 |
| Mustard or Rapeseed Oild | 🔾 | 🔾 | 🔾 | 🔾 | 🔾 | 🔾 | 🔾 | 🔾 | 🔾 | 🔾 | 🔾 | 🔾 | 🔾 | 🔾 |
| Other (Specify) | 🔾 | 🔾 | 🔾 | 🔾 | 🔾 | 🔾 | 🔾 | 🔾 | 🔾 | 🔾 | 🔾 | 🔾 | 🔾 | 🔾 |

**150) Comments Regarding Oils and Fats** ________________________________________________________

**151) Protein Sources: What Protein Sources were Commonly Used in Your Household? Include Breakfast Protein Sources such as Bacon, Eggs and also Meat in Lunch Sandwiches. Give the best estimates you can.**

|  | Non-MS Subject or Before Onset of MS | | | | | | | | | | | | | |
| --- | --- | --- | --- | --- | --- | --- | --- | --- | --- | --- | --- | --- | --- | --- |
|  | Never | Less than once a month | 1-3 times a month | Once a week | 2-4 times a week | 5-6 times a week | Once a day | 2-3 times a day | 4 or more times a day | Don’t Remember | Not Applicable | Not Applicable at this stage | Other (specify) | Declined |
| Eggs in any form, Boiled, Omelettes, Scrambled | 🔾 | 🔾 | 🔾 | 🔾 | 🔾 | 🔾 | 🔾 | 🔾 | 🔾 | 🔾 | 🔾 | 🔾 | 🔾 | 🔾 |
| Beef, Beef Products | 🔾 | 🔾 | 🔾 | 🔾 | 🔾 | 🔾 | 🔾 | 🔾 | 🔾 | 🔾 | 🔾 | 🔾 | 🔾 | 🔾 |
| Pork, Pork Products | 🔾 | 🔾 | 🔾 | 🔾 | 🔾 | 🔾 | 🔾 | 🔾 | 🔾 | 🔾 | 🔾 | 🔾 | 🔾 | 🔾 |
| Lamb, Mutton | 🔾 | 🔾 | 🔾 | 🔾 | 🔾 | 🔾 | 🔾 | 🔾 | 🔾 | 🔾 | 🔾 | 🔾 | 🔾 | 🔾 |
| Deer, Caribou, Reindeer | 🔾 | 🔾 | 🔾 | 🔾 | 🔾 | 🔾 | 🔾 | 🔾 | 🔾 | 🔾 | 🔾 | 🔾 | 🔾 | 🔾 |
| Chicken | 🔾 | 🔾 | 🔾 | 🔾 | 🔾 | 🔾 | 🔾 | 🔾 | 🔾 | 🔾 | 🔾 | 🔾 | 🔾 | 🔾 |
| Turkey, Duck, Other Poultry | 🔾 | 🔾 | 🔾 | 🔾 | 🔾 | 🔾 | 🔾 | 🔾 | 🔾 | 🔾 | 🔾 | 🔾 | 🔾 | 🔾 |
| Canned Tuna | 🔾 | 🔾 | 🔾 | 🔾 | 🔾 | 🔾 | 🔾 | 🔾 | 🔾 | 🔾 | 🔾 | 🔾 | 🔾 | 🔾 |
| Fish | 🔾 | 🔾 | 🔾 | 🔾 | 🔾 | 🔾 | 🔾 | 🔾 | 🔾 | 🔾 | 🔾 | 🔾 | 🔾 | 🔾 |
| Shrimp, Lobster | 🔾 | 🔾 | 🔾 | 🔾 | 🔾 | 🔾 | 🔾 | 🔾 | 🔾 | 🔾 | 🔾 | 🔾 | 🔾 | 🔾 |
| Oysters, Shellfish, Scallops | 🔾 | 🔾 | 🔾 | 🔾 | 🔾 | 🔾 | 🔾 | 🔾 | 🔾 | 🔾 | 🔾 | 🔾 | 🔾 | 🔾 |
| Beans | 🔾 | 🔾 | 🔾 | 🔾 | 🔾 | 🔾 | 🔾 | 🔾 | 🔾 | 🔾 | 🔾 | 🔾 | 🔾 | 🔾 |
| Lentils | 🔾 | 🔾 | 🔾 | 🔾 | 🔾 | 🔾 | 🔾 | 🔾 | 🔾 | 🔾 | 🔾 | 🔾 | 🔾 | 🔾 |
| Other (Specify) | 🔾 | 🔾 | 🔾 | 🔾 | 🔾 | 🔾 | 🔾 | 🔾 | 🔾 | 🔾 | 🔾 | 🔾 | 🔾 | 🔾 |

|  | After Onset of MS | | | | | | | | | | | | | |
| --- | --- | --- | --- | --- | --- | --- | --- | --- | --- | --- | --- | --- | --- | --- |
|  | Never | Less than once a month | 1-3 times a month | Once a week | 2-4 times a week | 5-6 times a week | Once a day | 2-3 times a day | 4 or more times a day | Don’t Remember | Not Applicable | Not Applicable at this stage | Other (specify) | Declined |
| Eggs in any form, Boiled, Omelettes, Scrambled | 🔾 | 🔾 | 🔾 | 🔾 | 🔾 | 🔾 | 🔾 | 🔾 | 🔾 | 🔾 | 🔾 | 🔾 | 🔾 | 🔾 |
| Beef, Beef Products | 🔾 | 🔾 | 🔾 | 🔾 | 🔾 | 🔾 | 🔾 | 🔾 | 🔾 | 🔾 | 🔾 | 🔾 | 🔾 | 🔾 |
| Pork, Pork Products | 🔾 | 🔾 | 🔾 | 🔾 | 🔾 | 🔾 | 🔾 | 🔾 | 🔾 | 🔾 | 🔾 | 🔾 | 🔾 | 🔾 |
| Lamb, Mutton | 🔾 | 🔾 | 🔾 | 🔾 | 🔾 | 🔾 | 🔾 | 🔾 | 🔾 | 🔾 | 🔾 | 🔾 | 🔾 | 🔾 |
| Deer, Caribou, Reindeer | 🔾 | 🔾 | 🔾 | 🔾 | 🔾 | 🔾 | 🔾 | 🔾 | 🔾 | 🔾 | 🔾 | 🔾 | 🔾 | 🔾 |
| Chicken | 🔾 | 🔾 | 🔾 | 🔾 | 🔾 | 🔾 | 🔾 | 🔾 | 🔾 | 🔾 | 🔾 | 🔾 | 🔾 | 🔾 |
| Turkey, Duck, Other Poultry | 🔾 | 🔾 | 🔾 | 🔾 | 🔾 | 🔾 | 🔾 | 🔾 | 🔾 | 🔾 | 🔾 | 🔾 | 🔾 | 🔾 |
| Canned Tuna | 🔾 | 🔾 | 🔾 | 🔾 | 🔾 | 🔾 | 🔾 | 🔾 | 🔾 | 🔾 | 🔾 | 🔾 | 🔾 | 🔾 |
| Fish | 🔾 | 🔾 | 🔾 | 🔾 | 🔾 | 🔾 | 🔾 | 🔾 | 🔾 | 🔾 | 🔾 | 🔾 | 🔾 | 🔾 |
| Shrimp, Lobster | 🔾 | 🔾 | 🔾 | 🔾 | 🔾 | 🔾 | 🔾 | 🔾 | 🔾 | 🔾 | 🔾 | 🔾 | 🔾 | 🔾 |
| Oysters, Shellfish, Scallops | 🔾 | 🔾 | 🔾 | 🔾 | 🔾 | 🔾 | 🔾 | 🔾 | 🔾 | 🔾 | 🔾 | 🔾 | 🔾 | 🔾 |
| Beans | 🔾 | 🔾 | 🔾 | 🔾 | 🔾 | 🔾 | 🔾 | 🔾 | 🔾 | 🔾 | 🔾 | 🔾 | 🔾 | 🔾 |
| Lentils | 🔾 | 🔾 | 🔾 | 🔾 | 🔾 | 🔾 | 🔾 | 🔾 | 🔾 | 🔾 | 🔾 | 🔾 | 🔾 | 🔾 |
| Other Meat (Specify) | 🔾 | 🔾 | 🔾 | 🔾 | 🔾 | 🔾 | 🔾 | 🔾 | 🔾 | 🔾 | 🔾 | 🔾 | 🔾 | 🔾 |

|  | Last 3-Months? | | | | | | | | | | | | | |
| --- | --- | --- | --- | --- | --- | --- | --- | --- | --- | --- | --- | --- | --- | --- |
|  | Never | Less than once a month | 1-3 times a month | Once a week | 2-4 times a week | 5-6 times a week | Once a day | 2-3 times a day | 4 or more times a day | Don’t Remember | Not Applicable | Not Applicable at this stage | Other (specify) | Declined |
| Eggs in any form, Boiled, Omelettes, Scrambled | 🔾 | 🔾 | 🔾 | 🔾 | 🔾 | 🔾 | 🔾 | 🔾 | 🔾 | 🔾 | 🔾 | 🔾 | 🔾 | 🔾 |
| Beef, Beef Products | 🔾 | 🔾 | 🔾 | 🔾 | 🔾 | 🔾 | 🔾 | 🔾 | 🔾 | 🔾 | 🔾 | 🔾 | 🔾 | 🔾 |
| Pork, Pork Products | 🔾 | 🔾 | 🔾 | 🔾 | 🔾 | 🔾 | 🔾 | 🔾 | 🔾 | 🔾 | 🔾 | 🔾 | 🔾 | 🔾 |
| Lamb, Mutton | 🔾 | 🔾 | 🔾 | 🔾 | 🔾 | 🔾 | 🔾 | 🔾 | 🔾 | 🔾 | 🔾 | 🔾 | 🔾 | 🔾 |
| Deer, Caribou, Reindeer | 🔾 | 🔾 | 🔾 | 🔾 | 🔾 | 🔾 | 🔾 | 🔾 | 🔾 | 🔾 | 🔾 | 🔾 | 🔾 | 🔾 |
| Chicken | 🔾 | 🔾 | 🔾 | 🔾 | 🔾 | 🔾 | 🔾 | 🔾 | 🔾 | 🔾 | 🔾 | 🔾 | 🔾 | 🔾 |
| Turkey, Duck, Other Poultry | 🔾 | 🔾 | 🔾 | 🔾 | 🔾 | 🔾 | 🔾 | 🔾 | 🔾 | 🔾 | 🔾 | 🔾 | 🔾 | 🔾 |
| Canned Tuna | 🔾 | 🔾 | 🔾 | 🔾 | 🔾 | 🔾 | 🔾 | 🔾 | 🔾 | 🔾 | 🔾 | 🔾 | 🔾 | 🔾 |
| Fish | 🔾 | 🔾 | 🔾 | 🔾 | 🔾 | 🔾 | 🔾 | 🔾 | 🔾 | 🔾 | 🔾 | 🔾 | 🔾 | 🔾 |
| Shrimp, Lobster | 🔾 | 🔾 | 🔾 | 🔾 | 🔾 | 🔾 | 🔾 | 🔾 | 🔾 | 🔾 | 🔾 | 🔾 | 🔾 | 🔾 |
| Oysters, Shellfish, Scallops | 🔾 | 🔾 | 🔾 | 🔾 | 🔾 | 🔾 | 🔾 | 🔾 | 🔾 | 🔾 | 🔾 | 🔾 | 🔾 | 🔾 |
| Beans | 🔾 | 🔾 | 🔾 | 🔾 | 🔾 | 🔾 | 🔾 | 🔾 | 🔾 | 🔾 | 🔾 | 🔾 | 🔾 | 🔾 |
| Lentils | 🔾 | 🔾 | 🔾 | 🔾 | 🔾 | 🔾 | 🔾 | 🔾 | 🔾 | 🔾 | 🔾 | 🔾 | 🔾 | 🔾 |
| Other Meat (Specify) | 🔾 | 🔾 | 🔾 | 🔾 | 🔾 | 🔾 | 🔾 | 🔾 | 🔾 | 🔾 | 🔾 | 🔾 | 🔾 | 🔾 |

**152) Comments Regarding Protein Sources ____**_________________________________________________

**153) Protein Sources: Estimate how frequently you ate Flame-grilled and Smoked Meats and Meat Products. Give the best estimates you can. Also indicate if you consumed Uncooked Meat, Uncooked Fish, or consumed preparations with Brain, Spinal Tissue and or Eyes.**

|  | Non-MS Subject or Before Onset of MS | | | | | | | | | | | | | |
| --- | --- | --- | --- | --- | --- | --- | --- | --- | --- | --- | --- | --- | --- | --- |
|  | Never | Less than once a month | 1-3 times a month | Once a week | 2-4 times a week | 5-6 times a week | Once a day | 2-3 times a day | 4 or more times a day | Don’t Remember | Not Applicable | Not Applicable at this stage | Other (specify) | Declined |
| Flame Grilled Meat. Include flame-grilled hamburgers | 🔾 | 🔾 | 🔾 | 🔾 | 🔾 | 🔾 | 🔾 | 🔾 | 🔾 | 🔾 | 🔾 | 🔾 | 🔾 | 🔾 |
| Smoked Meats | 🔾 | 🔾 | 🔾 | 🔾 | 🔾 | 🔾 | 🔾 | 🔾 | 🔾 | 🔾 | 🔾 | 🔾 | 🔾 | 🔾 |
| Uncooked Meat | 🔾 | 🔾 | 🔾 | 🔾 | 🔾 | 🔾 | 🔾 | 🔾 | 🔾 | 🔾 | 🔾 | 🔾 | 🔾 | 🔾 |
| Uncooked Fish, Include Sushi | 🔾 | 🔾 | 🔾 | 🔾 | 🔾 | 🔾 | 🔾 | 🔾 | 🔾 | 🔾 | 🔾 | 🔾 | 🔾 | 🔾 |
| Brain | 🔾 | 🔾 | 🔾 | 🔾 | 🔾 | 🔾 | 🔾 | 🔾 | 🔾 | 🔾 | 🔾 | 🔾 | 🔾 | 🔾 |
| Spinal Tissues | 🔾 | 🔾 | 🔾 | 🔾 | 🔾 | 🔾 | 🔾 | 🔾 | 🔾 | 🔾 | 🔾 | 🔾 | 🔾 | 🔾 |
| Eyes | 🔾 | 🔾 | 🔾 | 🔾 | 🔾 | 🔾 | 🔾 | 🔾 | 🔾 | 🔾 | 🔾 | 🔾 | 🔾 | 🔾 |
| Other (Specify) | 🔾 | 🔾 | 🔾 | 🔾 | 🔾 | 🔾 | 🔾 | 🔾 | 🔾 | 🔾 | 🔾 | 🔾 | 🔾 | 🔾 |

|  | After Onset of MS | | | | | | | | | | | | | |
| --- | --- | --- | --- | --- | --- | --- | --- | --- | --- | --- | --- | --- | --- | --- |
|  | Never | Less than once a month | 1-3 times a month | Once a week | 2-4 times a week | 5-6 times a week | Once a day | 2-3 times a day | 4 or more times a day | Don’t Remember | Not Applicable | Not Applicable at this stage | Other (specify) | Declined |
| Flame Grilled Meat. Include flame-grilled hamburgers | 🔾 | 🔾 | 🔾 | 🔾 | 🔾 | 🔾 | 🔾 | 🔾 | 🔾 | 🔾 | 🔾 | 🔾 | 🔾 | 🔾 |
| Smoked Meats | 🔾 | 🔾 | 🔾 | 🔾 | 🔾 | 🔾 | 🔾 | 🔾 | 🔾 | 🔾 | 🔾 | 🔾 | 🔾 | 🔾 |
| Uncooked Meat | 🔾 | 🔾 | 🔾 | 🔾 | 🔾 | 🔾 | 🔾 | 🔾 | 🔾 | 🔾 | 🔾 | 🔾 | 🔾 | 🔾 |
| Uncooked Fish, Include Sushi | 🔾 | 🔾 | 🔾 | 🔾 | 🔾 | 🔾 | 🔾 | 🔾 | 🔾 | 🔾 | 🔾 | 🔾 | 🔾 | 🔾 |
| Brain | 🔾 | 🔾 | 🔾 | 🔾 | 🔾 | 🔾 | 🔾 | 🔾 | 🔾 | 🔾 | 🔾 | 🔾 | 🔾 | 🔾 |
| Spinal Tissues | 🔾 | 🔾 | 🔾 | 🔾 | 🔾 | 🔾 | 🔾 | 🔾 | 🔾 | 🔾 | 🔾 | 🔾 | 🔾 | 🔾 |
| Eyes | 🔾 | 🔾 | 🔾 | 🔾 | 🔾 | 🔾 | 🔾 | 🔾 | 🔾 | 🔾 | 🔾 | 🔾 | 🔾 | 🔾 |
| Other (Specify) | 🔾 | 🔾 | 🔾 | 🔾 | 🔾 | 🔾 | 🔾 | 🔾 | 🔾 | 🔾 | 🔾 | 🔾 | 🔾 | 🔾 |

|  | Last 3-Months | | | | | | | | | | | | | |
| --- | --- | --- | --- | --- | --- | --- | --- | --- | --- | --- | --- | --- | --- | --- |
|  | Never | Less than once a month | 1-3 times a month | Once a week | 2-4 times a week | 5-6 times a week | Once a day | 2-3 times a day | 4 or more times a day | Don’t Remember | Not Applicable | Not Applicable at this stage | Other (specify) | Declined |
| Flame Grilled Meat. Include flame-grilled hamburgers | 🔾 | 🔾 | 🔾 | 🔾 | 🔾 | 🔾 | 🔾 | 🔾 | 🔾 | 🔾 | 🔾 | 🔾 | 🔾 | 🔾 |
| Smoked Meats | 🔾 | 🔾 | 🔾 | 🔾 | 🔾 | 🔾 | 🔾 | 🔾 | 🔾 | 🔾 | 🔾 | 🔾 | 🔾 | 🔾 |
| Uncooked Meat | 🔾 | 🔾 | 🔾 | 🔾 | 🔾 | 🔾 | 🔾 | 🔾 | 🔾 | 🔾 | 🔾 | 🔾 | 🔾 | 🔾 |
| Uncooked Fish, Include Sushi | 🔾 | 🔾 | 🔾 | 🔾 | 🔾 | 🔾 | 🔾 | 🔾 | 🔾 | 🔾 | 🔾 | 🔾 | 🔾 | 🔾 |
| Brain | 🔾 | 🔾 | 🔾 | 🔾 | 🔾 | 🔾 | 🔾 | 🔾 | 🔾 | 🔾 | 🔾 | 🔾 | 🔾 | 🔾 |
| Spinal Tissues | 🔾 | 🔾 | 🔾 | 🔾 | 🔾 | 🔾 | 🔾 | 🔾 | 🔾 | 🔾 | 🔾 | 🔾 | 🔾 | 🔾 |
| Eyes | 🔾 | 🔾 | 🔾 | 🔾 | 🔾 | 🔾 | 🔾 | 🔾 | 🔾 | 🔾 | 🔾 | 🔾 | 🔾 | 🔾 |
| Other (Specify) | 🔾 | 🔾 | 🔾 | 🔾 | 🔾 | 🔾 | 🔾 | 🔾 | 🔾 | 🔾 | 🔾 | 🔾 | 🔾 | 🔾 |

**154) Drinks Consumed: What did you drink? Include Breakfast Drinks such as Orange Drinks, Coffee, Tea etc., Water consumed throughout the day, Wine, Tea, Colas etc. Do NOT Include Dairy, e.g., Milk, Yogurt, Shakes.**

|  | Non-MS Subject or Before Onset of MS | | | | | | | | | | | | | |
| --- | --- | --- | --- | --- | --- | --- | --- | --- | --- | --- | --- | --- | --- | --- |
|  | Never | Less than once a month | 1-3 times a month | Once a week | 2-4 times a week | 5-6 times a week | Once a day | 2-3 times a day | 4 or more times a day | Don’t Remember | Not Applicable | Not Applicable at this stage | Other (specify) | Declined |
| Water or bottled water (1 cup or Bottle) | 🔾 | 🔾 | 🔾 | 🔾 | 🔾 | 🔾 | 🔾 | 🔾 | 🔾 | 🔾 | 🔾 | 🔾 | 🔾 | 🔾 |
| Regular soda/pop (1 can) | 🔾 | 🔾 | 🔾 | 🔾 | 🔾 | 🔾 | 🔾 | 🔾 | 🔾 | 🔾 | 🔾 | 🔾 | 🔾 | 🔾 |
| Diet soda/pop (1 can) | 🔾 | 🔾 | 🔾 | 🔾 | 🔾 | 🔾 | 🔾 | 🔾 | 🔾 | 🔾 | 🔾 | 🔾 | 🔾 | 🔾 |
| Fruit juices (1 cup) | 🔾 | 🔾 | 🔾 | 🔾 | 🔾 | 🔾 | 🔾 | 🔾 | 🔾 | 🔾 | 🔾 | 🔾 | 🔾 | 🔾 |
| Red wine (1 cup) | 🔾 | 🔾 | 🔾 | 🔾 | 🔾 | 🔾 | 🔾 | 🔾 | 🔾 | 🔾 | 🔾 | 🔾 | 🔾 | 🔾 |
| White wine | 🔾 | 🔾 | 🔾 | 🔾 | 🔾 | 🔾 | 🔾 | 🔾 | 🔾 | 🔾 | 🔾 | 🔾 | 🔾 | 🔾 |
| Wine cooler, cocktail, liquor, liqueur | 🔾 | 🔾 | 🔾 | 🔾 | 🔾 | 🔾 | 🔾 | 🔾 | 🔾 | 🔾 | 🔾 | 🔾 | 🔾 | 🔾 |
| Beer (1 can) | 🔾 | 🔾 | 🔾 | 🔾 | 🔾 | 🔾 | 🔾 | 🔾 | 🔾 | 🔾 | 🔾 | 🔾 | 🔾 | 🔾 |
| Black tea | 🔾 | 🔾 | 🔾 | 🔾 | 🔾 | 🔾 | 🔾 | 🔾 | 🔾 | 🔾 | 🔾 | 🔾 | 🔾 | 🔾 |
| Green tea | 🔾 | 🔾 | 🔾 | 🔾 | 🔾 | 🔾 | 🔾 | 🔾 | 🔾 | 🔾 | 🔾 | 🔾 | 🔾 | 🔾 |
| Regular coffee | 🔾 | 🔾 | 🔾 | 🔾 | 🔾 | 🔾 | 🔾 | 🔾 | 🔾 | 🔾 | 🔾 | 🔾 | 🔾 | 🔾 |
| Decaffeinated coffee | 🔾 | 🔾 | 🔾 | 🔾 | 🔾 | 🔾 | 🔾 | 🔾 | 🔾 | 🔾 | 🔾 | 🔾 | 🔾 | 🔾 |
| Soy Milk | 🔾 | 🔾 | 🔾 | 🔾 | 🔾 | 🔾 | 🔾 | 🔾 | 🔾 | 🔾 | 🔾 | 🔾 | 🔾 | 🔾 |
| Other (Specify) | 🔾 | 🔾 | 🔾 | 🔾 | 🔾 | 🔾 | 🔾 | 🔾 | 🔾 | 🔾 | 🔾 | 🔾 | 🔾 | 🔾 |

|  | After Onset of MS | | | | | | | | | | | | | |
| --- | --- | --- | --- | --- | --- | --- | --- | --- | --- | --- | --- | --- | --- | --- |
|  | Never | Less than once a month | 1-3 times a month | Once a week | 2-4 times a week | 5-6 times a week | Once a day | 2-3 times a day | 4 or more times a day | Don’t Remember | Not Applicable | Not Applicable at this stage | Other (specify) | Declined |
| Water or bottled water (1 cup or Bottle) | 🔾 | 🔾 | 🔾 | 🔾 | 🔾 | 🔾 | 🔾 | 🔾 | 🔾 | 🔾 | 🔾 | 🔾 | 🔾 | 🔾 |
| Regular soda/pop (1 can) | 🔾 | 🔾 | 🔾 | 🔾 | 🔾 | 🔾 | 🔾 | 🔾 | 🔾 | 🔾 | 🔾 | 🔾 | 🔾 | 🔾 |
| Diet soda/pop (1 can) | 🔾 | 🔾 | 🔾 | 🔾 | 🔾 | 🔾 | 🔾 | 🔾 | 🔾 | 🔾 | 🔾 | 🔾 | 🔾 | 🔾 |
| Fruit juices (1 cup) | 🔾 | 🔾 | 🔾 | 🔾 | 🔾 | 🔾 | 🔾 | 🔾 | 🔾 | 🔾 | 🔾 | 🔾 | 🔾 | 🔾 |
| Red wine (1 cup) | 🔾 | 🔾 | 🔾 | 🔾 | 🔾 | 🔾 | 🔾 | 🔾 | 🔾 | 🔾 | 🔾 | 🔾 | 🔾 | 🔾 |
| White wine | 🔾 | 🔾 | 🔾 | 🔾 | 🔾 | 🔾 | 🔾 | 🔾 | 🔾 | 🔾 | 🔾 | 🔾 | 🔾 | 🔾 |
| Wine cooler, cocktail, liquor, liqueur | 🔾 | 🔾 | 🔾 | 🔾 | 🔾 | 🔾 | 🔾 | 🔾 | 🔾 | 🔾 | 🔾 | 🔾 | 🔾 | 🔾 |
| Beer (1 can) | 🔾 | 🔾 | 🔾 | 🔾 | 🔾 | 🔾 | 🔾 | 🔾 | 🔾 | 🔾 | 🔾 | 🔾 | 🔾 | 🔾 |
| Black tea | 🔾 | 🔾 | 🔾 | 🔾 | 🔾 | 🔾 | 🔾 | 🔾 | 🔾 | 🔾 | 🔾 | 🔾 | 🔾 | 🔾 |
| Green tea | 🔾 | 🔾 | 🔾 | 🔾 | 🔾 | 🔾 | 🔾 | 🔾 | 🔾 | 🔾 | 🔾 | 🔾 | 🔾 | 🔾 |
| Regular coffee | 🔾 | 🔾 | 🔾 | 🔾 | 🔾 | 🔾 | 🔾 | 🔾 | 🔾 | 🔾 | 🔾 | 🔾 | 🔾 | 🔾 |
| Decaffeinated coffee | 🔾 | 🔾 | 🔾 | 🔾 | 🔾 | 🔾 | 🔾 | 🔾 | 🔾 | 🔾 | 🔾 | 🔾 | 🔾 | 🔾 |
| Soy Milk | 🔾 | 🔾 | 🔾 | 🔾 | 🔾 | 🔾 | 🔾 | 🔾 | 🔾 | 🔾 | 🔾 | 🔾 | 🔾 | 🔾 |
| Other (Specify) | 🔾 | 🔾 | 🔾 | 🔾 | 🔾 | 🔾 | 🔾 | 🔾 | 🔾 | 🔾 | 🔾 | 🔾 | 🔾 | 🔾 |

|  | Last 3-Months? | | | | | | | | | | | | | |
| --- | --- | --- | --- | --- | --- | --- | --- | --- | --- | --- | --- | --- | --- | --- |
|  | Never | Less than once a month | 1-3 times a month | Once a week | 2-4 times a week | 5-6 times a week | Once a day | 2-3 times a day | 4 or more times a day | Don’t Remember | Not Applicable | Not Applicable at this stage | Other (specify) | Declined |
| Water or bottled water (1 cup or Bottle) | 🔾 | 🔾 | 🔾 | 🔾 | 🔾 | 🔾 | 🔾 | 🔾 | 🔾 | 🔾 | 🔾 | 🔾 | 🔾 | 🔾 |
| Regular soda/pop (1 can) | 🔾 | 🔾 | 🔾 | 🔾 | 🔾 | 🔾 | 🔾 | 🔾 | 🔾 | 🔾 | 🔾 | 🔾 | 🔾 | 🔾 |
| Diet soda/pop (1 can) | 🔾 | 🔾 | 🔾 | 🔾 | 🔾 | 🔾 | 🔾 | 🔾 | 🔾 | 🔾 | 🔾 | 🔾 | 🔾 | 🔾 |
| Fruit juices (1 cup) | 🔾 | 🔾 | 🔾 | 🔾 | 🔾 | 🔾 | 🔾 | 🔾 | 🔾 | 🔾 | 🔾 | 🔾 | 🔾 | 🔾 |
| Red wine (1 cup) | 🔾 | 🔾 | 🔾 | 🔾 | 🔾 | 🔾 | 🔾 | 🔾 | 🔾 | 🔾 | 🔾 | 🔾 | 🔾 | 🔾 |
| White wine | 🔾 | 🔾 | 🔾 | 🔾 | 🔾 | 🔾 | 🔾 | 🔾 | 🔾 | 🔾 | 🔾 | 🔾 | 🔾 | 🔾 |
| Wine cooler, cocktail, liquor, liqueur | 🔾 | 🔾 | 🔾 | 🔾 | 🔾 | 🔾 | 🔾 | 🔾 | 🔾 | 🔾 | 🔾 | 🔾 | 🔾 | 🔾 |
| Beer (1 can) | 🔾 | 🔾 | 🔾 | 🔾 | 🔾 | 🔾 | 🔾 | 🔾 | 🔾 | 🔾 | 🔾 | 🔾 | 🔾 | 🔾 |
| Black tea | 🔾 | 🔾 | 🔾 | 🔾 | 🔾 | 🔾 | 🔾 | 🔾 | 🔾 | 🔾 | 🔾 | 🔾 | 🔾 | 🔾 |
| Green tea | 🔾 | 🔾 | 🔾 | 🔾 | 🔾 | 🔾 | 🔾 | 🔾 | 🔾 | 🔾 | 🔾 | 🔾 | 🔾 | 🔾 |
| Regular coffee | 🔾 | 🔾 | 🔾 | 🔾 | 🔾 | 🔾 | 🔾 | 🔾 | 🔾 | 🔾 | 🔾 | 🔾 | 🔾 | 🔾 |
| Decaffeinated coffee | 🔾 | 🔾 | 🔾 | 🔾 | 🔾 | 🔾 | 🔾 | 🔾 | 🔾 | 🔾 | 🔾 | 🔾 | 🔾 | 🔾 |
| Soy Milk | 🔾 | 🔾 | 🔾 | 🔾 | 🔾 | 🔾 | 🔾 | 🔾 | 🔾 | 🔾 | 🔾 | 🔾 | 🔾 | 🔾 |
| Other (Specify) | 🔾 | 🔾 | 🔾 | 🔾 | 🔾 | 🔾 | 🔾 | 🔾 | 🔾 | 🔾 | 🔾 | 🔾 | 🔾 | 🔾 |

**155) Water: Have you ever used Non-municipal Well, Lake, Pond Water as Your Primary Source of Water for Drinking? This could occur in Rural Areas, Farms, vacations or stays in Developing Countries.**

|  | Non-MS Subject or Before Onset of MS | | | | | | | | | | |
| --- | --- | --- | --- | --- | --- | --- | --- | --- | --- | --- | --- |
|  | No | Yes, for less than 1 month | Yes, for 1-6 months | Yes, for 6-11 months | Yes, for 1 year | Yes, for 2-3 years | Yes, for 3-5 years | Yes, More than 5 years | Do not Remember | Do not Know | Declined |
| Non-Municipal Well Water | 🔾 | 🔾 | 🔾 | 🔾 | 🔾 | 🔾 | 🔾 | 🔾 | 🔾 | 🔾 | 🔾 |
| Non-Municipal Lake or Pond Water | 🔾 | 🔾 | 🔾 | 🔾 | 🔾 | 🔾 | 🔾 | 🔾 | 🔾 | 🔾 | 🔾 |
| Non-Municipal River Water | 🔾 | 🔾 | 🔾 | 🔾 | 🔾 | 🔾 | 🔾 | 🔾 | 🔾 | 🔾 | 🔾 |

|  | After Onset of MS | | | | | | | | | | |
| --- | --- | --- | --- | --- | --- | --- | --- | --- | --- | --- | --- |
|  | No | Yes, for less than 1 month | Yes, for 1-6 months | Yes, for 6-11 months | Yes, for 1 year | Yes, for 2-3 years | Yes, for 3-5 years | Yes, More than 5 years | Do not Remember | Do not Know | Declined |
| Non-Municipal Well Water | 🔾 | 🔾 | 🔾 | 🔾 | 🔾 | 🔾 | 🔾 | 🔾 | 🔾 | 🔾 | 🔾 |
| Non-Municipal Lake or Pond Water | 🔾 | 🔾 | 🔾 | 🔾 | 🔾 | 🔾 | 🔾 | 🔾 | 🔾 | 🔾 | 🔾 |
| Non-Municipal River Water | 🔾 | 🔾 | 🔾 | 🔾 | 🔾 | 🔾 | 🔾 | 🔾 | 🔾 | 🔾 | 🔾 |

|  | Last 3 Months? | | | | | | | | | | |
| --- | --- | --- | --- | --- | --- | --- | --- | --- | --- | --- | --- |
|  | No | Yes, for less than 1 month | Yes, for 1-6 months | Yes, for 6-11 months | Yes, for 1 year | Yes, for 2-3 years | Yes, for 3-5 years | Yes, More than 5 years | Do not Remember | Do not Know | Declined |
| Non-Municipal Well Water | 🔾 | 🔾 | 🔾 | 🔾 | 🔾 | 🔾 | 🔾 | 🔾 | 🔾 | 🔾 | 🔾 |
| Non-Municipal Lake or Pond Water | 🔾 | 🔾 | 🔾 | 🔾 | 🔾 | 🔾 | 🔾 | 🔾 | 🔾 | 🔾 | 🔾 |
| Non-Municipal River Water | 🔾 | 🔾 | 🔾 | 🔾 | 🔾 | 🔾 | 🔾 | 🔾 | 🔾 | 🔾 | 🔾 |

**156) Comments Regarding Drinks and Water _____________________________________________**

**157) Dairy and Dairy Products Consumed: Dairy includes, Milk and Milk Products such as Ice cream, Cheese, Yogurt, Sour Cream.**

|  | Non-MS Subject or Before Onset of MS | | | | | | | | | | | | | |
| --- | --- | --- | --- | --- | --- | --- | --- | --- | --- | --- | --- | --- | --- | --- |
|  | Never | Less than once a month | 1-3 times a month | Once a week | 2-4 times a week | 5-6 times a week | Once a day | 2-3 times a day | 4 or more times a day | Don’t Remember | Not Applicable | Not Applicable at this stage | Other (specify) | Declined |
| `Whole or Homogenized Milk | 🔾 | 🔾 | 🔾 | 🔾 | 🔾 | 🔾 | 🔾 | 🔾 | 🔾 | 🔾 | 🔾 | 🔾 | 🔾 | 🔾 |
| Low Fat Milk | 🔾 | 🔾 | 🔾 | 🔾 | 🔾 | 🔾 | 🔾 | 🔾 | 🔾 | 🔾 | 🔾 | 🔾 | 🔾 | 🔾 |
| Non-Fat Milk | 🔾 | 🔾 | 🔾 | 🔾 | 🔾 | 🔾 | 🔾 | 🔾 | 🔾 | 🔾 | 🔾 | 🔾 | 🔾 | 🔾 |
| Sliced cheese (include sandwiches) | 🔾 | 🔾 | 🔾 | 🔾 | 🔾 | 🔾 | 🔾 | 🔾 | 🔾 | 🔾 | 🔾 | 🔾 | 🔾 | 🔾 |
| Whole Cheese | 🔾 | 🔾 | 🔾 | 🔾 | 🔾 | 🔾 | 🔾 | 🔾 | 🔾 | 🔾 | 🔾 | 🔾 | 🔾 | 🔾 |
| Yogurt, Yogurt Drinks | 🔾 | 🔾 | 🔾 | 🔾 | 🔾 | 🔾 | 🔾 | 🔾 | 🔾 | 🔾 | 🔾 | 🔾 | 🔾 | 🔾 |
| Ice cream, Milk Shakes | 🔾 | 🔾 | 🔾 | 🔾 | 🔾 | 🔾 | 🔾 | 🔾 | 🔾 | 🔾 | 🔾 | 🔾 | 🔾 | 🔾 |
| Cream, Sour Cream | 🔾 | 🔾 | 🔾 | 🔾 | 🔾 | 🔾 | 🔾 | 🔾 | 🔾 | 🔾 | 🔾 | 🔾 | 🔾 | 🔾 |
| Other (Specify) | 🔾 | 🔾 | 🔾 | 🔾 | 🔾 | 🔾 | 🔾 | 🔾 | 🔾 | 🔾 | 🔾 | 🔾 | 🔾 | 🔾 |

|  | After Onset of MS | | | | | | | | | | | | | |
| --- | --- | --- | --- | --- | --- | --- | --- | --- | --- | --- | --- | --- | --- | --- |
|  | Never | Less than once a month | 1-3 times a month | Once a week | 2-4 times a week | 5-6 times a week | Once a day | 2-3 times a day | 4 or more times a day | Don’t Remember | Not Applicable | Not Applicable at this stage | Other (specify) | Declined |
| `Whole or Homogenized Milk | 🔾 | 🔾 | 🔾 | 🔾 | 🔾 | 🔾 | 🔾 | 🔾 | 🔾 | 🔾 | 🔾 | 🔾 | 🔾 | 🔾 |
| Low Fat Milk | 🔾 | 🔾 | 🔾 | 🔾 | 🔾 | 🔾 | 🔾 | 🔾 | 🔾 | 🔾 | 🔾 | 🔾 | 🔾 | 🔾 |
| Non-Fat Milk | 🔾 | 🔾 | 🔾 | 🔾 | 🔾 | 🔾 | 🔾 | 🔾 | 🔾 | 🔾 | 🔾 | 🔾 | 🔾 | 🔾 |
| Sliced cheese (include sandwiches) | 🔾 | 🔾 | 🔾 | 🔾 | 🔾 | 🔾 | 🔾 | 🔾 | 🔾 | 🔾 | 🔾 | 🔾 | 🔾 | 🔾 |
| Whole Cheese | 🔾 | 🔾 | 🔾 | 🔾 | 🔾 | 🔾 | 🔾 | 🔾 | 🔾 | 🔾 | 🔾 | 🔾 | 🔾 | 🔾 |
| Yogurt, Yogurt Drinks | 🔾 | 🔾 | 🔾 | 🔾 | 🔾 | 🔾 | 🔾 | 🔾 | 🔾 | 🔾 | 🔾 | 🔾 | 🔾 | 🔾 |
| Ice cream, Milk Shakes | 🔾 | 🔾 | 🔾 | 🔾 | 🔾 | 🔾 | 🔾 | 🔾 | 🔾 | 🔾 | 🔾 | 🔾 | 🔾 | 🔾 |
| Cream, Sour Cream | 🔾 | 🔾 | 🔾 | 🔾 | 🔾 | 🔾 | 🔾 | 🔾 | 🔾 | 🔾 | 🔾 | 🔾 | 🔾 | 🔾 |
| Other (Specify) | 🔾 | 🔾 | 🔾 | 🔾 | 🔾 | 🔾 | 🔾 | 🔾 | 🔾 | 🔾 | 🔾 | 🔾 | 🔾 | 🔾 |

**158) Comments Regarding Dairy _________________**____________________________________________
