# Supplementary material for: Risk Factors for Chronic Cerebrospinal Venous Insufficiency (CCSVI) in a Large Cohort of Volunteers
Source: PLoS One. 2011 Nov 30;6(11):e28062. doi: 10.1371/journal.pone.0028062 (PMC3227626; doi:10.1371/journal.pone.0028062)
Supplement: Appendix S2 — Environmental Factors in Multiple Sclerosis. (DOC) [file pone.0028062.s002.doc]

**Appendix S2: Environmental Factors in Multiple Sclerosis (short list)**

All the subjects were interviewed using structured questionnaire with questions that included information about:

**1. Demographics characteristics:**

- *age*
- *gender*
- *weight and height*
- *birthplace (city, state and country)*
- *parents' and siblings' place of birth*
- *ethnicity (US Census Bureau)*
- *familiarity with multiple sclerosis*
- *pregnancies and live births*

**2. Vascular risk factors**:

- *presence of heart disease* that included: congestive heart failure, heart attack, arrhythmia, valvular disease, heart murmurs, enlarged heart, heart surgery, rheumatic fever, constrictive pericarditis, pulmonary hypertension, mediastinal tumor and other
- *hypertension*
- *diabetes*
- *obesity:* body mass index (BMI) > 30; body shape
- *smoking:* passive vs. active, duration, frequency, number of cigarettes per day
- *high jugular venous pressure*

**3. Environmental factors**:

- *race (US Census Bureau)*
- *education status*
- *living status*
- *employment status:* current employment status, job title, job activities, city, state
- *allergies*: to foods (eggs, milk, peanuts, tree nuts, wheat, soy, fish, shellfish), animals (cat), insects (bee sting, mosquito sting, wasp sting), plant pollens, grass, tree, weeds, mold, latex, metals and solvents
- *vaccines*: Tetanus-Diphtheria-Pertussis (TDP) vaccine, Hepatitis A vaccine, Hepatitis B vaccine, HiB (H. influenzae), Influenza vaccine, Measles-Mumps-Rubella (MMR vaccine), Polio (inactivated vaccine), Pneumococcal vaccine, Rotavirus vaccine, Varicella (chicken pox) vaccine, Human papillomavirus vaccine (for girls, mark not applicable for males), Meningococcus vaccine and Tuberculosis vaccine
- *extreme physical conditions* on a regular basis
- *stressful and tragic events* (divorce of parents, loss of parent, loss of newborn child, first trimester miscarriages, second or third trimester miscarriages, loss of child under 10 years, loss of child 10-18 years, loss of adult child, loss of sibling, homelessness, bankruptcy, job loss, homicide in home, life threatening illness in family, criminal legal proceedings, civil legal proceedings and prison sentence)
- *color of eyes, natural color of hair*
- *sun exposure* (freckles on unexposed area of skin, brown within several hours of sun exposure, face reaction to sun exposure, job-related sun exposure, exercise-related sun exposure, leisure-related sun exposure, beach, vacation-related exposure, sunbathing and tanning bed)
- *parasitic infections* during life (amoebiasis, coccidia, giardia, Head, body, or crab louse, Liver fluke, Scabies, Strongyloidiasis, Tapeworm, Toxocariasis, Toxoplasmosis, Trichinosis, African trypanosomiasis, Chagas disease, Dengue fever, Leishmaniasis, Malaria, Schistosomiasis and Trachoma
- *contact with pet animals* for three months or more
- *contact with chemicals* on a regular basis
- *contact with farm animals* for more than three months (cows, buffalo, cattle, pigs, swine, chickens, hens, horses)
- *contact with tissues, pathogens, meat* (not for consumption), blood, feces for more than three months as part of the job or otherwise

**4. Environmental factors**:

- *use of alcohol:* frequency, duration, type
- *use of dietary supplements* for more than one month such as: vitamins (B 1,3,5 and 12, C, D, E, K), multivitamin tablets, calcium, fish oil tablets, creatinine, iron and magnesium supplements, B-complex, amino acids and zinc
- *use of herbal supplements* for more than one month such as: acai berry, aloe vera extract, bee pollen, cranberry fruit extract, Echinacea, fenugreek, garlic supplements, ginkgo, green tea extract, kava, peppermint, saw palmetto, red yeast rice, rosehip, sea kelp, soy estrogens, stevia extract; herbal combinations for appetite control, fatigue control, menstrual symptoms, strength muscle-building and libido
- *use of milk products*
- *use of peanut oil:* sesame oil, butter, coconut oil, clarified butter, olive oil, corn oil, sunflower oil, lard, fish oil and mustard or rapeseed oil
- *physical activity* (frequency and type): mixed exercises, cross training, yoga, Pilates, strength training, Tai Chi, aerobics exercises, running, walking, swimming, court sports (tennis, basketball, racquetball, squash), field sport (soccer, lacrosse, field hockey), contact field sport (rugby, football), boxing, contact sport (wrestling, judo) and TV exercises
- *sleeping:* hours of sleep, daytime sleep or nap, sleeping side, extra pillows
- *use of illicit drugs* such as: marijuana, hashish, heroin, cocaine, amphetamines, Adderall, Benzedrine, Metamphetamine, Speed, Dexies, Preludin, Yellow Jackets, Epitestosterone, Erythropoeitin, EPO, Epoidin, Ecstasy, MDMA, Molly, Inhalants or solvents (not Nitrites), Prescription opiates and painkillers, Human growth hormone, Amyl nitrite, butyl nitrite, Rush, Nitrous oxide, whip-its and other prescription drugs (Ritalin, etc.)

**5**. **Autoimmune diseases**:

- *SLE*
- *rheumatoid disorders*
- *Crohn's disease*
- *psoriasis*
- *myasthenia gravis*
- *diabetes type 1*

**6**. **Other concomitant diseases**:

- *asthma*
- *allergy*
- *cancer*
- *lymphoma*
- *chronic opstructive pulmonary disease*
- *irritable bowel syndrome*
- *migraine*
